# Novel potential drugs for the treatment of primary open-angle glaucoma using protein-protein interaction network analysis

**DOI:** 10.5808/gi.22070

**Published:** 2023-03-31

**Authors:** Parisima Ghaffarian Zavarzadeh, Zahra Abedi

**Affiliations:** 1 Laboratory of Systems Biology and Bioinformatics (LBB), Institute of Biochemistry and Biophysics, University of Tehran, Tehran 1417935840, Iran; 2 Luxembourg Centre for Systems Biomedicine (LCSB), University of Luxembourg, Belvaux 4365, Luxembourg

**Keywords:** differentially expressed genes, drug-gene network, drug repurposing, glaucoma, primary open-angle, protein-protein interaction

## Abstract

Glaucoma is the second leading cause of irreversible blindness, and primary open-angle glaucoma (POAG) is the most common type. Due to inadequate diagnosis, treatment is often not administered until symptoms occur. Hence, approaches enabling earlier prediction or diagnosis of POAG are necessary. We aimed to identify novel drugs for glaucoma through bioinformatics and network analysis. Data from 36 samples, obtained from the trabecular meshwork of healthy individuals and patients with POAG, were acquired from a dataset. Next, differentially expressed genes (DEGs) were identified to construct a protein-protein interaction (PPI) network. In both stages, the genes were enriched by studying the critical biological processes and pathways related to POAG. Finally, a drug-gene network was constructed, and novel drugs for POAG treatment were proposed. Genes with p < 0.01 and |log fold change| > 0.3 (1,350 genes) were considered DEGs and utilized to construct a PPI network. Enrichment analysis yielded several key pathways that were upregulated or downregulated. For example, extracellular matrix organization, the immune system, neutrophil degranulation, and cytokine signaling were upregulated among immune pathways, while signal transduction, the immune system, extracellular matrix organization, and receptor tyrosine kinase signaling were downregulated. Finally, novel drugs including metformin hydrochloride, ixazomib citrate, and cisplatin warrant further analysis of their potential roles in POAG treatment. The candidate drugs identified in this computational analysis require *in vitro* and *in vivo* validation to confirm their effectiveness in POAG treatment. This may pave the way for understanding life-threatening disorders such as cancer.

## Introduction

Glaucoma is the second leading cause of irreversible blindness worldwide [1-3]. It is primarily characterized by progressive optic nerve degeneration with elevated intraocular pressure (IOP) leading to eyesight impairment [1]. Two major types, primary open-angle glaucoma (POAG) and angle-closure glaucoma (PCAG), are distinguished by the anatomic configuration of the aqueous humor outflow pathway [4]. Among the various types of glaucoma, POAG is the most common, affecting 60%–70% of patients [1]. Similar to other types, POAG progresses quietly, and blindness is the final stage of the disease; approximately 50% of glaucoma cases are not diagnosed until irreversible damage has occurred [1]. Epidemiologically, the majority of POAG cases are in the United States and Western Europe, while PCAG is mainly observed among Chinese and other Asian people [4].

Major risk factors for POAG include elevated IOP, older age, African ethnic origin, family history of glaucoma, and myopia. Less pivotal risk factors in the progression of glaucoma include cardiovascular risk factors such as dyslipidemia, hypertension, diabetes mellitus, and high body mass index or obesity [2]. Since no neuroprotective treatments are available, therapies are mainly limited to lowering IOP; therefore, system biology and computational analysis could pave the way for improved diagnosis and hinder the progression of this and similar incurable disorders [5]. Three main treatment approaches are available for glaucoma: laser treatment, incisional surgery, and medication [6]. Additionally, diet plays a role in preventing and controlling POAG, with research indicating that consumption of specific foods is directly linked with the incidence and progression of glaucoma via impact on IOP [7]. IOP-lowering medications have long been effective in controlling IOP in patients with glaucoma. Prostaglandin analogs are considered the gold standard treatment for POAG based on their efficiency and safety [8]. Moreover, other candidate drugs with IOP-lowering effects are being investigated for the potential treatment of glaucoma. However, as science has progressed, the methods of study for such diseases have broadened enormously. For instance, the number of clinical trials on repurposed candidate drugs has increased over recent decades [6]. This approach paves the way for understanding the role of drugs obtained from computational analysis. Notably, few studies have been dedicated to POAG biomarker identification and analysis and computational investigation [1].

Protein-protein interaction (PPI) network analysis is performed to extract the essential sub-modules from the PPI network. Integrated computational analysis has been used to introduce novel potential drugs for the treatment of age-related macular degeneration [9]. However, computational and bioinformatics studies are insufficient; instead, substantial additional clinical and laboratory-based research is required to understand the exact role of each network and proposed drugs in the disease. This study was carried out to analyze microarray transcriptome data that included samples from patients with POAG and a healthy group. We conducted functional annotation and bioinformatics analysis to identify differentially expressed genes (DEGs), and then constructed a PPI network to further evaluate the pathogenesis of and candidate genes for glaucoma. Lastly, we constructed a drug-gene network based on genes in the top module and suggested novel drugs that may contribute to POAG treatment. However, further medical and clinical investigations are required to confirm the roles of these proposed drugs.

## Methods

### Dataset and preprocessing

Expression data with the accession number GSE27276 were downloaded from the National Center for Biotechnology Information Gene Expression Omnibus (https://www.ncbi.nlm.nih.gov/geo/). The samples, all obtained from trabecular meshwork tissue, included 17 from patients with POAG and 19 control samples (36 samples total) [10]. The chip analyzer platform was GPL2507 Sentrix Human-6 Expression BeadChip (Illumina, San Diego, CA, USA) [10]. Before preprocessing, the non-gene transcripts were eliminated from the original file. Then, DEGs between control and disease samples were identified using the Limma package of R (version 4.0.2, R Foundation for Statistical Computing, Vienna, Austria) from the Bioconductor project [11]. Benjamini and Hochberg’s false discovery rate approach was utilized to adjust the p-values. Next, after removal of genes without IDs from the file, the genes were mapped to their IDs, and genes with p < 0.01 and |log fold change| > 0.3 were considered significant and included in further analysis. Then, these genes were used to construct the PPI network through the STRING database (version 11.0; https://string-db.org/) [12].

### Functional enrichment analysis of DEGs

Additionally, a bioinformatics tool was utilized for functional enrichment and pathway analysis of DEGs obtained by gene ontology and Reactome pathways in the DAVID database (https://david.ncifcrf.gov/), respectively [13]. Accordingly, disease-related biological processes and pathways were obtained at this stage.

### PPI network construction

As previously mentioned, the STRING database K-means method (confidence level, 0.7) was used to construct the PPI network from DEGs with p < 0.01 and |log fold change| > 0.3. To visualize the data, Cytoscape version 3.8.2 (The Cytoscape Consortium, San Diego, CA, USA) was utilized. ClusterVis (Sequentix, Berlin, Germany) was used to perform cluster analysis. Different algorithms exist for cluster analysis using ClusterVis. We selected the fast agglomerate edge clustering algorithm in this study. In this algorithm, a coefficient is calculated for each edge in the networks; then, based on the clustering coefficients, the edges are sorted in a decreasing way. Next, protein complexes are identified according to the bottom-up condensation of the hierarchical clustering algorithm. Used to analyze large protein networks, the fast agglomerate edge clustering algorithm is beneficial because of its fast computational power and low complexity [14]. The parameters selected in this study were DefinitionWay: strong, In/OutThreshold: 7, and Overlapped: true. Three PPI clusters (that is, PPI complexes) were detected.

### Functional enrichment and enrichment analysis of top modules

Then, further enrichment analysis was conducted for the top three modules for gene ontology and Reactome pathways (both performed with DAVID v.6.8).

### Drug-gene network construction

After selection of the significant subnetworks, the drug-gene network was constructed. The Drug Gene Interaction Database (DGIdb) (https://www.dgidb.org/) was utilized to identify potential candidate drugs [15]. This comprehensive database includes drug-gene interaction data from six databases (My Cancer Genome 39, TALC 40, TEND 41, PharmGKB 42, TTD43, and DrugBank 44). The candidate drugs are potential treatments for POAG; however, they must be investigated through clinical and laboratory procedures.

## Results

DEGs for control and POAG samples were obtained. The GSE27276 microarray dataset was analyzed to establish a list of important genes involved in POAG pathogenesis. We obtained 1,350 significant DEGs (p < 0.01 and |log fold change| > 0.3) by comparing gene expression levels in POAG samples with controls (Supplementary Table 1).

### Enrichment analysis of DEGs

#### Upregulated genes

The significant pathways and functional enrichment information were extracted from the DAVID database. On the DEG list, the pathways of upregulated genes with p < 0.01 were selected as significant. The most significant pathways or systems were extracellular matrix (ECM) organization, the immune system, integrin cell surface interactions, diseases of metabolism, regulation of insulin-like growth factor transport and uptake by insulin-like growth factor binding proteins, post-translational protein phosphorylation, the adaptive immune system, and signaling by platelet-derived growth factor (PDGF); many other pathways showed significant results as well (Supplementary Table 2). The results of the functional enrichment analysis are provided in Supplementary Tables 3–5.

#### Downregulated genes

A similar approach was utilized to study the significantly downregulated pathways, which included signal transduction, the immune system, ECM organization, cell junction organization, TP53 regulation of transcription of additional cell death genes with uncertain roles in p53-dependent apoptosis, toll-like receptor cascades, and many other pathways (Supplementary Table S6). The results of functional enrichment analysis are provided in Supplementary Tables 7–9. Notably, most of these pathways were related to the immune system.

### PPI network and clustering analysis

**Fig. 1.**
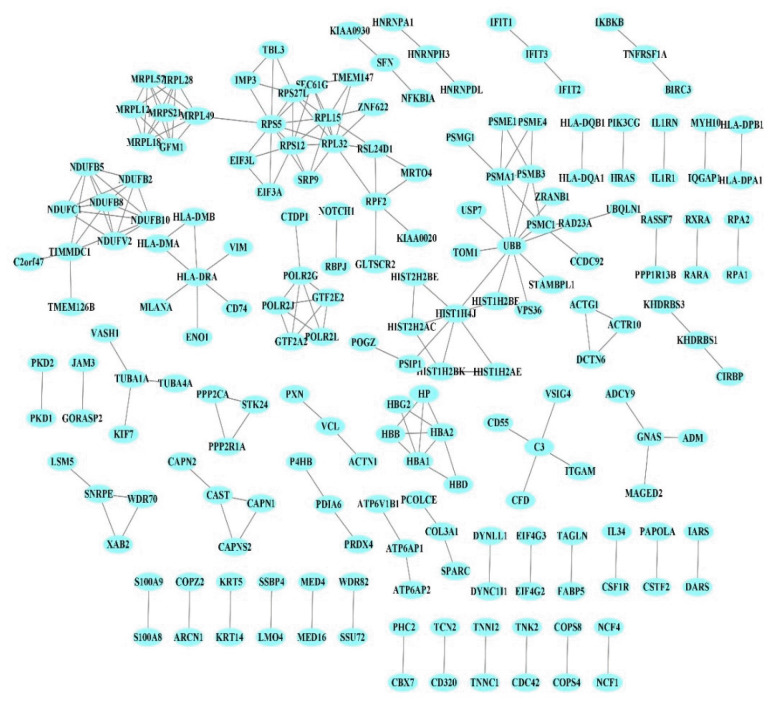
Protein-protein interaction. Each node corresponds to a protein. Edges indicate experimentally validated physical interactions.

**Fig. 2.**
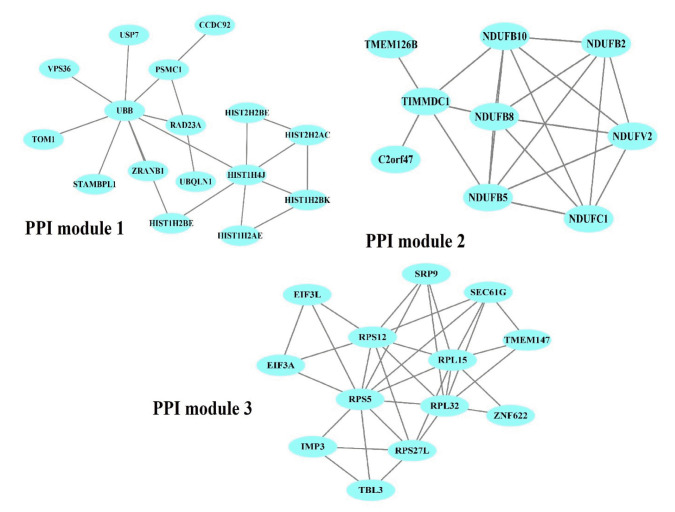
Obtained protein-protein interaction (PPI) modules from the PPI network. Blue ellipses denote PPIs in each module.

The PPI network of 1,350 genes was derived from the STRING database (Fig. 1). Clustering analysis was utilized to find strongly interacting protein modules. Next, three protein modules were established (Fig. 2). Additional analysis of PPI modules was conducted with ClusterVis (see Table 1 for more information).

### Functional annotation and pathway enrichment analysis of PPI modules

The DAVID database was used to perform an enrichment analysis of the genes in each module separately. Based on the Reactome pathway database, the most significant pathways of module 1 (p < 0.01) were post-translational protein modification, nucleotide excision repair, and PTEN regulation (Supplementary Table 10). For module 2, Reactome pathway enrichment revealed pathways including complex I biogenesis, respiratory electron transport, ATP synthesis by chemiosmotic coupling, and heat production by uncoupling proteins and metabolism (p < 0.01) (Supplementary Table 11). In contrast, the Reactome pathways for module 3 were translation ribosomal RNA processing in the nucleus and cytosol, nonsense-mediated decay, regulation of expression of Slits and Robos, protein metabolism, nervous system development, cellular responses to stress, and cellular responses to stimuli (p < 0.01) (Supplementary Table 12).

The results of the gene ontology enrichment analysis of each module, including significant (p < 0.01) biological processes, molecular functions, and cell components, are indicated in Supplementary Tables 13–15 (module 1), Supplementary Tables 16–18 (module 2), and Supplementary Tables 19–21 (module 3).

### Drug-gene interaction network

**Fig. 3.**
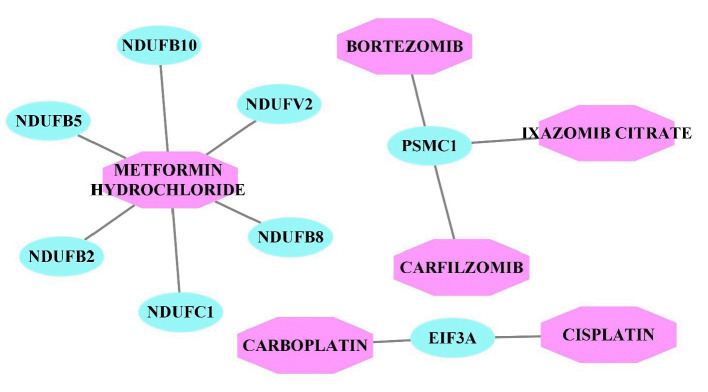
Drug-gene interaction network. Purple octagons represent drugs, while blue ellipse nodes denote genes in the protein-protein interaction module.

The DGIdb was used to identify drugs targeting the genes of the modules. The selected modules’ genes were merged; then, these genes were then imported into the DGIdb, and approved drugs targeting these genes were extracted. After detecting drug-gene interactions for the genes of all modules, the obtained interactions were utilized to construct a drug-gene network. Based on drug-gene interactions, six drugs were detected. However, we found no drug targeting multiple genes in the PPI modules. Finally, Cytoscape (version 3.6) was used to visualize the drug-gene network (Fig. 3), and the drug metformin hydrochloride was found to target more than one gene. Bortezomib, ixazomib citrate, carfilzomib, carboplatin, and cisplatin targeted only a single gene each. The mentioned drugs can be repurposed to treat POAG.

## Discussion

This study was performed to detect more effective drugs for POAG treatment, as well as to better understand POAG pathogenesis. Accordingly, DEGs were obtained between POAG and control samples. A total of 1,350 genes with p < 0.01 and |log fold change| > 0.3 were categorized as DEGs. Biological pathway analysis indicated that the upregulated DEGs were enriched in ECM organization, the immune system, neutrophil degranulation, ECM degradation, the innate immune system, PDGF signaling, and many other processes (Supplementary Table 2). The role of the ECM has been evaluated in outflow homeostasis and its related potential as a target for POAG treatment. Based on this study, the ECM is a promising target for future glaucoma treatment [16]. Identifying ECM molecules is a key challenge, as these must be therapeutically targeted for improving the extracellular environment [17]. Separately, previous studies have indicated that immune system regulation is strongly involved in the fates of glial and retinal ganglion cells that cause glaucomatous optic nerve degeneration. Therefore, an improved understanding of the varied roles of the immune system in any glaucomatous optic nerve degeneration will help advance neuroprotective strategies for glaucoma [18]. Neutrophil penetration and degranulation occur on the ocular surface. Degranulation and neutrophil extracellular trap development (termed NETosis) indicate that autophagy in neutrophils is involved in the pathogenesis of ocular surface diseases. Understanding the role of neutrophils on the ocular surface is necessary [19].

Environmental factors and genetics have also been reported to contribute to the enhanced risk of glaucoma. Evidence now indicates that epigenetics may also play a critical role, offering potential novel therapeutic targets. Furthermore, several pathways are involved in the progression of this disease, such as Rho kinase, transforming growth factor-β, JNK, mitogen-activated protein kinase, PTEN, Bcl-2, brain-derived neurotrophic factor, calcium-calpain signaling, and phosphoinositide 3-kinase/Akt caspase. These pathways result in the upregulation of proapoptotic gene expression, the downregulation of neuroprotective and survival factors, and the generation of fibrosis at the trabecular meshwork, which may block the aqueous humor drainage system. Novel therapeutic agents targeting each component of these pathways have shown initial success in animal models and even human trials, indicating potential for preserving retinal neurons and vision [20]. Additionally, numerous studies have shown the role of the *PDGF* family in ocular disorders involving degeneration of neuronal and vascular retinal cells. This supports the importance of model study and analysis of this gene family and targeting-related cascade in the effort to cure neurodegenerative ocular disorders [21].

Downregulated DEGs were mainly involved in biological pathways including signal transduction, ECM organization, the p53 apoptotic cascade, and toll-like receptor cascades. Importantly, pathways related to various forms of signal transduction, ECM organization, and the immune system were noted among both upregulated and downregulated DEGs. However, for p53, understanding the apoptosis pathway and key genes involved in this process is crucial. For example, p53 may control the expression level of proapoptotic and antiapoptotic genes, with diseases caused by its downregulation of apoptotic processes [22].

However, the results could differ completely based on geographical context. For instance, a cohort study in Iran revealed that a polymorphism of p53 (Arg72Pro) was correlated with POAG, while another study of Japanese patients demonstrated no relation between p53 polymorphisms and the disease; thus, these studies have drawn inconsistent conclusions [23,24].

Toll-like receptor 4 is an active component of the innate immune system that was repeatedly mentioned in the pathway enrichment analysis. Similarly, it is involved in cell death, and its downregulation could impact the cell survival of retinal ganglion cells through decreasing TGF-β2–induced fibrosis [25]. Based on these pathways, more studies are needed to understand the key targets for POAG treatment.

We constructed a PPI network based on DEGs between patients with POAG and control samples, with both sets of samples taken from the trabecular meshwork. Further investigations were based on three modules of proteins that were most correlated with POAG. Generally, the main goal of this study was to discover novel therapeutic candidate drugs for gene regulation in POAG cases. Hence, we constructed a gene-drug network, and we identified several drugs, including metformin hydrochloride (which targets multiple genes); bortezomib, ixazomib citrate, and carfilzomib (which target the *PSMC1* gene); and carboplatin and cisplatin (which target the *EIF3A* gene) for potential repurposing. Clinical and laboratory studies should be conducted to confirm their effectiveness in POAG treatment.

Metformin hydrochloride targets several genes from the *NDUF* family. The role of metformin in glaucoma, especially primary-angle, has been well established. Although a cohort study revealed no positive relationship between this drug and POAG, other research has indicated a strong association between decreasing risk of glaucoma progression and metformin uptake [26]. Another study declared that among all diabetic drugs, only this drug positively impacts sight-threatening diseases, including POAG [27]. Additionally, the mechanism of action of metformin could be varied; it may improve glycemic control, or it may affect other mechanisms such as neurogenesis, inflammatory systems, or longevity pathways. However, all past researchers have studied the role of metformin alone, and no information is available regarding the drug we noted (metformin hydrochloride).

The ubiquitin-proteasome system (UPS) is an adaptive proteolytic system that controls numerous cellular processes, including protein degradation, DNA repair, stress responses, and cell proliferation [28]. Dysfunction of this system is directly linked with the incidence and progression of human cancers and neurodegenerative disorders such as glaucoma [29]. Therefore, drugs that target this system could have positive impacts in controlling the related disease. Three drugs obtained from our data analysis (bortezomib, ixazomib citrate, and carfilzomib) have been mentioned as proteasome inhibitors, and the US Food and Drug Administration has approved bortezomib based on its impact on the UPS [29]. Later, two other drugs (ixazomib and carfilzomib) were also approved for preventing resistance to bortezomib [29].

Moreover, other drugs with proteasome-inhibiting activities have been investigated for the treatment of hematological malignancies and solid tumors [29]. Notably, that study also indicates that one contributor to POAG is the pathological remodeling of trabecular meshwork cells, leading to obstructed outflow of aqueous humor; this is due to the dysfunction of the UPS in enhancing apoptosis in trabecular meshwork cells (the tissue exclusively studied in the present study) [29]. Nevertheless, as with metformin hydrochloride, no specific information is available, particularly regarding the role of ixazomib citrate in POAG.

Although no information is available about the impact of cisplatin in POAG, one study assessed the role of this drug to obtain the relationship between the impact of this drug and specific methylation patterns of individuals. This research indicated that specific mitochondrial DNA patterns are associated with different responses to cisplatin treatment [30].

Lastly, little data are available from the phase I/II clinical trial study of subconjunctival carboplatin injection on retinoblastoma and eye-related disorders. Although they described carboplatin as encouraging, those authors still recommended enucleation when the patient lacks functional vision, such as in cases of large tumors, long-standing retinal detachment, and neovascular glaucoma [31]. In such cases, while surgery is far more efficient, drug treatment is beneficial to prevent metastasis, especially in eye-related cancers.

Overall, since most of the drugs obtained in this study are related to cancer in general or eye-related cancers in particular, their administration in glaucoma has been insufficiently investigated. Therefore, in accordance with our study’s aims, we propose that drugs including metformin hydrochloride, ixazomib citrate, and cisplatin are novel and warrant further clinical and laboratory investigations of their potential in treating glaucoma.

Here, we aimed to compare the trabecular meshwork tissue of healthy patients with that of patients with POAG. We identified 1,350 DEGs, then constructed a PPI network based on significant DEGs (p < 0.01 and |log fold change| > 0.3). Lastly, we reconstructed a drug-gene network for the genes of the PPI modules. However, our study consisted of computational analysis and lacked experimental methods to confirm the findings. In particular, the candidate drugs obtained in this study require *in vitro* and *in vivo* validation of each drug's safety and usefulness. In that case, the drugs proposed in this study could pave the way to understanding life-threatening disorders such as cancer and eye disease.

## Figures and Tables

**Table 1.** List of cluster analysis results by ClusterVis

	PPI module 1	PPI module 2	PPI module 3
Rank	Rank 2	Rank 3	Rank 1
Nodes	16	9	13
Edges	20	20	33
Modularity	4	2.8	8.25

PPI, protein-protein interaction.

## Supplementary Materials

Supplementary Table 1. All 1,350 significant differentially expressed genes (p < 0.01 and |log fold change| > 0.3)

Supplementary Table 2. Upregulated genes based on Reactome pathway enrichment analysis

Supplementary Table 3. Upregulated genes based on molecular function enrichment analysis

Supplementary Table 4. Upregulated genes based on cell component enrichment analysis

Supplementary Table 5. Upregulated genes based on biological process enrichment analysis

Supplementary Table 6. Downregulated genes based on Reactome pathway enrichment analysis

Supplementary Table 7. Downregulated genes based on molecular function enrichment analysis

Supplementary Table 8. Downregulated genes based on cell component enrichment analysis

Supplementary Table 9. Downregulated genes based on biological process enrichment analysis

Supplementary Table 10. Reactome pathway results for protein-protein interaction module 1

Supplementary Table 11. Reactome pathway results for protein-protein interaction module 2

Supplementary Table 12. Reactome pathway results for protein-protein interaction module 3

Supplementary Table 13. Biological process results for protein-protein interaction module 1

Supplementary Table 14. Cell component results for protein-protein interaction module 1

Supplementary Table 15. Molecular function results for protein-protein interaction module 1

Supplementary Table 16. Biological process results for protein-protein interaction module 2

Supplementary Table 17. Cell component results for protein-protein interaction module 2

Supplementary Table 18. Molecular function results for protein-protein interaction module 2

Supplementary Table 19. Biological process results for protein-protein interaction module 3

Supplementary Table 20. Cell component results for protein-protein interaction module 3

Supplementary Table 21. Molecular function results for protein-protein interaction module 3

## References

[1] Suo L, Dai W, Qin X, Li G, Zhang D, Cheng T (2022). Screening of primary open-angle glaucoma diagnostic markers based on immune-related genes and immune infiltration. BMC Genom Data.

[2] Imperato JS, Zou KH, Li JZ, Hassan TA (2022). Clinical practice management of primary open-angle glaucoma in the United States: an analysis of real-world evidence. Patient Prefer Adherence.

[3] Hosseini SS, Abedi Z, Maghsoudloo M, Sheikh Beig Goharrizi MA, Shojaei A (2019). Investigation of genes associated with primary open-angle glaucoma (POAG) using expression profile analysis. J Ophthalmol Optom Sci.

[4] Weinreb RN, Leung CK, Crowston JG, Medeiros FA, Friedman DS, Wiggs JL (2016). Primary open-angle glaucoma. Nat Rev Dis Primers.

[5] Gharahkhani P, Jorgenson E, Hysi P, Khawaja AP, Pendergrass S, Han X (2021). Genome-wide meta-analysis identifies 127 open-angle glaucoma loci with consistent effect across ancestries. Nat Commun.

[6] Storgaard L, Tran TL, Freiberg JC, Hauser AS, Kolko M (2021). Glaucoma clinical research: trends in treatment strategies and drug development. Front Med (Lausanne).

[7] Al Owaifeer AM, Al Taisan AA (2018). The role of diet in glaucoma: a review of the current evidence. Ophthalmol Ther.

[8] Schmidl D, Schmetterer L, Garhofer G, Popa-Cherecheanu A (2015). Pharmacotherapy of glaucoma. J Ocul Pharmacol Ther.

[9] Abedi Z, MotieGhader H, Maghsoudloo M, Sheikh Beig Goharrizi MA, Shojaei A, Masoudi-Nejad A (2019). Novel potential drugs for therapy of age-related molecular degeneration using protein-protein interaction network (PPI) analysis. J Ophthalmic Optom Sci.

[10] Liu Y, Allingham RR, Qin X, Layfield D, Dellinger AE, Gibson J (2013). Gene expression profile in human trabecular meshwork from patients with primary open-angle glaucoma. Invest Ophthalmol Vis Sci.

[11] Ritchie ME, Phipson B, Wu D, Hu Y, Law CW, Shi W (2015). limma powers differential expression analyses for RNA-sequencing and microarray studies. Nucleic Acids Res.

[12] Chen SJ, Liao DL, Chen CH, Wang TY, Chen KC (2019). Construction and analysis of protein-protein interaction network of heroin use disorder. Sci Rep.

[13] Sherman BT, Hao M, Qiu J, Jiao X, Baseler MW, Lane HC (2022). DAVID: a web server for functional enrichment analysis and functional annotation of gene lists (2021 update). Nucleic Acids Res.

[15] Freshour SL, Kiwala S, Cotto KC, Coffman AC, McMichael JF, Song JJ (2021). Integration of the Drug-Gene Interaction Database (DGIdb 4.0) with open crowdsource efforts. Nucleic Acids Res.

[16] O'Callaghan J, Cassidy PS, Humphries P (2017). Open-angle glaucoma: therapeutically targeting the extracellular matrix of the conventional outflow pathway. Expert Opin Ther Targets.

[17] Keller KE, Peters DM (2022). Pathogenesis of glaucoma: extracellular matrix dysfunction in the trabecular meshwork. A review. Clin Exp Ophthalmol.

[18] Tezel G, Wax MB (2004). The immune system and glaucoma. Curr Opin Ophthalmol.

[19] Mun Y, Hwang JS, Shin YJ (2021). Role of neutrophils on the ocular surface. Int J Mol Sci.

[20] Gauthier AC, Liu J (2017). Epigenetics and signaling pathways in glaucoma. Biomed Res Int.

[21] Kumar A, Li X (2018). PDGF-C and PDGF-D in ocular diseases. Mol Aspects Med.

[22] Nickells RW (1999). Apoptosis of retinal ganglion cells in glaucoma: an update of the molecular pathways involved in cell death. Surv Ophthalmol.

[23] Mabuchi F, Sakurada Y, Kashiwagi K, Yamagata Z, Iijima H, Tsukahara S (2009). Lack of association between p53 gene polymorphisms and primary open angle glaucoma in the Japanese population. Mol Vis.

[24] Dimasi DP, Hewitt AW, Green CM, Mackey DA, Craig JE (2005). Lack of association of p53 polymorphisms and haplotypes in high and normal tension open angle glaucoma. J Med Genet.

[25] Poyomtip T (2019). Roles of toll-like receptor 4 for cellular pathogenesis in primary open-angle glaucoma: a potential therapeutic strategy. J Microbiol Immunol Infect.

[26] George R, Asokan R, Vijaya L (2021). Association of metformin use among diabetics and the incidence of primary open-angle glaucoma: the Chennai Eye Disease Incidence Study. Indian J Ophthalmol.

[27] Lin HC, Stein JD, Nan B, Childers D, Newman-Casey PA, Thompson DA (2015). Association of geroprotective effects of metformin and risk of open-angle glaucoma in persons with diabetes mellitus. JAMA Ophthalmol.

[28] Park J, Cho J, Song EJ (2020). Ubiquitin-proteasome system (UPS) as a target for anticancer treatment. Arch Pharm Res.

[29] Tundo GR, Sbardella D, Santoro AM, Coletta A, Oddone F, Grasso G (2020). The proteasome as a druggable target with multiple therapeutic potentialities: cutting and non-cutting edges. Pharmacol Ther.

[30] Abedi S, Yung G, Atilano SR, Thaker K, Chang S, Chwa M (2020). Differential effects of cisplatin on cybrid cells with varying mitochondrial DNA haplogroups. PeerJ.

[31] De Potter P (2002). Current treatment of retinoblastoma. Curr Opin Ophthalmol.

