# Supplementary material for: Novel potential drugs for the treatment of primary open-angle glaucoma using protein-protein interaction network analysis
Source: Genomics Inform. 2023 Mar 31;21(1):e6. doi: 10.5808/gi.22070 (PMC10085733; doi:10.5808/gi.22070)
Supplement: Supplementary Table 1. — All 1,350 significant differentially expressed genes (p < 0.01 and |log fold change| > 0.3) [file gi-22070-Supplementary-Table-1.pdf]

Supplementary Table 1. All 1,350 significant differentially expressed genes ($p < 0.01$ and |log fold change| > 0.3)

Gene symbol	p-value	LogFC
KRT19	4.15E-10	-4.34
CALML3	5.18E-10	-3.74
KRT15	1.18E-07	-3.39
KRT13	4.84E-09	-3.37
PAX6	1.17E-08	-2.92
SLPI	3.05E-09	-2.9
LCN2	1.77E-08	-2.88
S100A9	5.74E-07	-2.78
S100A8	7.45E-05	-2.66
C19orf33	3.02E-08	-2.63
TGM1	1.11E-07	-2.52
KRT14	2.27E-06	-2.47
KRT5	4.98E-07	-2.45
TNNT3	6.81E-09	-2.4
HP	2.52E-07	-2.34
BCAS1	1.13E-06	-2.28
PROM2	4.87E-07	-2.26
CEBPD	1.36E-11	-2.18
FAM3D	2.76E-07	-2.18
MPZL2	4.47E-08	-2.17
PRSS8	2.09E-08	-2.1
GCNT3	2.05E-08	-2.06
CEACAM6	3.56E-06	-1.87
PKP3	4.07E-06	-1.82
CHP2	2.12E-07	-1.8
MMP3	0.000102	-1.79
HOPX	5.36E-07	-1.74
MSLN	5.46E-08	-1.73
S100A2	0.000136	-1.72
MUC21	6.33E-08	-1.71
ZFP36	3.24E-07	-1.68
CSRNP1	7.43E-09	-1.67
ATP10B	3.58E-05	-1.62
CSRP2	0.00012	-1.6
C10orf10	8.80E-07	-1.59
S100A14	4.58E-06	-1.58
BNIPL	1.88E-06	-1.57
HCAR3	3.03E-06	-1.57
LAMB3	1.88E-06	-1.57
ESRP2	1.41E-05	-1.55
IL1RN	1.35E-05	-1.55
KLK11	9.00E-07	-1.55

UPK1B	3.93E-06	-1.5
CAPNS2	3.77E-06	-1.48
ARL4D	2.57E-06	-1.47
MASP1	5.43E-10	-1.46
MYH11	0.00949	-1.46
FAM3B	2.68E-06	-1.44
MUC20	1.50E-06	-1.44
FAM83A	2.43E-05	-1.42
TSPAN1	1.39E-05	-1.42
DEFB1	2.16E-05	-1.41
LGALS7	0.000358	-1.41
MAOA	4.99E-08	-1.41
MT1A	1.87E-06	-1.41
RASD1	8.47E-06	-1.4
SAA2	0.00184	-1.4
SERPINA3	7.79E-05	-1.4
ABCA12	3.65E-06	-1.39
AP1M2	1.54E-06	-1.37
CFD	2.73E-08	-1.37
MIDN	5.32E-06	-1.37
SCNN1A	1.70E-06	-1.37
HILPDA	0.000165	-1.36
CSF3	0.00119	-1.34
TMC4	0.000235	-1.34
DUSP1	2.03E-06	-1.33
TNXB	9.43E-08	-1.33
AQP3	0.000207	-1.3
CMIP	2.78E-08	-1.3
MYH14	1.56E-08	-1.3
BIRC3	0.000216	-1.29
KLC3	2.65E-05	-1.29
MT2A	1.31E-06	-1.29
TYRP1	0.000812	-1.29
DTX2	5.84E-07	-1.28
FAM83H	1.26E-05	-1.28
BHLHE40	3.06E-05	-1.27
NAMPT	8.79E-06	-1.26
PLVAP	2.81E-05	-1.26
ARHGEF16	6.13E-05	-1.25
MTNR1A	2.97E-06	-1.25
BCL11A	0.000144	-1.24
ERICH5	1.26E-06	-1.23
OSR2	2.36E-09	-1.22
CITED4	1.04E-07	-1.19
DDIT4	7.36E-05	-1.19

FABP5	9.58E-05	-1.19
IL20RB	1.14E-05	-1.19
SLN	0.00515	-1.19
ELF3	4.20E-05	-1.17
GPX3	0.00105	-1.17
LGI4	0.000803	-1.17
RASSF7	0.000115	-1.17
AVPI1	3.62E-05	-1.16
TMEM79	0.000746	-1.16
FOSL2	1.74E-06	-1.15
ZNF750	3.28E-07	-1.14
EZR	1.28E-05	-1.13
DUSP4	6.02E-07	-1.12
SCGB2A1	0.0012	-1.12
AQP5	1.07E-06	-1.1
TOX2	0.00027	-1.1
ADH1B	3.02E-05	-1.09
CHI3L2	0.00127	-1.09
MT1M	0.00022	-1.09
TMEM30B	4.27E-06	-1.09
ZG16B	0.000414	-1.09
CEACAM7	0.000163	-1.08
DCT	1.95E-06	-1.08
MOCOS	3.79E-06	-1.08
S100A11	0.00014	-1.08
S100A12	0.000139	-1.08
CDC14B	2.06E-09	-1.07
DUSP3	1.58E-05	-1.07
CEACAM5	0.000189	-1.06
HES4	1.16E-06	-1.06
MMP1	0.00542	-1.06
SLC2A3	1.59E-05	-1.06
RBPMS2	0.00957	-1.05
SLC1A5	5.93E-08	-1.05
CLDN7	1.26E-05	-1.04
DUSP5	3.10E-06	-1.04
GADD45B	1.77E-05	-1.04
FLOT2	1.79E-05	-1.03
KRT17	0.00201	-1.03
PTP4A3	0.00589	-1.02
GDF15	2.63E-05	-1
SLC6A14	0.000119	-0.991
HIST2H2BE	4.96E-05	-0.985
CROT	0.000397	-0.982
TNFRSF21	0.00213	-0.975

TRIM29	7.62E-06	-0.973
BZW2	1.86E-05	-0.967
THOC3	3.07E-06	-0.967
TWIST2	0.000581	-0.965
CD55	0.00155	-0.953
RGMA	5.17E-09	-0.949
CXCL17	2.01E-06	-0.946
NAPRT	2.33E-05	-0.937
MEIS1	6.93E-06	-0.935
BCL6	6.42E-06	-0.932
RAB25	2.52E-05	-0.929
TUBA4A	1.19E-05	-0.929
GNA15	0.000131	-0.927
RASAL1	2.63E-05	-0.924
SFN	0.000842	-0.924
PPDPF	6.61E-05	-0.911
TMPRSS11D	0.00294	-0.911
RHOU	2.03E-08	-0.902
VWF	0.00256	-0.9
MT1X	0.00169	-0.896
RBBP8NL	0.000273	-0.891
FGFBP1	0.00156	-0.885
PRRG2	0.000124	-0.878
C1orf106	5.62E-05	-0.872
A4GALT	7.10E-07	-0.87
KCNJ13	0.00102	-0.868
DPT	0.00185	-0.863
FAM60A	6.35E-09	-0.863
PXN	9.76E-07	-0.856
ARL4A	0.00271	-0.854
HIST1H2BK	6.23E-05	-0.853
GJB5	4.76E-05	-0.852
TYMP	0.00118	-0.851
KRT18	0.00482	-0.85
NFKBIA	3.67E-06	-0.85
RRAGD	5.98E-05	-0.85
C3	0.00139	-0.847
EFNA1	2.29E-07	-0.843
MLANA	0.0025	-0.834
TPSAB1	0.00895	-0.825
CYP4B1	0.000143	-0.821
KLF4	7.26E-05	-0.82
FERMT1	0.00251	-0.817
MFN2	3.08E-05	-0.812
CDH19	0.00112	-0.811

POF1B	2.64E-05	-0.811
SH3BP1	7.76E-06	-0.811
MRPL12	0.00014	-0.809
LSR	0.000174	-0.804
C11orf58	0.000626	-0.803
BTG1	0.000297	-0.801
VWA1	3.30E-05	-0.801
SERPINB5	0.000791	-0.795
FOXN3	2.06E-05	-0.794
PHC2	6.03E-05	-0.79
ZNF296	0.000274	-0.79
PODN	0.0026	-0.789
CXCL1	0.00301	-0.778
UBE2V1	5.09E-09	-0.778
BCO1	3.10E-05	-0.777
KLF9	0.0021	-0.773
PIGR	6.47E-05	-0.772
HIST2H2AC	9.55E-07	-0.769
CAPN1	4.77E-05	-0.768
MARK2	7.98E-06	-0.767
LMO4	1.43E-06	-0.764
KLHL8	1.38E-05	-0.755
VAV3	8.22E-06	-0.751
MECOM	6.16E-05	-0.75
PDCD4	3.13E-05	-0.749
SERPINB1	0.00791	-0.749
UGCG	0.000918	-0.749
FAM83F	4.73E-06	-0.741
EIF4G3	0.00114	-0.74
ZRANB1	0.0077	-0.74
RNF122	2.94E-09	-0.736
CGN	0.000569	-0.735
CKMT1B	0.00344	-0.735
MAT2A	0.0035	-0.725
SORD	0.000183	-0.725
SULT2B1	0.000195	-0.723
FABP4	0.00068	-0.722
LAMA5	0.000258	-0.722
ADM	2.04E-06	-0.721
MRPL28	4.58E-05	-0.72
CLDN1	0.000194	-0.719
PSME4	1.46E-05	-0.718
S100P	0.00352	-0.718
GPN2	6.06E-06	-0.717
F3	0.000209	-0.716

SYT17	0.00114	-0.716
TRIB1	0.00156	-0.713
YBX3	0.00474	-0.71
IMPA2	0.00127	-0.707
RBM47	0.000388	-0.705
FLNC	0.00775	-0.703
VPS37B	0.00963	-0.7
CDCA7L	5.12E-05	-0.699
AGPAT2	2.92E-07	-0.698
HOOK1	0.000125	-0.697
TFF1	0.00264	-0.694
ADAMTS4	0.00273	-0.693
EYA2	4.09E-05	-0.693
PDLIM1	4.24E-05	-0.69
MALL	0.000146	-0.686
DTNB	0.00182	-0.685
POU2F1	0.00267	-0.684
CASZ1	0.00124	-0.683
EXT1	0.00799	-0.682
SPRY2	0.000285	-0.678
GPIHBP1	0.009	-0.677
FCN3	9.97E-05	-0.675
RELN	0.000286	-0.674
LIMK2	0.000604	-0.673
TAPBP	5.14E-05	-0.673
CDKN2B	0.00271	-0.672
ANKHD1	1.69E-05	-0.666
HIST1H1C	0.00067	-0.665
BSPRY	0.00313	-0.664
NDRG1	0.000112	-0.664
CCDC120	0.000203	-0.658
PSMC1	6.43E-07	-0.658
AUTS2	5.69E-05	-0.656
STAP2	0.00441	-0.655
GLTSCR2	1.80E-06	-0.654
ADIRF	0.00192	-0.653
RAB38	0.000144	-0.652
HTRA3	0.00031	-0.641
CLEC2B	0.00819	-0.638
KRT4	0.00124	-0.637
RBPMS	0.00493	-0.637
CLIC6	0.00067	-0.636
EVPL	0.000457	-0.636
SDC4	4.66E-06	-0.636
GJB2	0.00148	-0.634

PLPP2	0.000338	-0.634
TRIB3	0.000317	-0.634
ALKBH5	0.00593	-0.633
MTFP1	0.000307	-0.633
ASS1	0.000139	-0.632
TNFRSF1A	2.97E-06	-0.632
RAPGEFL1	3.55E-05	-0.631
C6orf47	2.34E-05	-0.63
PPP2CA	3.88E-06	-0.629
CNKSR1	0.000252	-0.628
LRG1	0.00202	-0.628
MAMDC2	0.000102	-0.628
PTGES	0.000717	-0.628
RPS5	4.70E-05	-0.628
GPX2	0.00328	-0.625
LYPD2	0.00291	-0.624
TRIM63	0.00622	-0.618
RARA	7.01E-05	-0.616
ZNF207	2.22E-05	-0.616
GLDN	6.58E-05	-0.613
TACC2	0.00176	-0.605
BMP1	0.000745	-0.604
ACKR1	1.09E-05	-0.601
NOP16	0.00317	-0.598
RAC1	6.74E-05	-0.598
DST	2.87E-05	-0.596
WAC	3.62E-08	-0.596
SH3BGRL2	3.11E-05	-0.595
GFM1	0.00022	-0.594
PCDH1	8.36E-05	-0.592
FOXQ1	6.39E-06	-0.59
PRRX2	0.000132	-0.589
EPS8L2	0.00774	-0.587
KDM5B	1.82E-06	-0.586
FBLN2	0.000284	-0.585
TALDO1	0.000867	-0.585
DIAPH2	1.38E-05	-0.583
VASP	6.04E-05	-0.583
MKL1	6.01E-05	-0.58
NDUFV2	1.93E-05	-0.578
CCL13	0.000292	-0.576
SMAGP	0.00115	-0.576
SQSTM1	4.14E-06	-0.575
ADK	1.69E-05	-0.573
FMO1	0.00111	-0.573

RPF2	0.00121	-0.572
ABCA1	5.37E-06	-0.57
CAST	0.000334	-0.57
SH2D4A	0.00023	-0.569
SHPK	0.000124	-0.567
STK24	1.12E-05	-0.567
ZFAND5	0.00116	-0.566
BACE2	0.000209	-0.565
CYP1B1	0.00976	-0.564
COBLL1	0.00759	-0.563
LRRC26	0.00254	-0.562
UBIAD1	0.000122	-0.562
ACTN1	0.00262	-0.56
NTNG1	0.0032	-0.56
LGR4	4.28E-05	-0.558
SLC25A10	0.000702	-0.555
FAM110A	0.00116	-0.554
C11orf80	0.00268	-0.553
CDS1	0.00137	-0.551
IKBKB	2.00E-04	-0.551
AREG	0.00317	-0.548
KCNJ2	8.56E-05	-0.546
NOTCH2NL	0.000901	-0.546
MKRN1	4.36E-06	-0.545
DAAM1	0.00498	-0.543
IGSF9	6.27E-05	-0.539
TYW3	0.000973	-0.539
GNAI3	0.000371	-0.538
PLCH2	0.00145	-0.538
STK3	0.000331	-0.537
UAP1	0.00332	-0.537
ZCCHC6	3.59E-06	-0.537
DHRS3	0.00415	-0.536
EIF2A	0.00115	-0.534
LILRA5	0.00705	-0.533
DLC1	0.00556	-0.532
TMEM189	0.000385	-0.53
ADH1A	0.00371	-0.529
RNF149	0.000118	-0.525
IFT57	0.0057	-0.523
STARD8	0.00112	-0.519
PERP	0.00134	-0.518
BRWD3	0.00054	-0.517
PPA2	0.00733	-0.517
ZNF581	0.000125	-0.517

KHSRP	0.00032	-0.516
PPP1R13B	0.0043	-0.516
PPP1R15A	0.00166	-0.516
VGLL4	0.00256	-0.514
CTDP1	3.78E-06	-0.512
FGFBP2	0.00681	-0.512
RBPJ	0.0011	-0.511
VGLL1	0.0017	-0.511
ZBTB16	0.00818	-0.511
TBC1D22A	0.00143	-0.51
HIST1H2BE	8.04E-06	-0.508
GSR	0.00128	-0.506
MON1B	0.000669	-0.506
ALDH3B2	0.00276	-0.505
CEACAM1	0.00869	-0.505
TEAD3	6.38E-07	-0.505
KAT8	0.000141	-0.504
SH3YL1	0.00298	-0.504
TMEM55B	0.00727	-0.504
LLGL2	0.00174	-0.503
TMC5	0.00108	-0.503
TRIM16	0.00975	-0.503
CSNK1E	0.00738	-0.502
KLK12	0.00129	-0.502
ALK	0.00122	-0.501
CARHSP1	0.00936	-0.501
SASH1	0.000245	-0.499
OTX1	6.00E-05	-0.498
CAMLG	0.000822	-0.497
HIF1A	0.00666	-0.497
RSPH14	0.00154	-0.496
IL1R1	0.000105	-0.491
DOHH	0.00228	-0.49
TFPI	0.00013	-0.49
MAPK13	0.00327	-0.489
PPP6C	0.000815	-0.489
CC2D1A	0.00448	-0.486
MKLN1	0.000857	-0.486
PCSK5	0.00308	-0.486
STK38	7.18E-05	-0.486
PPP4R1	0.00712	-0.485
WWP1	0.00118	-0.485
STXBP5	9.63E-07	-0.483
MTPAP	0.00665	-0.482
DERA	0.000351	-0.481

SLCO2A1	0.00473	-0.481
DNAJC7	0.00353	-0.48
DBT	0.00833	-0.479
OTUB1	0.00325	-0.479
ABCG1	0.0021	-0.478
CNFN	0.00534	-0.478
FPGS	0.00439	-0.477
MED4	0.000695	-0.477
TYSND1	0.00523	-0.473
PPP4R2	0.00922	-0.471
FLOT1	0.00258	-0.466
FBXO34	2.27E-05	-0.465
POLR2J	0.00127	-0.464
BBOX1	0.00587	-0.462
IQGAP1	0.000819	-0.462
SOX15	0.000929	-0.461
DRAP1	0.0036	-0.456
HECA	0.00292	-0.454
RIPK4	0.00524	-0.454
SELP	0.00045	-0.451
SH3RF2	0.00615	-0.451
ZSWIM8	0.003	-0.451
PUM3	0.000787	-0.447
ZYX	0.00222	-0.446
MYO5A	0.000147	-0.445
DIMT1	0.00158	-0.444
MXI1	8.65E-05	-0.444
UNC93A	0.000733	-0.444
EIF3A	0.00368	-0.443
ZNF777	0.00947	-0.443
SSU72	3.52E-05	-0.442
ZNF395	0.000779	-0.442
ARL6IP4	0.00176	-0.441
PLIN2	0.000907	-0.441
GTF2E2	0.000112	-0.439
USP32	0.000108	-0.439
AKR1A1	0.00278	-0.437
SCO1	0.000117	-0.437
PPP2R1A	0.00571	-0.436
ETHE1	0.00626	-0.435
ABRACL	0.00948	-0.434
UBQLN1	0.00127	-0.434
PICK1	0.000281	-0.431
XRCC5	0.000675	-0.431
LAMC2	0.000553	-0.43

PLEKHN1	0.00107	-0.43
BRAP	4.22E-05	-0.429
C12orf54	0.00069	-0.429
LRP5L	7.03E-05	-0.428
ZDHHC5	0.00127	-0.428
TNFAIP6	0.000139	-0.427
EIF4EBP1	0.000239	-0.426
PM20D2	0.00746	-0.426
RNASEH1	0.00214	-0.426
XAB2	0.00466	-0.426
AZGP1	0.000241	-0.423
SLC7A6	0.00265	-0.42
MED25	0.000145	-0.419
RASSF6	0.0037	-0.418
RPL32	2.33E-06	-0.418
TCF20	0.000948	-0.415
ADCY9	0.00956	-0.414
ABCB7	0.00966	-0.413
RAB18	0.00947	-0.413
UBE2D3	8.85E-05	-0.413
KPNA6	0.000617	-0.412
AKAP8	9.22E-05	-0.411
RABGGTA	0.000672	-0.411
ARID2	0.000702	-0.41
VPS36	0.00098	-0.41
MORC2	0.00041	-0.409
GIPC1	0.00348	-0.408
KRT24	0.0057	-0.407
PIM1	0.000507	-0.407
BMP7	0.000713	-0.405
CTSD	0.0082	-0.405
NDE1	0.00298	-0.405
NUDT21	0.00968	-0.405
SPTBN2	0.00567	-0.403
CHSY1	0.00214	-0.402
RSL24D1	0.00243	-0.401
THAP4	0.00221	-0.401
MAP3K6	0.00142	-0.4
PPM1G	0.000428	-0.4
ADAMTS9	0.00219	-0.398
HIST1H4J	0.00868	-0.398
TSPAN9	0.00141	-0.398
CBX3	0.00112	-0.395
ACE2	0.00123	-0.394
ERO1A	0.0028	-0.394

HDAC11	0.00774	-0.393
PGRMC2	0.000198	-0.391
EFHD2	0.00237	-0.389
EIF2B4	5.70E-05	-0.389
SHMT2	0.00283	-0.388
CD44	0.00171	-0.386
DMD	0.00238	-0.386
MYPOP	0.00726	-0.386
WBP11	2.36E-05	-0.385
FBXO11	0.000513	-0.384
MED16	7.31E-05	-0.383
CCDC12	0.000112	-0.382
TBCD	0.00638	-0.382
EDARADD	0.00189	-0.381
SLX1B	0.00965	-0.381
TOB1	0.00657	-0.381
CHCHD6	0.000349	-0.378
FOXJ2	0.00714	-0.378
IRF1	0.000305	-0.378
RNF181	0.00219	-0.377
TMEM11	0.000284	-0.377
RUNX3	0.00703	-0.374
STK39	0.00438	-0.374
GTF3C5	0.00771	-0.373
TP53INP1	0.00609	-0.372
BCORL1	0.000852	-0.371
KDM2A	0.000985	-0.371
CDCP1	0.00228	-0.369
ARHGAP10	0.00512	-0.368
VPS37C	0.00283	-0.366
DUOXA1	0.000816	-0.365
PAPOLA	0.00435	-0.365
RNF43	0.00467	-0.365
CALML4	0.000389	-0.364
UBE3A	0.0037	-0.364
MAP3K1	0.00625	-0.363
MARVELD3	0.00478	-0.362
ACVR1B	0.00329	-0.36
FLT4	0.000648	-0.36
ZNF438	0.00429	-0.359
SLC25A28	0.00066	-0.358
HIST1H2BH	0.000429	-0.357
MUC1	0.00211	-0.357
AMBRA1	8.18E-05	-0.353
HDAC8	0.000847	-0.353

SLITRK6	0.00106	-0.353
HRAS	0.00539	-0.352
GAK	0.00361	-0.35
CEP57	0.00658	-0.348
DHX37	0.00998	-0.347
FAM120B	0.00928	-0.346
TNNI2	0.00231	-0.346
ZNF622	0.00224	-0.346
PSME1	0.00441	-0.345
SGK223	0.000824	-0.345
COL11A1	0.00131	-0.344
SDCBP2	0.00478	-0.344
SPHK1	0.00341	-0.344
ALKBH6	0.00215	-0.343
PIH1D1	0.00554	-0.342
TMUB2	0.00322	-0.342
PIGA	0.0065	-0.341
RAD23A	0.00621	-0.341
CYP4F12	0.00445	-0.34
JMY	0.00659	-0.34
TBL3	0.00879	-0.34
TOM1	0.000654	-0.34
CDC42	0.00722	-0.337
RAN	0.00578	-0.337
SMC5	0.000462	-0.335
ALPK1	0.00496	-0.333
BCL3	0.000195	-0.333
WDR70	0.000181	-0.333
JAG1	0.00549	-0.332
FPR1	0.000946	-0.331
HIST1H2AE	0.00133	-0.33
RND3	0.00986	-0.329
RALBP1	0.00191	-0.328
MAPKAP1	0.00336	-0.327
SPINT1	0.00571	-0.327
CLUH	0.00198	-0.326
EDN2	0.0069	-0.326
TMC6	0.00141	-0.325
USP7	0.000518	-0.325
CSTF2	0.00179	-0.323
KLHL25	0.00866	-0.322
C11orf84	0.00155	-0.321
INPP4B	0.00127	-0.321
PSMB3	0.00971	-0.32
PPL	0.00144	-0.319

ATP6V1B1	0.00161	-0.318
PTP4A1	0.00461	-0.317
TM9SF3	0.00668	-0.316
40057	0.00352	-0.313
FABP6	0.00618	-0.313
SMAD3	0.00719	-0.313
RORA	0.00235	-0.311
RAB2A	0.000827	-0.31
GATA3	0.00183	-0.309
NUBP2	0.00474	-0.309
OBSCN	0.00437	-0.309
MYO10	0.00609	-0.308
PHF23	0.00592	-0.307
COL17A1	0.00625	-0.305
NOTCH1	0.00578	-0.305
BIK	0.00075	-0.303
DLK2	0.000734	-0.303
TTC39A	0.000364	-0.303
L2HGDH	0.00312	-0.302
MYB	0.000964	-0.302
RHPN2	0.00606	-0.302
FUT6	0.00163	0.301
RABEPK	0.0041	0.301
CCDC28B	0.00281	0.302
PAGR1	0.00155	0.302
SLC25A45	0.00864	0.302
SPCS2	0.00808	0.302
ARSG	1.15E-05	0.303
CSGALNACT1	0.00029	0.303
CYB561	0.000509	0.303
FBXO9	9.11E-07	0.303
METRN	0.00185	0.303
PPARGC1B	0.000233	0.303
PTPRN2	0.00344	0.303
SLC22A4	0.000298	0.303
DOCK6	0.00102	0.304
LPCAT1	0.00157	0.304
SLC30A3	0.000951	0.304
GALC	0.000647	0.305
CCDC92	0.00587	0.306
MPP1	0.00224	0.306
PDCD2	0.0037	0.306
PKD1	0.00907	0.306
ZNF177	0.0033	0.306
APMAP	0.00356	0.307

OGDHL	3.55E-05	0.307
C2orf47	0.0082	0.308
FAM181B	0.00175	0.308
PDIA6	0.000132	0.308
L3MBTL2	0.00389	0.309
SC5D	0.000394	0.309
ZFHX4	0.00608	0.309
CA5B	0.00616	0.31
HOMEZ	0.00133	0.31
IGSF21	1.02E-05	0.31
NLGN2	0.00471	0.31
TBC1D14	0.00759	0.31
TMED4	0.000723	0.31
LSS	2.39E-05	0.311
MBNL2	0.00758	0.311
PTDSS1	0.000632	0.311
SFTPD	0.00354	0.311
TMEM17	0.00842	0.312
VIPAS39	0.00209	0.312
MATK	0.000445	0.313
SLC17A9	0.00121	0.313
ACTR10	0.00766	0.314
MRPL57	0.000304	0.314
PIGT	0.0059	0.314
LCAT	0.00376	0.316
METTL21A	0.00652	0.316
SNAP47	0.00187	0.317
WDR13	0.00387	0.317
ANXA4	0.000266	0.319
DARS	0.00627	0.319
SMIM11A	0.000204	0.319
STK32B	0.000741	0.319
TNFSF13B	0.00205	0.319
ZC3HC1	0.00073	0.319
AP3D1	0.0022	0.32
GMPR2	0.00241	0.32
YTHDF1	0.00434	0.32
RNF34	0.00409	0.321
FAM133A	0.00164	0.322
GABARAP	0.0082	0.322
SCG5	0.0041	0.322
PDLIM5	0.00091	0.323
ZNF440	0.00542	0.323
ANKFY1	0.00162	0.324
KANSL2	0.00275	0.324

ANO6	0.00312	0.325
RAP2A	0.000546	0.326
SNX19	0.00635	0.326
ST3GAL5	0.00211	0.326
FAM188A	0.00204	0.327
ZNF430	0.00118	0.327
MAGEB10	0.000805	0.328
ZNF213	0.000121	0.328
MSANTD4	7.61E-05	0.329
SLC22A17	0.00338	0.329
NFAT5	0.000736	0.33
VASH1	3.47E-07	0.33
ZNF264	0.00108	0.33
CD320	0.000192	0.331
FOXJ1	0.00234	0.331
PSMG1	0.00541	0.331
UBP1	0.000915	0.331
HNRNPDL	4.06E-05	0.332
XKR8	0.00372	0.334
NAP1L1	0.00334	0.335
POFUT1	0.000866	0.335
TTF2	0.0013	0.336
RGS7BP	0.000264	0.337
IL18	0.0073	0.338
CHMP5	0.00745	0.339
IFT52	9.31E-05	0.339
SDF4	0.00327	0.34
CCDC24	1.18E-05	0.341
PCSK1N	0.00825	0.341
SLC15A4	0.00357	0.341
ABCA3	1.11E-05	0.342
ALDH4A1	0.000351	0.342
KHDRBS3	0.00575	0.342
GORASP2	0.0028	0.343
ITGAM	1.74E-05	0.343
LRRC27	0.00547	0.343
POFUT2	0.00165	0.343
SLC15A3	0.00505	0.344
ST13	0.00088	0.345
TRIM45	0.000392	0.345
PPBP	4.04E-05	0.346
COPS4	0.000134	0.347
GAA	0.00788	0.347
VSIG4	0.00277	0.347
RFTN2	0.0066	0.348

TERF1	0.00431	0.348
ACAT2	0.00033	0.349
MAP3K12	0.00514	0.349
MYL5	0.00956	0.349
C3orf18	0.00226	0.351
CRIP2	0.00147	0.351
FAM8A1	0.00566	0.351
MPV17	0.0018	0.351
TMEM190	0.00745	0.351
RAI14	0.00476	0.353
TTC17	0.00665	0.353
HLA-DRA	3.90E-05	0.354
MRPL18	0.00299	0.354
CYB5D2	0.000122	0.355
KIDINS220	0.00245	0.355
SSBP4	0.00213	0.355
REEP2	0.000991	0.356
VCL	0.00527	0.356
LMCD1	0.00232	0.357
POMGNT1	0.000176	0.357
RMDN3	0.000261	0.357
CD48	0.000217	0.358
DOCK2	0.000175	0.359
GPC5	0.00908	0.359
PGRMC1	0.000244	0.359
DNAJC10	0.00334	0.361
FBLN7	3.39E-05	0.361
FIBP	0.00218	0.361
PAICS	0.00764	0.361
POGZ	0.00729	0.361
LAMP5	0.000114	0.363
RXRA	0.0044	0.363
LOXL1	0.00124	0.364
WSB1	0.00329	0.364
ZNF546	0.000276	0.364
CAPN2	0.000885	0.367
GLT8D2	6.01E-05	0.367
SGK3	0.000984	0.367
DCTN6	0.000119	0.368
RGN	0.000339	0.369
CRELD1	0.00886	0.37
TIMMDC1	0.00824	0.37
CCL8	0.00101	0.371
CD52	0.00183	0.371
SERPINI1	3.77E-06	0.371

THYN1	0.00978	0.371
LMBRD1	0.000119	0.373
THRA	0.00509	0.373
ETNPPL	0.00151	0.374
CYHR1	0.0066	0.375
GLCE	0.00958	0.375
IL34	0.00813	0.375
MPC1	0.00497	0.375
SLFN12	3.47E-05	0.375
TMEM179B	0.00755	0.376
ZNF160	0.00439	0.377
TMEM204	0.000138	0.378
LGALS3BP	1.32E-06	0.379
SMG1	0.000155	0.379
CLEC4A	0.00339	0.38
PCYOX1	0.00147	0.38
PYCR2	0.00281	0.38
SULF1	2.06E-06	0.38
TRIM9	0.000259	0.38
AKIP1	0.000927	0.381
NTHL1	0.00253	0.381
RERGL	0.00788	0.381
TEK	0.000116	0.381
SRP9	0.00407	0.383
KLF13	0.00133	0.384
PFKFB4	0.00943	0.385
TRA2A	0.00121	0.385
YAE1D1	6.79E-06	0.385
HINT2	0.00332	0.386
NME5	0.00852	0.387
TLR2	0.00783	0.387
SMYD3	0.00635	0.388
ALDH1A2	0.0059	0.389
PEG3	0.00267	0.39
PRPS1	0.000339	0.39
INTS3	2.00E-04	0.391
MME	0.000666	0.391
SHC4	0.000286	0.391
ATP6AP1	5.13E-05	0.392
LAT2	2.27E-05	0.392
DMKN	0.00402	0.393
DYNLRB2	0.00364	0.393
MICAL3	0.000626	0.393
RGPD4	0.000387	0.393
ZC3HAV1	0.000436	0.393

CA11	0.000189	0.394
TBCK	0.00377	0.394
CEP19	0.00072	0.395
GLIPR1	6.50E-05	0.395
TRIM22	0.000568	0.395
42248	0.0042	0.396
DUSP19	3.45E-05	0.396
PRCP	0.00394	0.397
WBP1	0.00331	0.397
CTSA	0.00951	0.398
SH2B3	4.00E-04	0.398
DPCD	5.71E-05	0.399
FADS1	0.00642	0.399
MORC4	0.00832	0.399
MDP1	0.000281	0.401
ZDHHC22	4.83E-06	0.402
APLP2	0.00399	0.403
COL6A1	0.00634	0.403
CYB5D1	0.00459	0.403
LZTFL1	0.00442	0.405
ARHGEF37	0.00111	0.406
FAM3A	0.00549	0.406
CCDC113	0.000684	0.407
FEZ1	0.00072	0.407
C1orf123	0.000906	0.408
CTSF	0.00381	0.408
IDH1	0.00933	0.408
TNFSF15	0.00115	0.408
IARS	0.000302	0.409
TUB	0.00204	0.409
C6orf62	0.00166	0.411
CD53	0.000997	0.411
HSPB2	0.0074	0.411
KHDRBS1	0.00487	0.412
RNF182	0.00376	0.412
ANKLE1	0.000269	0.414
BDH2	0.00389	0.414
EXO5	0.00347	0.414
COX11	0.00671	0.415
ISLR2	0.000318	0.415
KMT5B	0.0066	0.416
METTL18	0.00227	0.417
CDK2AP1	0.00236	0.418
REC8	0.00101	0.418
NDUFB10	0.00266	0.419

PSMA1	0.000981	0.419
ZNF106	0.0026	0.419
RBBP7	0.000983	0.42
TNFRSF11B	0.00255	0.42
B3GALT6	0.00442	0.422
EIF4G2	0.00234	0.422
FHIT	1.07E-05	0.423
FAP	8.19E-07	0.424
TMEM147	0.0065	0.424
LAGE3	0.00566	0.425
SPATA33	2.65E-05	0.425
SIVA1	0.00593	0.426
HYI	0.00128	0.427
POLR2G	0.00103	0.427
SLC35A5	0.00183	0.427
SCGB3A2	0.000758	0.428
YIF1A	0.000624	0.428
SERINC1	0.0036	0.429
SLC2A10	0.00276	0.43
CPNE3	0.000692	0.431
GPR155	0.000188	0.431
LSM5	0.000448	0.431
RNF130	6.79E-05	0.431
RUSC1	0.0041	0.431
COMMD3	0.0069	0.432
TFAP2B	0.00903	0.432
SCN11A	0.000141	0.433
RERG	3.21E-06	0.434
CBX7	0.00409	0.436
WDR61	3.05E-05	0.436
CD81	0.00129	0.437
FKBP14	0.00591	0.437
YWHAH	0.00879	0.437
LRRN1	0.000236	0.438
ZNF618	0.00318	0.438
CTSZ	0.00109	0.439
CLDN10	0.000234	0.44
COL4A2	0.00903	0.44
FGFRL1	0.00048	0.44
WFS1	0.000417	0.44
HNRNPH3	3.09E-05	0.441
NBL1	0.00608	0.441
PRDX4	0.000142	0.441
RSPO2	0.00111	0.441
SCRG1	2.51E-05	0.441

DHRS7	0.00243	0.442
MRTO4	5.35E-05	0.442
TEP1	0.00023	0.443
DFNA5	0.00744	0.445
PDZD4	0.000361	0.445
TMEM42	1.56E-06	0.445
APEX1	0.00533	0.446
RTN1	0.0018	0.446
CREB1	2.63E-06	0.447
IFT20	0.00385	0.448
TCN2	0.0053	0.448
TMEM263	0.00112	0.449
EIF3L	0.00304	0.45
GTF2A2	0.000879	0.45
IDI1	0.000789	0.45
WDR82	1.01E-05	0.45
ACVR1	0.00505	0.451
CHPF	0.00341	0.451
P4HA1	0.000361	0.451
ACAD11	0.00315	0.453
AMIGO2	0.000112	0.453
ASXL1	0.00463	0.453
MYOZ1	0.00011	0.453
ARMCX2	0.000111	0.455
HNMT	0.00168	0.455
KCNK15	0.00499	0.455
MMAB	0.00122	0.455
AHCYL1	0.00264	0.456
GUSB	3.10E-05	0.456
KIF1C	0.00833	0.457
MRPL49	9.95E-05	0.457
RTN4	0.000598	0.457
SLC35E1	0.0018	0.458
ATP1B2	8.52E-05	0.459
NAT14	0.00138	0.459
COPRS	0.000529	0.46
HECW2	0.0014	0.461
NDUFB5	0.0013	0.461
GPRASP2	0.00014	0.462
NDUFC1	0.000228	0.463
ATRAID	0.00183	0.464
KIF7	3.31E-05	0.464
SPATS2L	0.000205	0.464
RGS5	0.00432	0.465
GAL3ST3	0.000116	0.466

KDELC2	0.000392	0.466
CCL3L1	0.00144	0.467
CECR1	0.00314	0.467
D2HGDH	0.00978	0.467
MAGED4B	0.000559	0.467
RPA1	0.00637	0.467
MAF	0.00173	0.468
CARD9	3.91E-07	0.47
SFRP2	0.000485	0.47
C5orf24	0.000689	0.471
SKP2	7.60E-06	0.471
ZNF540	0.000167	0.471
HSD11B1L	0.000865	0.472
NDUFA4	0.00142	0.472
SLC25A44	0.000902	0.472
CALY	0.000165	0.473
SLC20A2	1.68E-05	0.473
ZNF454	0.000221	0.473
MRPS21	0.000644	0.474
PITPNM1	0.00199	0.474
TMEM98	0.000235	0.475
CYYR1	6.48E-07	0.476
NPIPA1	0.00135	0.476
STAMBPL1	3.15E-06	0.476
KIAA0930	0.000617	0.477
MZF1	0.000954	0.477
HYAL2	0.000277	0.478
ITIH3	3.94E-07	0.479
TMEM126B	0.000181	0.479
RPA2	0.000422	0.48
MATR3	0.00852	0.481
MBTPS1	0.00192	0.481
PRKAR1A	0.00144	0.481
HPD	0.000604	0.482
KIFC3	0.00122	0.482
C9orf3	0.001	0.483
NCF4	0.00237	0.484
YIPF5	3.73E-05	0.486
SLC35E2B	0.000208	0.487
LAPTM4A	0.00194	0.489
PIGP	0.000552	0.489
ATP10D	0.00197	0.49
HEG1	5.70E-06	0.492
XAGE2	0.00153	0.493
SRI	0.000592	0.494

SOD1	0.00491	0.495
TNK2	1.98E-05	0.495
LYRM7	0.000741	0.497
PRSS23	1.65E-05	0.497
LY6E	0.000453	0.499
IMP3	0.000414	0.5
PPP1R3C	8.00E-04	0.5
CCDC53	5.84E-06	0.501
CD14	0.000235	0.501
GLB1	0.00316	0.501
LYVE1	1.52E-05	0.501
CRELD2	6.19E-05	0.502
SCARF2	0.00171	0.502
SLC20A1	3.63E-05	0.505
ALAS2	8.57E-07	0.506
LHFPL2	0.00355	0.507
ERAP2	1.40E-06	0.508
EXT2	2.73E-06	0.508
MPEG1	0.000128	0.508
BCL2L13	0.00148	0.509
SRSF1	0.00292	0.511
ANXA5	0.000681	0.512
SMPX	4.89E-07	0.512
PODXL	6.94E-05	0.514
C11orf1	0.00111	0.515
KAZALD1	0.00518	0.516
RHOBTB3	0.0027	0.517
ZRANB2	0.00035	0.517
PIK3CD	0.000103	0.52
AKR1B1	0.00246	0.521
VKORC1	0.000772	0.521
TSPAN3	0.0024	0.522
C2orf15	3.02E-05	0.523
GUK1	0.00318	0.524
TMEM160	0.00116	0.524
ADORA1	0.000564	0.525
P4HB	0.00633	0.526
DPYSL2	0.000183	0.53
FCGR3A	0.00364	0.53
PNMA1	0.000817	0.53
PYURF	0.00222	0.53
AHSA2	8.52E-05	0.532
KDELR3	5.99E-06	0.534
POLE4	0.00133	0.536
DCN	0.00149	0.537

FCHSD2	0.0064	0.537
IFIT3	0.00938	0.537
ABHD14A	5.77E-07	0.538
B3GALNT1	0.000161	0.538
NCF1	0.000473	0.538
ARCN1	0.000571	0.539
CTHRC1	0.00653	0.539
TMEM59L	0.000551	0.54
UST	4.97E-07	0.54
ZNF136	0.00483	0.541
LYL1	0.000431	0.542
CGNL1	0.00208	0.543
EMP3	5.25E-05	0.543
IFI44	2.24E-05	0.543
LY96	0.00424	0.543
ACTG1	0.00817	0.544
IL17D	9.50E-05	0.546
ESD	0.00229	0.547
MDK	0.00646	0.548
PCDH12	1.59E-08	0.548
TRIP6	3.31E-05	0.548
LTBP3	7.79E-06	0.549
TMEM245	0.000104	0.549
CD99L2	1.14E-05	0.55
HNRNPA1	0.00948	0.551
PSIP1	8.69E-06	0.551
S100A13	0.0022	0.551
KCNMB1	0.0083	0.553
PLA2G5	9.05E-06	0.553
ATP6AP2	0.00164	0.554
CPE	0.00154	0.554
DKK3	1.62E-05	0.554
LDOC1L	1.15E-06	0.555
TCEAL2	0.00405	0.558
GSTM5	0.000232	0.559
LTC4S	0.00193	0.559
KLKB1	7.17E-05	0.56
TIMP2	5.78E-06	0.56
DNASE1L3	0.000609	0.562
GNAS	0.000922	0.564
PKD2	1.14E-06	0.564
NEFH	0.000326	0.565
CBX6	0.00136	0.567
RGS4	0.00262	0.567
HLA-DMA	0.00368	0.568

P4HA2	3.10E-05	0.572
SEMA3E	1.19E-05	0.572
ISLR	0.00546	0.574
LY86	1.88E-06	0.574
MRAS	0.00384	0.574
PPIC	1.07E-05	0.574
ZNF665	0.00577	0.574
C21orf33	0.000728	0.579
SYT11	0.00137	0.579
FAM189A2	4.22E-05	0.58
SLC2A5	3.82E-06	0.582
GAP43	6.86E-06	0.584
CD83	0.000207	0.585
HMGN2	7.32E-05	0.585
SIGIRR	0.00467	0.585
SAC3D1	0.000115	0.589
COPS8	6.86E-05	0.591
APBB3	7.23E-05	0.593
EFEMP2	0.000358	0.594
HCAR1	8.22E-05	0.596
COPZ2	1.01E-06	0.599
ARF4	3.08E-05	0.6
KIAA0355	0.00135	0.6
ARAP3	0.000292	0.603
DDAH1	0.0097	0.603
HNRNPM	0.00878	0.603
ADORA3	0.00377	0.604
KIAA1755	0.00657	0.605
MS4A6A	3.45E-05	0.605
DNAJB2	0.00677	0.606
SLC4A3	1.32E-05	0.611
GALT	8.45E-05	0.613
NDN	7.13E-05	0.614
PCDH7	0.000993	0.614
SSR4	0.00191	0.614
FCGRT	0.00017	0.616
TREM2	4.14E-06	0.618
FLI1	1.92E-06	0.621
SPARC	0.000666	0.626
SNRPE	0.00264	0.627
IFIT1	2.69E-10	0.631
PRRT2	0.00578	0.631
ZNF549	0.00471	0.631
C5	1.83E-05	0.634
SHISA4	2.02E-05	0.634

MOK	0.000229	0.635
SULF2	0.000199	0.637
CRABP2	0.00038	0.64
MXRA5	0.00511	0.64
PIK3CG	0.0014	0.64
MSX1	0.000503	0.642
HLA-C	0.00072	0.645
CD99	2.50E-06	0.647
LTB	0.00307	0.648
PLS3	5.57E-06	0.648
ZNF671	0.000918	0.649
PAMR1	0.00271	0.651
ZSCAN18	6.93E-05	0.652
FAM156A	1.20E-05	0.653
TTC3	1.60E-05	0.654
PPT1	1.49E-09	0.656
CDC26	0.000136	0.657
MXRA7	9.91E-05	0.658
TM2D2	5.59E-06	0.658
PINK1	0.000951	0.659
ALDOC	0.0021	0.66
SEMA6D	0.000457	0.66
COL1A2	3.38E-06	0.662
UBB	0.0035	0.665
PLA2G16	0.00139	0.666
IL13RA2	0.00705	0.672
MUM1	0.00013	0.674
C1orf54	0.000137	0.676
RPS27L	0.000227	0.678
TNNC1	0.00123	0.679
COTL1	6.85E-05	0.681
TCEAL5	0.000917	0.682
MX1	0.000662	0.683
PRELP	0.00269	0.683
RPL15	0.000447	0.683
VPS41	5.43E-08	0.685
ACOX2	1.19E-05	0.686
FBLN5	0.000372	0.686
HCST	0.00104	0.688
LRRFIP1	0.00164	0.688
CACNG3	5.26E-06	0.69
ITM2B	0.00817	0.692
MTMR11	1.08E-06	0.692
CPXM2	0.00109	0.695
CTNNBL1	0.000166	0.695

GLT1D1	2.05E-05	0.695
MPC2	9.94E-05	0.699
PALD1	7.57E-07	0.699
REXO2	0.00451	0.7
SMOC1	0.000129	0.7
ALKBH7	3.87E-05	0.701
GSN	0.00287	0.701
HLA-DQB1	0.000796	0.702
UROD	0.000838	0.702
BEX3	0.00473	0.704
SDC3	0.000217	0.704
CRLF1	5.04E-05	0.705
F5	6.58E-06	0.707
MRC2	0.000406	0.707
PDGFRB	0.00425	0.708
FZD1	1.26E-06	0.71
MAGED2	1.55E-06	0.713
GMDS	0.00594	0.715
FCER1G	0.000848	0.717
MEGF10	0.000359	0.718
LTBP2	0.00124	0.72
NDUFB2	0.00245	0.72
C1orf122	0.0014	0.722
GABBR1	0.000133	0.725
OLFML2B	0.000237	0.726
PBXIP1	0.000301	0.726
HEPH	4.90E-05	0.728
PLCE1	3.68E-07	0.729
TSPAN6	1.19E-05	0.736
ESYT1	0.000223	0.738
PPM1F	9.63E-07	0.739
VENTX	0.000187	0.741
TUBA1A	8.12E-07	0.746
PON2	0.000281	0.747
RNASE4	0.000297	0.747
TTC25	7.82E-07	0.747
VCAM1	0.00816	0.747
ADAP2	0.00155	0.748
AQP1	0.000686	0.748
PHPT1	0.000824	0.748
LDHA	4.77E-06	0.751
TCTN1	1.40E-07	0.751
PDGFD	0.000374	0.752
COLEC12	3.87E-06	0.753
IGFBP7	0.00411	0.753

MAGED1	1.78E-06	0.757
MNDA	3.22E-10	0.761
MYH10	2.49E-05	0.761
CHST6	1.21E-07	0.762
COL5A1	3.29E-05	0.764
APLNR	1.52E-06	0.766
RIMBP2	0.000169	0.769
SERPING1	0.0027	0.774
MEGF6	0.0011	0.779
PAQR8	0.000102	0.782
FGD6	1.26E-06	0.791
DYNC1I1	6.43E-06	0.794
IGSF6	1.70E-05	0.796
POLR2L	0.0053	0.797
NUAK1	5.90E-06	0.799
CYBRD1	0.000386	0.801
NDUFB8	0.000149	0.801
THBD	3.53E-05	0.804
NAV1	0.000207	0.81
GLS	2.37E-05	0.811
NISCH	6.22E-06	0.811
HSPA8	3.51E-05	0.819
TNS3	1.16E-05	0.82
ARHGEF6	4.99E-05	0.821
IFI44L	8.10E-09	0.829
CIRBP	0.000734	0.832
CPQ	2.65E-06	0.846
TRO	0.000352	0.847
NCAM1	5.99E-07	0.849
IFIT2	3.92E-07	0.851
ATP5J	0.00485	0.854
DAD1	0.000858	0.855
LAYN	2.74E-06	0.857
JAM3	5.71E-07	0.864
TMEM119	7.14E-06	0.866
SHOX	0.00118	0.869
PTN	0.00937	0.875
CST3	0.0027	0.876
OLFML3	0.000304	0.88
CCL3	0.000132	0.881
HLA-DPB1	4.00E-04	0.883
CSF1R	3.70E-05	0.885
DOK1	0.000231	0.887
TMEM130	6.14E-06	0.89
LEFTY2	6.21E-07	0.892

COL4A4	2.49E-06	0.896
C21orf62	2.13E-05	0.9
MARCO	0.000259	0.901
STMN2	0.00273	0.902
CLDN11	5.01E-05	0.912
ALDH7A1	7.97E-05	0.916
CST6	2.13E-08	0.919
SLC24A3	0.000149	0.923
CEMIP	0.00126	0.937
COLGALT2	0.000324	0.937
SARAF	9.23E-05	0.939
C1QA	0.000943	0.94
KCNAB1	0.00204	0.942
ITGB5	1.67E-07	0.949
TGFB3	7.73E-06	0.951
VAMP5	0.000371	0.952
FGF9	0.000569	0.96
GP1BB	0.000304	0.962
SLC16A9	8.03E-06	0.964
ITGBL1	3.91E-06	0.965
FN1	9.85E-09	0.967
COL3A1	7.53E-05	0.972
RPS12	0.00913	0.973
MATN2	1.24E-05	0.974
CYTH4	9.20E-06	0.978
VOPP1	3.01E-07	0.982
ENO1	0.00343	0.985
DYNLL1	1.44E-06	0.988
HLA-DQA1	0.000718	0.993
COL5A2	1.20E-06	0.994
C1QTNF3	5.88E-09	1.01
CHST15	0.000516	1.01
HEY1	0.00117	1.01
ZDHHC14	2.98E-05	1.01
FRZB	0.00028	1.03
NEB	5.65E-05	1.03
VIM	0.000921	1.04
ANKH	5.78E-10	1.05
CLEC11A	2.75E-06	1.05
COCH	4.41E-05	1.05
MGARP	1.00E-04	1.05
MXRA8	1.36E-06	1.05
HLA-DMB	0.00032	1.06
TNFRSF25	3.99E-07	1.06
OLFML1	1.44E-06	1.07

EPDR1	1.12E-05	1.09
ID3	0.000504	1.09
MCHR1	3.37E-05	1.09
TPM2	0.00337	1.1
TYROBP	4.35E-05	1.12
AIF1	5.21E-05	1.13
CEND1	0.00028	1.13
GPHA2	0.000475	1.13
TAGLN	0.00264	1.13
BCL6B	8.02E-06	1.14
SEC61G	0.00229	1.14
ALOX5AP	0.000496	1.15
FADS2	8.26E-09	1.16
LUM	2.79E-06	1.17
COL8A2	1.71E-06	1.18
ITM2C	1.79E-08	1.18
SCD	5.24E-07	1.18
IGFBP6	2.54E-06	1.22
CCDC3	6.38E-08	1.23
MAOB	1.25E-06	1.26
RASL11B	1.98E-07	1.27
PCOLCE	4.53E-07	1.28
HTRA1	2.78E-07	1.31
CD74	1.01E-05	1.33
CA12	2.81E-08	1.35
CTGF	0.000409	1.35
CELF5	3.91E-05	1.4
CDH2	0.000111	1.41
CDH23	8.77E-06	1.41
NELL2	4.35E-08	1.41
GRP	0.000375	1.43
SLC4A11	0.000116	1.54
HBG1	2.48E-06	1.55
HBG2	2.60E-06	1.55
IGFBP2	1.68E-05	1.55
OGN	1.45E-05	1.58
LGALS1	1.84E-05	1.6
PTGDS	1.53E-07	1.63
FMOD	5.04E-07	1.64
CYTL1	9.96E-07	1.69
HLA-DPA1	9.46E-09	1.84
C2orf40	5.38E-10	1.98
FCGBP	8.61E-08	2.15
MGP	3.23E-07	2.39
HBD	7.26E-19	3.54

HBB	2.03E-19	4.98
HBA1	5.25E-17	5.05
HBA2	2.07E-18	5.43
