# Supplementary material for: Novel potential drugs for the treatment of primary open-angle glaucoma using protein-protein interaction network analysis
Source: Genomics Inform. 2023 Mar 31;21(1):e6. doi: 10.5808/gi.22070 (PMC10085733; doi:10.5808/gi.22070)
Supplement: Supplementary Table 2. — Upregulated genes based on Reactome pathway enrichment analysis [file gi-22070-Supplementary-Table-2.pdf]

Supplementary Table 2. Upregulated genes based on Reactome pathway enrichment analysis

Reactome pathway	p-value	Genes
Extracellular matrix organization	3.13E-07	*COLGALT2, SPARC, ITGAM, ITGB5, SDC3, HTRA1, LTBP2, LTBP3, FBLN5, LOXL1, EFEMP2, TIMP2, CAPN2, NCAM1, JAM3, VCAM1, TGFB3, LUM, FN1, PCOLCE, DCN, COL3A1, COL1A2, COL4A2, COL5A1, P4HA1, P4HA2, COL4A4, COL5A2, COL6A1, COL8A2, P4HB, FMOD, KLKB1*
Glycosaminoglycan metabolism	8.15E-07	*CSGALNACT1, CHST6, CEMIP, CHPF, LUM, GLCE, SDC3, B3GALT6, PRELP, LYVE1, DCN, EXT2, GLB1, HYAL2, OGN, UST, CHST15, GPC5, GUSB, FMOD*
ECM proteoglycans	2.49E-06	*SPARC, ITGB5, TGFB3, LUM, FN1, DCN, COL3A1, COL1A2, COL4A2, COL5A1, COL4A4, COL5A2, COL6A1, NCAM1, FMOD*
Metabolism of carbohydrates	1.52E-05	*PRPS1, PFKFB4, CEMIP, GALT, CHPF, SDC3, AKR1B1, PRELP, ENO1, FUT6, UBB, HYAL2, CHST15, GPC5, GUSB, CSGALNACT1, CHST6, LUM, GLCE, GAA, B3GALT6, LYVE1, DCN, EXT2, PPP1R3C, GLB1, OGN, UST, ALDOC, FMOD*
Immune system	1.63E-05	*AHCYL1, NCF1, TMEM179B, CD81, NCF4, IFIT1, IFIT3, ACTG1, IFIT2, CDC26, COTL1, CPNE3, IL13RA2, SKP2, TRIM22, FBXO9, WSB1, HLA-C, CLEC4A, SIGIRR, TYROBP, HECW2, SERPING1, VCL, CSF1R, CCL3L1, LY96, TNFRSF11B, C5, PRDX4, HNRNPDL, UBB, NDN, CD14, SLC15A4, HLA-DQA1, CD99, CTSA, HSPA8, PTPRN2, VCAM1, TNFSF15, IDH1, FN1, LY86, PPBP, COL1A2, RNF182, HLA-DRA, EIF4G2, ITGAM, ITGB5, CTSZ, LPCAT1, PIK3CD, HBB, TREM2, SLC2A5, PIK3CG, TNFSF13B, MRC2, TRIM9, GLIPR1, FCGR3A, TUBA1A, SEC61G, TIMP2, CTSF, LRRFIP1, GUSB, HLA-DPA1, CD53, FCER1G, MME, GAA, SFTPD, ATP6AP2, IL18, COMMD3, ANO6, DYNLL1, LAT2, CREB1, PSMA1, TNFRSF25, ALDOC, LTB, DOCK2, HCST, HLA-DQB1, TLR2, COLEC12, DCTN6, C1QA, RNF34, PRCP, CST3, PGRMC1, HLA-DMB, CCL3, NCAM1, RNF130, SH2B3, DYNC1I1, POLR2L, TRIM45, CD74, GSN, ERAP2, IL34, CARD9, MX1, SOD1, COL3A1, GLB1, HLA-DPB1, MNDA, VIM, P4HB, ACTR10, CRLF1*
Collagen biosynthesis and modifying enzymes	1.76E-05	*COLGALT2, PCOLCE, COL3A1, COL1A2, COL4A2, COL5A1, P4HA1, P4HA2, COL4A4, COL5A2, COL6A1, COL8A2, P4HB*
Diseases associated with glycosaminoglycan metabolism	3.72E-05	*EXT2, CHST6, LUM, SDC3, OGN, GPC5, B3GALT6, PRELP, FMOD, DCN*
Binding and uptake of ligands by scavenger receptors	4.56E-05	*COLEC12, MARCO, COL3A1, SPARC, COL1A2, COL4A2, SCGB3A2, HBB, HBA2, HBA1*
Integrin cell surface interactions	4.57E-05	*ITGAM, VCAM1, ITGB5, LUM, FN1, COL3A1, COL1A2, COL4A2, COL5A1, COL4A4, COL5A2, COL6A1, COL8A2, JAM3*
Neutrophil degranulation	5.16E-05	*ITGAM, TMEM179B, CTSZ, LPCAT1, HBB, PRCP, SLC2A5, CST3, GLIPR1, PGRMC1, PRDX4, TIMP2, COTL1, CPNE3, CD14, GUSB, SLC15A4, CD53, CTSA, HSPA8, FCER1G, PTPRN2, GSN, MME, IDH1, GAA, ATP6AP2, HLA-C, COMMD3, ANO6, PPBP, DYNLL1, TYROBP, GLB1, ALDOC, MNDA, DOCK2, ACTR10, VCL, TLR2*
Collagen formation	8.41E-05	*COLGALT2, PCOLCE, LOXL1, COL3A1, COL1A2, COL4A2, COL5A1, P4HA1, P4HA2, COL4A4, COL5A2, COL6A1, COL8A2, P4HB*
Platelet degranulation	8.81E-05	*LGALS3BP, ITIH3, SPARC, TGFB3, PCDH7, APLP2, ANXA5, FN1, PPBP, LHFPL2, F5, SOD1, ISLR, SERPING1, MAGED2, LEFTY2, VCL*
Diseases of metabolism	1.11E-04	*GALT, SDC3, PRELP, UBB, GPC5, GUSB, CTSA, CHST6, LUM, MMAB, ABCA3, IDH1, GAA, SFTPD, B3GALT6, POMGNT1, DCN, EXT2, LMBRD1, PPP1R3C, TCN2, GLB1, OGN, FMOD, CD320*

Response to elevated platelet cytosolic Ca^{2+}	1.39E-04	*LGALS3BP, ITIH3, SPARC, TGFB3, PCDH7, APLP2, ANXA5, FN1, PPBP, LHFPL2, F5, SOD1, ISLR, SERPING1, MAGED2, LEFTY2, VCL*
Defective CHST6 causes MCDC1	1.82E-04	*CHST6, LUM, OGN, PRELP, FMOD*
Hemostasis	1.89E-04	*DOCK6, LGALS3BP, ITIH3, SPARC, ITGAM, SDC3, HBB, PRCP, CD99L2, HBD, SRI, PIK3CG, THBD, ISLR, TUBA1A, KCNMB1, KIF1C, LEFTY2, SH2B3, CD99, JAM3, CD74, FCER1G, HBG2, TGFB3, HBG1, GP1BB, APLP2, PCDH7, ANXA5, FN1, LHFPL2, PPBP, ATP1B2, F5, SOD1, COL1A2, PRKAR1A, GNAS, SERPING1, CD48, MAGED2, TEK, DOCK2, KLKB1, VCL*
Regulation of IGF transport and uptake by IGFBPs	6.90E-04	*MBTPS1, WFS1, APLP2, IGFBP2, FN1, PDIA6, PRSS23, F5, CST3, LGALS1, CDH2, MXRA8, IGFBP7, IGFBP6, P4HB*
Syndecan interactions	7.09E-04	*COL3A1, COL1A2, COL5A1, ITGB5, SDC3, COL5A2, FN1*
Scavenging by class A receptors	8.78E-04	*COLEC12, MARCO, COL3A1, COL1A2, COL4A2, SCGB3A2*
Assembly of collagen fibrils and other multimeric structures	8.80E-04	*COL3A1, COL1A2, COL4A2, COL5A1, COL4A4, COL5A2, COL6A1, COL8A2, PCOLCE, LOXL1*
Keratan sulfate degradation	0.001574	*GLB1, LUM, OGN, PRELP, FMOD*
Post-translational protein phosphorylation	0.00175	*MBTPS1, WFS1, APLP2, FN1, PDIA6, PRSS23, F5, CST3, LGALS1, CDH2, MXRA8, IGFBP7, P4HB*
MHC class II antigen presentation	0.001809	*CTSA, DCTN6, CD74, DYNLL1, HLA-DMB, TUBA1A, HLA-DPB1, HLA-DRA, CTSF, ACTR10, HLA-DQA1, DYNC1I1, HLA-DPA1, HLA-DQB1*
Elastic fiber formation	0.002091	*EFEMP2, ITGB5, TGFB3, FN1, LTBP2, LTBP3, LOXL1, FBLN5*
Collagen chain trimerization	0.002091	*COL3A1, COL1A2, COL4A2, COL5A1, COL4A4, COL5A2, COL6A1, COL8A2*
Non-integrin membrane-ECM interactions	0.002922	*COL3A1, COL1A2, COL4A2, COL5A1, ITGB5, COL4A4, SDC3, COL5A2, FN1*
Defective B4GALT1 causes B4GALT1-CDG (CDG-2d)	0.003439	*LUM, OGN, PRELP, FMOD*
Defective ST3GAL3 causes MCT12 and EIEE15	0.003439	*LUM, OGN, PRELP, FMOD*
Molecules associated with elastic fibers	0.003918	*EFEMP2, ITGB5, TGFB3, FN1, LTBP2, LTBP3, FBLN5*
Chondroitin sulfate/dermatan sulfate metabolism	0.0044	*CSGALNACT1, CHPF, SDC3, UST, CHST15, GPC5, B3GALT6, DCN*
Adaptive immune system	0.004965	*COLEC12, DCTN6, RNF34, AHCYL1, ITGB5, NCF1, CD81, NCF4, LY96, PIK3CD, TREM2, MRC2, TRIM9, FCGR3A, HLA-DMB, TUBA1A, UBB, CDC26, SEC61G, CTSF, CD14, SKP2, RNF130, CD99, HLA-DQA1, DYNC1I1, FBXO9, HLA-DPA1, CTSA, CD74, WSB1, VCAM1, ERAP2, SFTPD, HLA-C, DYNLL1, COL3A1, TYROBP, COL1A2, PSMA1, HECW2, HLA-DPB1, RNF182, HLA-DRA, ACTR10, HCST, HLA-DQB1, TLR2*
Erythrocytes take up oxygen and release carbon dioxide	0.004999	*HBB, HBA2, HBA1, AQP1*
Degradation of the extracellular matrix	0.00559	*HTRA1, FN1, DCN, COL3A1, COL1A2, COL4A2, COL5A1, COL4A4, COL5A2, COL6A1, TIMP2, COL8A2, CAPN2, KLKB1*
Crosslinking of collagen fibrils	0.00571	*COL1A2, COL4A2, COL4A4, PCOLCE, LOXL1*

Innate immune system	0.006612	*AHCYL1, ITGAM, NCF1, CD81, TMEM179B, NCF4, CTSZ, LPCAT1, HBB, TREM2, SLC2A5, ACTG1, GLIPR1, FCGR3A, TIMP2, COTL1, CPNE3, LRRFIP1, GUSB, CD53, FCER1G, MME, GAA, SFTPD, ATP6AP2, HLA-C, COMMD3, ANO6, DYNLL1, LAT2, CLEC4A, SIGIRR, TYROBP, CREB1, PSMA1, SERPING1, ALDOC, DOCK2, VCL, TLR2, C1QA, LY96, PRCP, CST3, C5, PGRMC1, PRDX4, UBB, CD14, SLC15A4, POLR2L, CTSA, HSPA8, PTPRN2, GSN, IDH1, CARD9, LY86, PPBP, GLB1, MNDA, ACTR10*
Diseases of glycosylation	0.006666	*CTSA, CHST6, GALT, LUM, SDC3, B3GALT6, PRELP, POMGNT1, DCN, EXT2, GLB1, OGN, GPC5, FMOD*
Platelet activation, signaling, and aggregation	0.00692	*LGALS3BP, ITIH3, SPARC, FCER1G, TGFB3, GP1BB, PCDH7, APLP2, ANXA5, FN1, PPBP, LHFPL2, PIK3CG, F5, SOD1, ISLR, COL1A2, SERPING1, MAGED2, LEFTY2, VCL*
Translocation of ZAP-70 to immunological synapse	0.006998	*HLA-DPB1, HLA-DRA, HLA-DQA1, HLA-DPA1, HLA-DQB1*
NCAM1 interactions	0.007423	*COL3A1, COL4A2, COL5A1, COL4A4, COL5A2, COL6A1, NCAM1*
Heparan sulfate/heparin (HS-GAG) metabolism	0.007483	*EXT2, GLB1, GLCE, SDC3, GPC5, B3GALT6, GUSB, DCN*
PDGF signaling	0.009969	*PDGFRB, COL3A1, COL4A2, COL5A1, COL4A4, PDGFD, COL5A2, COL6A1*

ECM, extracellular matrix; IGF, insulin-like growth factor; IGFBP, IGF binding protein; MHC, major histocompatibility complex; PDGF, platelet-derived growth factor.
