# Supplementary material for: Novel potential drugs for the treatment of primary open-angle glaucoma using protein-protein interaction network analysis
Source: Genomics Inform. 2023 Mar 31;21(1):e6. doi: 10.5808/gi.22070 (PMC10085733; doi:10.5808/gi.22070)
Supplement: Supplementary Table 3. — Upregulated genes based on molecular function enrichment analysis [file gi-22070-Supplementary-Table-3.pdf]

Supplementary Table 3. Upregulated genes based on molecular function enrichment analysis

Molecular function	p-value	Genes
Protein binding	2.66E-09	*ZNF177, C5ORF24, ANKLE1, STMN2, MSANTD4, ENO1, ALKBH7, SLC4A3, GLS, DPYSL2, ANKFY1, PLCE1, WSB1, CLEC11A, DKK3, SIGIRR, NISCH, FAM133A, PRKAR1A, WDR82, TEP1, HECW2, FEZ1, AKIP1, SERPING1, TMEM126B, CRABP2, MRPL18, ZC3HAV1, GABARAP, C5, LDHA, PRDX4, NTHL1, S100A13, CHST15, HLA-DQA1, ZSCAN18, FZD1, ZNF264, PRRT2, SIVA1, ST13, TMEM190, FN1, RMDN3, FLI1, HNRNPM, COL1A2, TMEM98, TCN2, TRIP6, PPT1, PSMG1, ZNF136, GSTM5, ARF4, SMG1, PIGT, PIGP, PRSS23, MRC2, C1QTNF3, TRIM9, TUBA1A, HEY1, RSPO2, TIMP2, GPRASP2, NEFH, CGNL1, ANXA4, TNNC1, ANXA5, MRPL49, TMEM42, EMP3, DYNLL1, TERF1, SRP9, F5, MS4A6A, MRAS, BDH2, PSMA1, KIFC3, VAMP5, HCST, HLA-DQB1, PCYOX1, GRP, RPS27L, TMEM160, METTL21A, MRPL57, HLA-DMA, HLA-DMB, SLC17A9, FRZB, RPL15, GPHA2, JAM3, YTHDF1, TIMMDC1, GSN, CARD9, ARCN1, COL3A1, DHRS7, SNX19, APEX1, SMOC1, LYRM7, HLA-DPB1, SCRG1, CYB561, ZNF213, KLKB1, CRLF1, NCF1, RASL11B, TMEM179B, CEP19, NCF4, UBP1, EFEMP2, LAPTM4A, APMAP, CDH2, FCGRT, UROD, MPC1, MPC2, ADORA1, KMT5B, SLC16A9, HMGN2, IL13RA2, ZNF440, IGFBP2, VASH1, MAF, MRTO4, SNRPE, PHPT1, FAM8A1, LY6E, SHC4, CSF1R, KHDRBS1, PFKFB4, CEMIP, KHDRBS3, CCDC24, RHOBTB3, LY96, TNFRSF11B, FHIT, ACAT2, BCL2L13, ADAP2, HNRNPDL, ZNF549, ESD, NDN, HYI, IGFBP7, SMYD3, IGFBP6, SLC15A3, SLC15A4, SAC3D1, ZNF540, WFS1, TNK2, LY86, CYBRD1, NEB, LSM5, COPRS, ZC3HC1, COPS4, TMEM17, P4HA1, REEP2, OGN, RNF182, HLA-DRA, CPE, SERINC1, COPS8, EIF4G2, MAP3K12, ALAS2, ITGAM, ITGB5, DUSP19, PIK3CD, CELF5, TREM2, ATRAID, PIK3CG, TNFSF13B, TRO, PAGR1, THBD, FCGR3A, MDK, GAL3ST3, METTL18, CTNNBL1, SNAP47, NFAT5, PRPS1, ITIH3, GTF2A2, RNF34, WBP1, NDUFB5, SRSF1, ATP10D, PRCP, SEMA3E, PBXIP1, SRI, LAGE3, DPCD, PPM1F, COCH, DNAJB2, CCL8, CCL3, POLR2G, RBBP7, HNRNPA1, SH2B3, POLR2L, NDUFA4, MMAB, FOXJ1, REC8, ASXL1, NELL2, PINK1, ZNF618, EIF3L, SYT11, MGP, VPS41, PDCD2, MNDA, VIPAS39, P4HB, AHCYL1, SPARC, IFIT1, CYB5D2, IFIT3, AQP1, IFIT2, LGALS1, RUSC1, CDC26, CPNE3, VSIG4, ACAD11, MYOZ1, CCDC92, SKP2, PTGDS, TNS3, SLC35A5, TRIM22, RPS12, FBXO9, ACVR1, PAQR8, IMP3, CIRBP, HLA-C, ISLR2, PPP1R3C, MXRA8, MGARP, ALDH7A1, NBL1, SLC22A4, BEX3, L3MBTL2, MAOB, MAGED1, SDC3, CEND1, CD99L2, NUAK1, TSPAN6, SARAF, CD14, ZRANB2, SLC35E1, TNFSF15, INTS3, IFI44, PYCR2, PPBP, PAICS, EXT2, CALY, ID3, LRRN1, MAGED2, ITM2B, CD320, ITM2C, FKBP14, CTSZ, AKR1B1, HBB, HBD, GLIPR1, SLC22A17, GUK1, HYAL2, SEC61G, KIF1C, SDF4, LMCD1, VENTX, VKORC1, CD53, FCER1G, MME, GP1BB, HEG1, SFTPD, CCDC113, IL18, COMMD3, ANO6, HBA2, NME5, HBA1, KIF7, PALD1, LAT2, CLDN11, CLDN10, RAP2A, PITPNM1, SGK3, ALDOC, CD48, HNRNPH3, CRELD1, CRELD2, DNASE1L3, MATN2, ARHGEF6, TLR2, C1QA, TAGLN, MEGF6, AMIGO2, CRIP2, PTN, ZDHHC22, AIF1, CST6, CST3, PGRMC1, RXRA, TCEAL5, PNMA1, STAMBPL1, SHOX, GPC5, MSX1, APBB3, DYNC1I1, RFTN2, MPP1, CD74, GABBR1, IL34, YIPF5, AP3D1, RPA1, RPA2, LHFPL2, LYVE1, KAZALD1, YIF1A, ALDH4A1, SPCS2, FAP, TTC3, ESYT1, ACTR10, FCGBP, CD83, CD81, OGDHL, TTF2, CCDC28B, LOXL1, ACTG1, RGS5, TMEM147, MAGED4B, POGZ, CAPN2, COTL1, MAGEB10, PDGFRB, MORC4, SLC30A3, APLP2, MATK, EPDR1, SHISA4, PCOLCE, C2ORF15, ATP1B2, TMEM130, PDIA6, LZTFL1, SLFN12, DOK1, TYROBP, COL4A2, APLNR, SCG5, MATR3, MZF1, VCL, PPIC, RAI14, RTN1, CCL3L1, NDUFB10, HTRA1, LTBP2, LTBP3, FBLN5, RTN4, UBB, PODXL, CDH23, TBC1D14, COX11, MYH10, CD99, HSPA8,*

		TFAP2B, CBX7, CBX6, HBG2, TGFB3, IDH1, IFT20, COL5A1, DNAJC10, GNAS, XAGE2, DOCK6, LGALS3BP, COLGALT2, THRA, HSPB2, PSIP1, PKD2, SLC2A5, DMKN, RABEPK, PKD1, IFT52, LYL1, LRRFIP1, SCARF2, YWHAH, KLF13, ATP6AP1, ATP6AP2, ARAP3, POMGNT1, LSS, DCN, LMBRD1, GAP43, CREB1, FCHSD2, ACOX2, GORASP2, DOCK2, CHMP5, COLEC12, GALT, TM2D2, GMPR2, LCAT, LTC4S, FAM156A, CYTH4, GMDS, TRA2A, NCAM1, PDLIM5, MBTPS1, LUM, MX1, NAP1L1, TTC17, SOD1, POLE4, SPATA33, SCD, KANSL2, GLB1, ALOX5AP, CDK2AP1, VIM, TEK, SSBP4
Class II protein complex binding	2.47E-07	*HSPA8, CD74, HLA-DMA, HLA-DMB, CD81, HLA-DPB1, HLA-DRA, HLA-DQA1, HLA-DPA1, HLA-DQB1*
Extracellular matrix structural constituent	5.15E-07	*SPARC, LUM, FN1, PRELP, LTBP2, PCOLCE, COL3A1, EFEMP2, COL1A2, COL4A2, COL5A1, COL4A4, MGP, COL5A2, COL8A2, IGFBP7, CRELD1, MXRA5, CTHRC1, MATN2*
MHC class II receptor activity	2.90E-06	*HLA-DMA, HLA-DPB1, HLA-DRA, HLA-C, HLA-DQA1, HLA-DPA1, HLA-DQB1*
Oxygen transporter activity	1.23E-05	*HBG2, HBG1, HBB, HBA2, HBD, HBA1*
Haptoglobin binding	1.28E-05	*HBG2, HBG1, HBB, HBA2, HBD, HBA1*
Organic acid binding	2.27E-05	*HBG2, HBG1, HBB, HBA2, HBD, HBA1*
Beta-amyloid binding	2.50E-04	*CST3, C1QA, CD74, MARCO, PGRMC1, ITGAM, TREM2, GPRASP2, APBB3, ITM2B, ITM2C, TLR2*
Hemoglobin alpha binding	4.37E-04	*HBG2, HBG1, HBB, HBD*
Platelet-derived growth factor binding	4.45E-04	*PDGFRB, COL3A1, COL1A2, COL5A1, COL6A1*
Calcium ion binding	5.51E-04	*FBLN7, MEGF6, SPARC, FKBP14, LPCAT1, LTBP2, PLA2G5, LTBP3, PCDH12, SRI, PKD2, AIF1, FBLN5, THBD, EFEMP2, CDH2, CDH23, S100A13, CAPN2, SDF4, PLS3, PAMR1, GSN, HEG1, TNNC1, PCDH7, ANXA4, GLCE, ANXA5, EPDR1, SULF1, SULF2, NELL2, CLEC4A, MYL5, PITPNM1, SYT11, SMOC1, MGP, RGN, CRELD1, ESYT1, CRELD2, DNASE1L3, CD320, MATN2*
Extracellular matrix structural constituent conferring tensile strength	5.79E-04	*COL3A1, COL1A2, COL4A2, COL5A1, COL4A4, COL5A2, COL6A1, COL8A2*
Integrin binding	7.08E-04	*ITGAM, VCAM1, ITGB5, CD81, FN1, PTN, FBLN5, NISCH, COL3A1, SFRP2, COL5A1, FAP, TIMP2, ITGBL1, P4HB, JAM3*
Identical protein binding	0.001065	*AHCYL1, OLFML2B, PKD2, PIK3CG, FGFRL1, IFIT3, ACTG1, AQP1, C1QTNF3, TUBA1A, LGALS1, CDH2, DPYSL2, MPC2, SDF4, CCDC92, SKP2, TRIM22, YWHAH, CD53, FCER1G, GP1BB, TPM2, ANXA4, APLP2, SFTPD, EPDR1, DYNLL1, TERF1, LZTFL1, CLDN11, NISCH, CLDN10, TYROBP, CREB1, MAF, MATR3, KIFC3, NBL1, ALDH7A1, TLR2, BEX3, KHDRBS1, PRPS1, KHDRBS3, NLGN2, MAOB, MAGED1, PON2, SDC3, HTRA1, SRI, LTC4S, FHIT, CST3, LDHA, PRDX4, RXRA, GMDS, ESD, CCL3, IGFBP6, HNRNPA1, CD74, TGFB3, ST13, IL34, TNK2, IDH1, CARD9, MX1, FN1, CYBRD1, PAICS, SOD1, ALDH4A1, COL1A2, FAP, P4HA1, ZNF618, SYT11, VPS41, ALOX5AP, VIM, ESYT1, TEK, GSTM5*
Oxygen binding	0.001628	*HBG2, HBG1, HBB, HBA2, HBD, HBA1*
Heparin binding	0.001747	*FBLN7, APLP2, FN1, PRELP, LTBP2, PCOLCE, PTN, FGFRL1, NELL2, EFEMP2, CCL8, COL5A1, FGF9, MDK, SMOC1, RSPO2*
Peroxidase activity	0.001885	*PRDX4, HBG2, HBG1, HBB, HBA2, HBD, HBA1*
Extracellular matrix structural constituent	0.002124	*LUM, OGN, PRELP, FMOD, DCN*

conferring compression resistance		
G-rich strand telomeric DNA binding	0.004579	*RPA1*, *RPA2*, *TERF1*, *HNRNPA1*
Cobalamin binding	0.004579	*LMBRD1*, *TCN2*, *MMAB*, *CD320*
Symporter activity	0.005838	*SLC22A4*, *SLC24A3*, *SLC20A2*, *SLC2A10*, *SLC20A1*, *SLC4A11*, *SLC16A9*, *SLC15A3*, *SLC15A4*
Inorganic phosphate transmembrane transporter activity	0.007363	*ANKH*, *SLC20A2*, *SLC20A1*
Insulin-like growth factor binding	0.008471	*IGFBP2*, *HTRA1*, *IGFBP7*, *IGFBP6*, *KAZALD1*
Carboxypeptidase activity	0.008471	*CTSA*, *CPQ*, *CTSZ*, *CPE*, *PRCP*
Peptide antigen binding	0.009908	*HLA-DPB1*, *HLA-DRA*, *HLA-C*, *HLA-DQA1*, *HLA-DPA1*, *HLA-DQB1*

MHC, major histocompatibility complex.
