# Supplementary material for: Novel potential drugs for the treatment of primary open-angle glaucoma using protein-protein interaction network analysis
Source: Genomics Inform. 2023 Mar 31;21(1):e6. doi: 10.5808/gi.22070 (PMC10085733; doi:10.5808/gi.22070)
Supplement: Supplementary Table 4. — Upregulated genes based on cell component enrichment analysis [file gi-22070-Supplementary-Table-4.pdf]

Supplementary Table 4. Upregulated genes based on cell component enrichment analysis

Cell component	p-value	Genes
Extracellular region	3.25E-14	*SPARC, OLFML2B, CYB5D2, LOXL1, EFEMP2, ISLR, LGALS1, FGF9, COTL1, FAM3A, IL13RA2, PTGDS, PAMR1, IGFBP2, CLEC11A, HSD11B1L, EPDR1, RNASE4, PCOLCE, MPEG1, DKK3, SFRP2, COL4A2, COL4A4, COL8A2, SCG5, SERPING1, MXRA5, NBL1, VCL, LY6E, CEMIP, CCL3L1, HTRA1, LY96, C1ORF54, TNFRSF11B, LTBP2, PLA2G5, LTBP3, FBLN5, FUT6, C5, PRDX4, ANKH, PDGFD, S100A13, IGFBP7, IGFBP6, CD14, CTSA, HSPA8, TGFB3, TNFSF15, IDH1, FN1, PPBP, COL1A2, COL5A1, TCN2, TMEM98, COL5A2, OGN, PPT1, SCGB3A2, GNAS, ITGBL1, MAGED2, ITM2B, LGALS3BP, CPQ, CTSZ, HBB, TREM2, DMKN, TNFSF13B, GLT1D1, MDK, TIMP2, RSPO2, GUSB, CD52, HEG1, SFTPD, ANXA5, IL18, COMMD3, HBA2, NME5, HBA1, DCN, F5, COL6A1, TNFRSF25, ALDOC, DOCK2, DNASE1L3, MATN2, C1QA, ITIH3, MEGF6, GRP, PON2, LCAT, PRELP, SEMA3E, PTN, DPCD, COCH, CST3, OLFML3, FRZB, OLFML1, CCL3, NCAM1, GPC5, GPHA2, LEFTY2, CTHRC1, TUB, CA11, GABBR1, GSN, LUM, IL34, KAZALD1, SOD1, NELL2, COL3A1, GLB1, SMOC1, MGP, MNDA, TEK, P4HB, CCDC3, FMOD, ACTR10, KLKB1, CRLF1*
Extracellular exosome	1.55E-12	*FCGBP, AHCYL1, CD81, ENO1, HNMT, ACTG1, AQP1, EFEMP2, ISLR, LGALS1, SCN11A, DPYSL2, CAPN2, COTL1, ANKFY1, CPNE3, PTGDS, APLP2, IGFBP2, HLA-C, PCOLCE, PDIA6, COL4A2, MXRA8, SERPING1, PHPT1, MXRA5, HPD, ALDH7A1, VCL, PPIC, CRABP2, HTRA1, RHOBTB3, LTBP2, PCDH12, LTBP3, FBLN5, ACAT2, FUT6, LDHA, C5, PRDX4, UBB, PODXL, TSPAN6, ESD, IGFBP7, TSPAN3, CD14, MYH10, CTSA, HSPA8, VCAM1, ST13, IDH1, FN1, CYBRD1, NEB, PAICS, HNRNPM, EXT2, COL1A2, TMEM98, OGN, PPT1, GNAS, HLA-DRA, CPE, SERPINI1, COPS8, ITM2B, ITM2C, LGALS3BP, ARF4, ITGAM, ITGB5, MTMR11, CPQ, CTSZ, HBB, AKR1B1, SLC2A5, PKD2, PRSS23, PKD1, C1QTNF3, FCGR3A, TUBA1A, SDF4, CTSF, GUSB, YWHAH, CD53, ATP6AP1, SSR4, MME, ANXA4, GAA, ANXA5, ATP6AP2, ANO6, HBA2, HBA1, LAT2, BDH2, DDAH1, PSMA1, COL6A1, CD48, ALDOC, KIFC3, DOCK2, VAMP5, CHMP5, PCYOX1, ITIH3, SLC20A2, LCAT, PRCP, PRELP, SRI, CST6, CST3, GMDS, HNRNPA1, GPR155, CD74, GSN, LUM, LYVE1, SOD1, GLB1, MGP, PDCD2, MNDA, VIM, P4HB, KLKB1*
Extracellular space	2.00E-12	*FCGBP, SPARC, OLFML2B, ENO1, LOXL1, ACTG1, EFEMP2, LGALS1, FGF9, FCGRT, FAM3A, IL13RA2, HMGN2, PTGDS, SEMA6D, IGFBP2, CLEC11A, RNASE4, PCOLCE, VASH1, PDIA6, DKK3, SFRP2, COL4A2, COL4A4, COL8A2, SERPING1, NBL1, FBLN7, CCL3L1, MRPL18, HTRA1, LY96, TNFRSF11B, LTBP2, FBLN5, PCSK1N, C5, PRDX4, UBB, PODXL, PDGFD, S100A13, CYTL1, IGFBP7, ARSG, IGFBP6, CD14, HSPA8, VCAM1, TGFB3, TNFSF15, ABCA3, FN1, LY86, PPBP, COL1A2, COL5A1, TCN2, TMEM98, COL5A2, OGN, SCGB3A2, CPE, LRRN1, SERPINI1, ITM2B, LGALS3BP, ITGAM, CPXM2, CPQ, CTSZ, HBB, AKR1B1, DMKN, TNFSF13B, THBD, C1QTNF3, GLIPR1, FCGR3A, TCTN1, TIMP2, CTSF, GUSB, METRN, SFTPD, IL18, HBA2, HBA1, DCN, F5, COL6A1, LTB, CRELD2, COLEC12, GRP, LCAT, PRELP, SEMA3E, PTN, CST3, CCL8, OLFML3, FRZB, OLFML1, CCL3, GPC5, GPHA2, LEFTY2, CTHRC1, JAM3, GABBR1, GSN, LUM, IL34, SULF1, SULF2, SOD1, COL3A1, FAP, SMOC1, FMOD, IL17D, SCRG1, KLKB1, CRLF1*
Lysosomal lumen	4.45E-09	*CTSA, PDGFRB, CD74, HSPA8, LUM, GAA, SDC3, EPDR1, PRELP, DCN, GALC, TCN2, GLB1, OGN, PPT1, GPC5, CTSF, GUSB, FMOD*
Membrane	4.61E-09	*RERGL, SPARC, NCF1, RASL11B, CD81, NCF4, STMN2, ENO1, SLC4A3, CYB5D2, TMEM263, ACTG1, AQP1, FADS2, C3ORF18, APMAP, POFUT1, DPYSL2, ANKFY1, SLC16A9, VSIG4, IL13RA2, FADS1, RPS12, PDGFRB, ACVR1, SLC30A3, APLP2, MATK, HLA-C, SHISA4, ATP1B2, SIGIRR, NISCH, TYROBP, COL4A2, PRKAR1A, COL4A4, MATR3, TMEM126B,*

		MXRA7, LY6E, KHDRBS1, TMEM59L, SDC3, GLT8D2, CEND1, GABARAP, BCL2L13, PTDSS1, LDHA, TMEM245, ANKH, CDH23, CHST15, ST3GAL5, SLC15A3, SLC15A4, HLA-DQA1, CTSA, FZD1, HSPA8, PRRT2, VCAM1, SLC35E1, TNFSF15, TNK2, CYBRD1, LAYN, SLC4A11, B3GALT6, PAICS, XKR8, HNRNPM, EXT2, FIBP, P4HA1, CALY, UST, REEP2, DNAJC10, PPT1, GNAS, RNF182, LRRN1, MAGED2, SERINC1, SLC35E2B, EIF4G2, ITM2B, CD320, MAP3K12, LGALS3BP, ARF4, PIGT, MTMR11, LPCAT1, PIK3CD, TREM2, SLC2A5, PKD2, NAT14, PKD1, RERG, PIK3CG, TNFSF13B, MRC2, C1QTNF3, GLIPR1, SEC61G, TCTN1, SDF4, SCARF2, GUSB, GAL3ST3, CTNNBL1, CD52, ATP6AP1, MME, SLC2A10, GAA, ANXA5, ATP6AP2, ABHD14A, ANO6, HBA2, TMEM42, EMP3, HBA1, DYNLL1, POMGNT1, LSS, F5, RAP2A, LMBRD1, MRAS, PITPNM1, DAD1, GORASP2, COL6A1, CD48, CRELD1, DOCK2, HLA-DQB1, COLEC12, NLGN2, TM2D2, SLC20A2, SLC20A1, ATP10D, AMIGO2, TMEM160, PBXIP1, SRI, PTN, LTC4S, HLA-DMA, PGRMC1, FAM156A, STAMBPL1, FRZB, SC5D, NCAM1, ZNF106, GPC5, CYYR1, RPL15, HNRNPA1, PDLIM5, GPR155, RNF130, APBB3, MPP1, CA12, CD74, KIDINS220, MX1, AP3D1, NAP1L1, LHFPL2, LYVE1, ARCN1, DHRS7, PINK1, FAP, SCD, EIF3L, VPS41, ALOX5AP, HLA-DPB1, ESYT1, CYB561
Endoplasmic reticulum lumen	2.21E-08	COLGALT2, FKBP14, CTSZ, PRSS23, CST3, LGALS1, CDH2, PDGFD, ESD, IGFBP7, ARSG, MBTPS1, PTPRN2, ERAP2, WFS1, APLP2, FN1, PDIA6, F5, COL3A1, COL1A2, COL4A2, COL5A1, P4HA1, P4HA2, COL4A4, COL5A2, COL6A1, DNAJC10, COL8A2, MXRA8, SERPING1, P4HB
Collagen trimer	5.03E-07	COLEC12, C1QA, SFTPD, PCOLCE, MARCO, C1QTNF3, COL3A1, COL1A2, COL4A2, COL5A1, P4HA1, COL4A4, COL5A2, COL6A1, COL8A2, CTHRC1
Cell surface	8.78E-07	CSF1R, NLGN2, SPARC, ITGAM, ITGB5, SDC3, CTSZ, CD99L2, PTN, ENO1, PKD1, THBD, HLA-DMA, LGALS1, CDH2, APMAP, HYAL2, RSPO2, TIMP2, NCAM1, GPC5, HLA-DPA1, FZD1, CD53, PDGFRB, CD74, FCER1G, VCAM1, MME, TGFB3, SEMA6D, ANXA4, ISLR2, LAYN, HLA-C, ANO6, SULF1, SULF2, TYROBP, FAP, HLA-DPB1, MXRA8, ITGBL1, HLA-DRA, TEK, VAMP5, HCST, TLR2
MHC class II protein complex	1.63E-06	CD74, HLA-DMA, HLA-DMB, HLA-DPB1, HLA-DRA, HLA-C, HLA-DQA1, HLA-DPA1, HLA-DQB1
Extracellular matrix	3.54E-06	COLEC12, FBLN7, FCGBP, OLFML2B, LTBP2, PRELP, TNFRSF11B, FBLN5, EFEMP2, TIMP2, LUM, FN1, DCN, COL3A1, COL1A2, COL4A2, COL5A1, COL4A4, COL5A2, COL6A1, MGP, OGN, COL8A2, LRRN1, FMOD, MATN2
Hemoglobin complex	3.72E-06	HBG2, HBG1, HBB, HBA2, HBD, HBA1
Golgi apparatus	6.21E-06	ARF4, NCF1, CPQ, LPCAT1, STMN2, PKD2, PKD1, FGFRL1, LAPTM4A, POFUT2, RUSC1, CAPN2, SDF4, KIF1C, PTGDS, TRIM22, RPS12, TMED4, PDGFRB, PAQR8, CHST6, SEMA6D, GLCE, HLA-C, TMEM130, ZDHHC14, F5, GORASP2, FEZ1, KIFC3, CRELD2, VAMP5, HLA-DQB1, TLR2, GALT, GLT8D2, ZDHHC22, PLA2G5, GABARAP, CST3, FUT6, FGD6, ST3GAL5, TBC1D14, IGFBP6, CD14, JAM3, B3GALNT1, MBTPS1, VCAM1, SLC35E1, YIPF5, AP3D1, B3GALT6, SULF1, YIF1A, IFT20, EXT2, GLB1, VPS41, TTC3, PPT1, CPE, VIPAS39, PSMG1, SCRG1, SLC35E2B, ITM2B, ITM2C
Haptoglobin-hemoglobin complex	1.80E-05	HBG2, HBG1, HBB, HBA2, HBD, HBA1
Ficolin-1-rich granule lumen	2.23E-05	HSPA8, GSN, IDH1, CTSZ, HBB, COMMD3, CST3, PRDX4, GLB1, TIMP2, COTL1, ALDOC, MNDA, GUSB, ACTR10, VCL
Endoplasmic reticulum membrane	2.76E-05	ZNF177, AHCYL1, PIGT, PIGP, LPCAT1, PKD2, FADS2, POFUT2, TMEM147, SEC61G, PTGDS, FADS1, HLA-DPA1, TMED4, VKORC1, ATP6AP1, SSR4, SFTPD, ATP6AP2, COPZ2, HLA-C, PDIA6, LSS, ZDHHC14, PYURF, LMBRD1, PITPNM1, GORASP2, DAD1, KDELR3, HPD, HLA-DQB1, RTN1, ATP10D, ZDHHC22, SRI, LTC4S, RTN4, DNAJB2, PTDSS1, UBB, SC5D, SARAF, HLA-DQA1, MBTPS1, CD74, GABBR1, ERAP2, WFS1, YIPF5, MX1, YIF1A, ARCN1, EXT2, DHRS7, SPCS2,

		TMEM98, SCD, REEP2, ALOX5AP, HLA-DPB1, TMEM119, HLA-DRA, SERINC1, ESYT1
Integral component of lumenal side of endoplasmic reticulum membrane	4.30E-05	*CD74, HLA-DPB1, HLA-DRA, HLA-C, PKD2, HLA-DQA1, HLA-DPA1, HLA-DQB1*
Integral component of membrane	6.74E-05	*ZNF177, ANKLE1, SLC4A3, FGFRL1, AQP1, C3ORF18, VSIG4, MPV17, SLC35A5, ACVR1, PAQR8, GLCE, HLA-C, ISLR2, SIGIRR, WDR82, MXRA8, VOPP1, TMEM126B, MXRA7, KDELR3, SLC22A4, IGSF6, MAOB, SDC3, CEND1, CD99L2, PCDH12, PTDSS1, LDHA, ANKH, TSPAN6, CHST15, ST3GAL5, TSPAN3, HLA-DQA1, FZD1, B3GALNT1, PTPRN2, PRRT2, SLC35E1, TNFSF15, ABCA3, TMEM190, RMDN3, XKR8, EXT2, MARCO, TMEM98, CALY, LRRN1, ITM2B, ITM2C, ARF4, PIGP, LPCAT1, HBB, HBD, NAT14, MRC2, GLIPR1, SLC22A17, SEC61G, HLA-DPA1, TMED4, VKORC1, CD53, CD52, CHST6, FCER1G, SSR4, MME, SLC2A10, GP1BB, HEG1, GAA, ANO6, TMEM42, EMP3, ZDHHC14, LAT2, CLDN11, MS4A6A, CLDN10, PSMA1, CD48, CRELD1, HCST, HLA-DQB1, TLR2, SLC20A2, SLC20A1, AMIGO2, TMEM160, ZDHHC22, HLA-DMA, PGRMC1, HLA-DMB, SLC17A9, SC5D, GPC5, GPR155, RNF130, CA12, CD74, GABBR1, TIMMDC1, KIDINS220, YIPF5, LHFPL2, LYVE1, YIF1A, SPCS2, FAP, HLA-DPB1, ESYT1, CYB561, CD83, CHPF, TMEM179B, CD81, TMEM263, LAMP5, LAPTM4A, TMEM147, APMAP, CDH2, FCGRT, MPC1, ADORA3, MPC2, ADORA1, FAM3A, SLC16A9, IL13RA2, SLC25A45, SLC25A44, FADS1, PDGFRB, SLC30A3, SEMA6D, APLP2, SHISA4, TMEM130, LZTFL1, MPEG1, CLEC4A, ARMCX2, TYROBP, APLNR, FAM8A1, CSF1R, RTN1, TMEM59L, GLT8D2, MCHR1, RTN4, FUT6, BCL2L13, TMEM245, HCAR1, PODXL, CDH23, COX11, ZNF546, SMYD3, SLC15A3, SLC15A4, CD99, VCAM1, WFS1, CYBRD1, LAYN, SLC4A11, B3GALT6, TMEM17, REEP2, DNAJC10, UST, GNAS, TMEM119, RNF182, HLA-DRA, SERINC1, SLC35E2B, ITGAM, MEGF10, TREM2, PKD2, SLC2A5, ATRAID, PKD1, TNFSF13B, FCGR3A, HEPH, NPIPA1, SCARF2, GAL3ST3, ATP6AP1, ATP6AP2, KCNAB1, ABHD14A, POMGNT1, LMBRD1, DAD1, TNFRSF25, LTB, COLEC12, NFAT5, PRPS1, WBP1, SMPX, TM2D2, NDUFB5, PON2, ATP10D, LCAT, PBXIP1, LTC4S, FAM156A, TMEM204, NCAM1, CYYR1, MBTPS1, ERAP2, PCDH7, NELL2, SCD, ALOX5AP, SSBP4*
Endoplasmic reticulum	7.56E-05	*CPQ, PIGP, CTSZ, LPCAT1, PKD2, PKD1, IFIT2, POFUT2, POFUT1, HYAL2, ADORA1, CAPN2, SDF4, KIF1C, CTSF, TMED4, VKORC1, SSR4, HLA-C, VASH1, PDIA6, ZDHHC14, KDELR3, MXRA7, CRELD2, DNASE1L3, NDUFB8, CEMIP, RTN1, CRABP2, ATP10D, PTN, ZDHHC22, LTC4S, RTN4, CST3, PGRMC1, PRDX4, SARAF, ARSG, CTSA, VCAM1, ERAP2, WFS1, YIPF5, SULF1, SULF2, ARCN1, EXT2, PINK1, COL1A2, TMEM98, P4HA1, P4HA2, SCD, APEX1, DNAJC10, OGN, REEP2, ALOX5AP, PSMG1, P4HB, ESYT1, CCDC3, CD320*
Endosome membrane	7.94E-05	*ATP6AP1, ATP6AP2, NCF4, AP3D1, LY96, RABEPK, LAMP5, HLA-DMA, HLA-DMB, UBB, FCGRT, VPS41, HLA-DPB1, HLA-DRA, ANKFY1, CD14, SLC15A3, ITM2B, HLA-DQA1, HLA-DPA1, HLA-DQB1, CHMP5, CD320*
Lysosomal membrane	1.11E-04	*ATRAID, LAMP5, LAPTM4A, HLA-DMA, HLA-DMB, ANKFY1, SLC15A3, SLC15A4, HLA-DQA1, HLA-DPA1, CTSA, CD74, HSPA8, SLC30A3, ABCA3, GAA, ATP6AP2, AP3D1, CYBRD1, LMBRD1, SYT11, VPS41, COL6A1, HLA-DPB1, HLA-DRA, CYB561, ITM2C, HLA-DQB1, CHMP5*
Plasma membrane	1.55E-04	*SPARC, ENO1, SLC4A3, FGFRL1, AQP1, SCN11A, DPYSL2, PLCE1, CPNE3, ACVR1, PAQR8, HLA-C, KCNK15, ISLR2, SIGIRR, NISCH, PRKAR1A, FEZ1, MXRA8, SLC22A4, IGSF6, MAGED1, SDC3, CD99L2, PCDH12, PLA2G5, GABARAP, ANKH, KCNMB1, TSPAN6, S100A13, ST3GAL5, TSPAN3, CD14, HLA-DQA1, FZD1, PTPRN2, PRRT2, TNFSF15, ABCA3, MICAL3, FN1, XKR8, MARCO, TCN2, TMEM98, CALY, TRIP6, ITGBL1, ITM2B, CD320, ITM2C, ARF4, CTSZ, LPCAT1, GLIPR1, TUBA1A, SLC22A17, HYAL2, SDF4, CTSF, HLA-DPA1, CD53, CD52, FCER1G, MME,*

		SLC2A10, GP1BB, ANXA4, GAA, ANO6, EMP3, DYNLL1, F5, LAT2, CLDN11, MS4A6A, CLDN10, RAP2A, MRAS, CD48, VAMP5, HCST, HLA-DQB1, PCYOX1, TLR2, SLC20A2, SLC20A1, AMIGO2, PTN, ZDHHC22, CST3, PGRMC1, GPC5, JAM3, RFTN2, MPP1, CA12, CD74, GABBR1, GSN, CARD9, LHFPL2, SULF1, LYVE1, WDR13, SULF2, FAP, HLA-DPB1, RIMBP2, ESYT1, KLKB1, CD83, NCF1, TMEM179B, CD81, ACTG1, LAMP5, RGS4, FADS2, RGS5, TMEM147, CDH2, ADORA3, ADORA1, CAPN2, COTL1, PLS3, SLC16A9, PDGFRB, SLC30A3, SEMA6D, APLP2, ATP1B2, TMEM130, PDIA6, CLEC4A, TYROBP, APLNR, PHPT1, VCL, LY6E, SHC4, CSF1R, CEMIP, HTRA1, RHOBTB3, LY96, TNFRSF11B, FHIT, MCHR1, RTN4, HCAR1, ADAP2, UBB, PODXL, CDH23, SLC15A4, CD99, HSPA8, VCAM1, TGFB3, TNK2, CYBRD1, SLC4A11, COPRS, REEP2, GNAS, TMEM119, HLA-DRA, CPE, SERINC1, MAP3K12, ITGAM, ITGB5, MEGF10, PIK3CD, TREM2, PKD2, SLC2A5, ATRAID, PKD1, RERG, PIK3CG, TNFSF13B, TRO, THBD, FCGR3A, HEPH, LRRFIP1, YWHAH, ATP6AP2, ARAP3, KCNAB1, GAP43, LMBRD1, FCHSD2, TNFRSF25, LTB, DOCK2, CHMP5, COLEC12, SNAP47, SLC24A3, NLGN2, RNF34, PON2, ATP10D, PRCP, SEMA3E, CYTH4, TMEM204, NCAM1, CACNG3, SH2B3, TUB, ERAP2, PCDH7, MX1, TTC17, SOD1, KANSL2, SYT11, VIM, TEK
Golgi membrane	2.49E-04	*RTN1, CHPF, TMEM59L, LPCAT1, ZDHHC22, PKD1, GABARAP, FUT6, CYTH4, HYAL2, PDGFD, CHST15, ST3GAL5, PLCE1, NCAM1, SLC35A5, HLA-DQA1, HLA-DPA1, CSGALNACT1, B3GALNT1, CD74, MBTPS1, CHST6, GLCE, AP3D1, COPZ2, HLA-C, B3GALT6, POMGNT1, TMEM130, YIF1A, ARCN1, IFT20, EXT2, GORASP2, UST, HLA-DPB1, HLA-DRA, KDELR3, HPD, ITM2B, HLA-DQB1*
Transport vesicle membrane	4.34E-04	*CD74, PTPRN2, HLA-DPB1, HLA-DRA, CPE, HLA-DQA1, HLA-DPA1, HLA-DQB1*
Lysosome	4.98E-04	*CTSA, CD74, HSPA8, CPQ, PON2, GAA, SFTPD, ATP6AP2, CTSZ, EPDR1, PRCP, GABARAP, SOD1, GALC, SYT11, HYAL2, VPS41, PPT1, CAPN2, HLA-DRA, CTSF, ARSG, ITM2C, PCYOX1*
Endocytic vesicle membrane	6.82E-04	*COLEC12, CD74, MARCO, UBB, HLA-DPB1, HLA-DRA, CACNG3, HLA-DQA1, HLA-DPA1, HLA-DQB1*
Basement membrane	0.001519	*EFEMP2, SPARC, COL4A2, COL5A1, FGF9, COL4A4, SMOC1, COL8A2, FN1, PTN, LOXL1*
Focal adhesion	0.00161	*FBLN7, ITGB5, CD81, CD99L2, ACTG1, TNFSF13B, MRC2, CDH2, CAPN2, CPNE3, SCARF2, TNS3, CD99, FZD1, PDGFRB, HSPA8, GSN, MME, ANXA5, LAYN, REXO2, FAP, TRIP6, ITGBL1, VIM, TEK, P4HB, VCL*
Intrinsic component of plasma membrane	0.001953	*TRO, PDGFRB, CD52, LY96, TREM2, CD48, TLR2*
Platelet alpha granule lumen	0.001982	*ISLR, SPARC, TGFB3, FN1, SERPING1, MAGED2, PPBP, LEFTY2, F5*
Endocytic vesicle lumen	0.002832	*SPARC, SCGB3A2, HBB, HBA2, HBA1*
Integral component of organelle membrane	0.003995	*SLC22A17, VOPP1, VAMP5, ITM2B*
Intracellular membrane-bounded organelle	0.005251	*AHCYL1, SPARC, CPQ, CTSZ, ATRAID, TNFSF13B, RGS5, ANKFY1, CTSF, GUSB, CCDC92, FADS1, HLA-DPA1, RPS12, PDGFRB, GAA, HSD11B1L, NISCH, LMBRD1, PITPNM1, ACOX2, SGK3, PPIC, CSF1R, RHOBTB3, PRCP, LTC4S, HLA-DMA, HLA-DMB, ADAP2, PODXL, TRA2A, SHOX, SC5D, TBC1D14, SLC15A3, ZNF540, CTSA, TGFB3, ABCA3, TNK2, YIPF5, YIF1A, SNX19, P4HA1, P4HA2, GLB1, PPT1, MNDA, ITM2B*

MHC, major histocompatibility complex.
