# Supplementary material for: Novel potential drugs for the treatment of primary open-angle glaucoma using protein-protein interaction network analysis
Source: Genomics Inform. 2023 Mar 31;21(1):e6. doi: 10.5808/gi.22070 (PMC10085733; doi:10.5808/gi.22070)
Supplement: Supplementary Table 5. — Upregulated genes based on biological process enrichment analysis [file gi-22070-Supplementary-Table-5.pdf]

Supplementary Table 5. Upregulated genes based on biological process enrichment analysis

Biological process	p-value	Genes
Antigen processing and presentation of exogenous peptide antigen via MHC class II	6.69E-07	*CD74, HLA-DMA, HLA-DMB, FCER1G, HLA-DPB1, HLA-DRA, CTSF, HLA-DQA1, HLA-DPA1, HLA-DQB1*
Carbon dioxide transport	3.91E-06	*HBG2, HBG1, HBB, HBA2, HBD, HBA1, AQP1*
Peptide antigen assembly with MHC class II protein complex	9.82E-06	*HLA-DMA, HLA-DMB, HLA-DPB1, HLA-DRA, HLA-DQA1, HLA-DPA1, HLA-DQB1*
Immunoglobulin production involved in immunoglobulin mediated immune response	1.47E-05	*HLA-DMA, HLA-DMB, HLA-DPB1, HLA-DRA, HLA-DQA1, HLA-DPA1, HLA-DQB1*
Collagen fibril organization	3.54E-05	*COL3A1, COLGALT2, SFRP2, COL1A2, COL5A1, P4HA1, LUM, COL5A2, VIPAS39, FMOD, LOXL1*
Antigen processing and presentation of peptide or polysaccharide antigen via MHC class II	4.22E-05	*HLA-DMA, HLA-DMB, HLA-DPB1, HLA-DRA, HLA-DQA1, HLA-DPA1, HLA-DQB1*
Mast cell degranulation	1.01E-04	*LAT2, GRP, SCN11A, S100A13, PIK3CD, PTGDS, PIK3CG*
Oxygen transport	1.53E-04	*HBG2, HBG1, HBB, HBA2, HBD, HBA1*
Antigen processing and presentation	1.84E-04	*CD74, HLA-DMA, HLA-DMB, HLA-DPB1, HLA-DRA, HLA-C, HLA-DQA1, HLA-DPA1, HLA-DQB1*
Hydrogen peroxide catabolic process	3.24E-04	*PRDX4, HBG2, HBG1, HBB, HBA2, HBD, HBA1*
Nitric oxide transport	3.88E-04	*HBB, HBA2, HBA1, AQP1*
Heart development	6.15E-04	*ACVR1, SPARC, HEG1, FN1, FOXJ1, KCNAB1, PTN, SRI, PKD2, PKD1, COL3A1, ADAP2, POFUT1, APLNR, ID3, PLCE1, TEK, PDLIM5, EIF4G2*
Positive regulation of T cell activation	8.24E-04	*HLA-DMA, HLA-DMB, HLA-DPB1, HLA-DRA, HLA-DQA1, HLA-DPA1, HLA-DQB1*
Cell-matrix adhesion	0.001169	*COL3A1, VCAM1, ITGAM, ITGB5, FN1, EPDR1, ITGBL1, PKD1, LYVE1, FBLN5, VCL, JAM3*
Aging	0.00123	*PDGFRB, C1QA, VCAM1, GSN, MME, TGFB3, IGFBP2, DCN, SOD1, CREB1, COL4A2, KCNMB1, APEX1, TIMP2, RGN, SERPING1, FADS1*
Skin development	0.001774	*TFAP2B, COL3A1, COL5A1, SLC2A10, COL5A2, LTB, PKD1*
Response to glucocorticoid	0.002087	*SPARC, MDK, ABCA3, IGFBP2, LCAT, PTGDS, HNMT, AIF1, PPARGC1B*
Positive regulation of phosphatidylinositol 3-kinase signaling	0.002217	*PDGFRB, NCF1, PDGFD, IL18, FN1, TREM2, SEMA3E, TEK, HCST, DCN*
Positive regulation of bone mineralization	0.003388	*ACVR1, RXRA, TGFB3, TMEM119, ANO6, PTN, ATRAID*
Glomerular basement membrane development	0.004091	*COL4A4, MPV17, SULF1, SULF2*
Positive regulation of chemokine production	0.004272	*CSF1R, CD74, C5, CARD9, IL18, AIF1, TLR2*

Cellular oxidant detoxification	0.004991	*PRDX4, HBG2, HBG1, ALOX5AP, HBB, HBA2, HBD, HBA1, LTC4S*
Positive regulation of cell death	0.005314	*ACOX2, HBG2, HBG1, HBB, HBA2, HBD, HBA1*
Platelet aggregation	0.005314	*FIBP, GNAS, FN1, HBB, VCL, PIK3CG, ACTG1*
Peptidyl-proline hydroxylation to 4-hydroxy-L-proline	0.005482	*PRDX4, P4HA1, P4HA2, P4HB*
Cell adhesion	0.006044	*FBLN7, LGALS3BP, NLGN2, ITGAM, ITGB5, AMIGO2, CD99L2, PBXIP1, PCDH12, ISLR, NUAK1, CDH2, PODXL, NCAM1, IGFBP7, MYH10, JAM3, VCAM1, GP1BB, PCDH7, FN1, LYVE1, CLDN11, CLEC4A, CLDN10, COL5A1, FAP, FEZ1, COL6A1, MXRA8, ITGBL1, VCL*
Response to drug	0.006557	*FZD1, TFAP2B, MAOB, ABCA3, CARD9, IGFBP2, TNFRSF11B, PTN, TERF1, SOD1, LDHA, CREB1, LGALS1, SFRP2, MDK, DAD1, SCN11A, APEX1, DPYSL2, TIMP2*
Positive regulation of smooth muscle cell chemotaxis	0.006809	*MDK, PDGFD, AIF1*
Purine nucleotide metabolic process	0.006809	*GUK1, GMPR2, FHIT*
Angiogenesis	0.006992	*PDGFRB, ACVR1, IL18, FN1, UBP1, VASH1, PIK3CG, ACTG1, NELL2, COL4A2, HEY1, FGF9, FAP, POFUT1, COL8A2, APLNR, TEK, JAM3*
Glycosaminoglycan catabolic process	0.007124	*GLB1, HYAL2, GUSB, LYVE1*
Elastic fiber assembly	0.007124	*COL3A1, EFEMP2, LTBP3, FBLN5*
Cellular response to interferon gamma	0.007199	*CCL8, GSN, CCL3L1, CCL3, VIM, AIF1, ACTG1, HLA-DPA1, TLR2, HLA-DQB1*
Positive regulation of phagocytosis	0.007211	*TUB, FCER1G, SFTPD, TREM2, PLA2G5, DOCK2, SOD1*
Ossification	0.008993	*EXT2, SPARC, THRA, MGP, CLEC11A, COL5A2, TMEM119, KAZALD1, PPARGC1B*
Kidney development	0.009148	*IFT20, TFAP2B, MME, WFS1, HYAL2, ALDH1A2, RGN, SULF1, PKD1, DCN, SULF2*
Response to axon injury	0.009423	*ARF4, LGALS1, TYROBP, TREM2, AIF1, SOD1*
Response to hydrogen peroxide	0.009555	*PDGFRB, LDHA, CAPN2, HBB, HBA2, HBA1, SOD1*
Tissue regeneration	0.009975	*GAP43, GSN, MDK, CPQ, PTN*

MHC, major histocompatibility complex.
