# Supplementary material for: Novel potential drugs for the treatment of primary open-angle glaucoma using protein-protein interaction network analysis
Source: Genomics Inform. 2023 Mar 31;21(1):e6. doi: 10.5808/gi.22070 (PMC10085733; doi:10.5808/gi.22070)
Supplement: Supplementary Table 6. — Downregulated genes based on Reactome pathway enrichment analysis [file gi-22070-Supplementary-Table-6.pdf]

Supplementary Table 6. Downregulated genes based on Reactome pathway enrichment analysis

Reactome pathway	p-value	Genes
Signal transduction	2.29E-06	*HDAC11, UBE2D3, RBPJ, CDC14B, PPP2R1A, MYB, RNF43, RALBP1, USP7, STARD8, ACTN1, CSNK1E, RUNX3, TUBA4A, MTNR1A, OBSCN, ADCY9, SPINT1, DAAM1, PSME4, RRAGD, PSME1, TRIB3, TRIB1, S100A9, SQSTM1, S100A8, ABCG1, NOTCH1, SHMT2, MAPKAP1, PXN, FPR1, DTX2, RASAL1, GATA3, SLC1A5, IQGAP1, HIF1A, STK3, C3, HCAR3, KLC3, STK38, EIF4EBP1, MYH14, MYH11, STAP2, SFN, RHPN2, SPTBN2, ABCA1, BRAP, PHC2, ZRANB1, POU2F1, JAG1, SMAD3, CGN, DRAP1, NFKBIA, DIAPH2, MKRN1, MUC20, ALK, FLT4, LAMC2, CXCL1, ADM, PCSK5, AREG, RND3, IKBKB, MECOM, DBT, EFHD2, FLOT1, FLOT2, RAC1, IFT57, HRAS, CTSD, DUSP4, VAV3, DUSP5, FGFBP1, DUSP3, EDN2, FGFBP2, VWF, DST, DUSP1, SPHK1, ARHGEF16, MMP3, F3, TNFRSF1A, CNKSR1, ARHGAP10, ELF3, ESRP2, RARA, RHOU, TFF1, ATP6V1B1, BIRC3, PPP1R15A, LAMA5, CCL13, COL11A1, ADH1A, GNAI3, HDAC8, ACVR1B, PPP2CA, CDC42, GNA15, PGRMC2, SH3BP1, PSMB3, MFN2, POLR2J, CD55, CDKN2B, LAMB3, NDE1, LIMK2, WWP1, DHRS3, MAPK13, FABP5, FABP6, PSMC1, DLC1, SPRY2, ACKR1, LGR4*
Signaling by interleukins	4.99E-06	*LAMA5, CSF3, IL1RN, CEBPD, MAOA, FPR1, IL20RB, RORA, CXCL1, GATA3, HIF1A, PPP2CA, IKBKB, CDC42, MUC1, PPP2R1A, PSMB3, PIM1, S100A12, DUSP4, POU2F1, DUSP3, SMAD3, MMP1, IL1R1, MMP3, TALDO1, TNFRSF1A, NFKBIA, BCL6, PSMC1, PSME4, PSME1, LCN2, PDCD4, RHOU, UBE2V1, ALPK1, SQSTM1*
Formation of the cornified envelope	2.27E-05	*KRT4, KRT13, KRT24, KRT5, EVPL, PPL, KLK12, TGM1, KRT19, KRT18, KRT17, PERP, KRT15, KRT14, PKP3, PRSS8, CAPN1*
Type I hemidesmosome assembly	2.57E-05	*COL17A1, LAMB3, DST, KRT14, LAMC2, KRT5*
Immune system	5.66E-05	*FCN3, CSF3, IL1RN, UBE2D3, HP, DEFB1, UBE3A, RORA, MT2A, TRIM29, PPP2R1A, TOM1, PIM1, CAPN1, IL1R1, TALDO1, FBXO11, TUBA4A, SLPI, PSME4, PSME1, TRIB3, MASP1, UBE2V1, ALPK1, S100A9, SQSTM1, S100A8, COL17A1, CFD, MAOA, MAPKAP1, FPR1, IL20RB, GATA3, IQGAP1, HIF1A, PPL, C3, KLC3, S100A12, AP1M2, S100A11, SPTBN2, VASP, POU2F1, SMAD3, KLHL25, XRCC5, ZBTB16, MYO5A, DERA, NFKBIA, BCL6, MKRN1, LCN2, EIF4G3, MUC20, MUC21, SERPINA3, PIGR, TNFAIP6, CXCL1, SLC2A3, IKBKB, NAPRT, RAC1, HRAS, CTSD, DUSP4, EDARADD, VAV3, SERPINB1, DUSP3, TRIM63, MMP1, MMP3, TMC6, TNFRSF1A, TAPBP, CEACAM1, CEACAM6, IRF1, RHOU, CD44, ATP6V1B1, BIRC3, LAMA5, CEBPD, AGPAT2, LILRA5, PPP2CA, CDC42, MUC1, PSMB3, CD55, MAP3K1, MYO10, WWP1, MAPK13, LRG1, CLEC2B, FABP5, PSMC1, PDCD4, RAB18, S100P*
Extracellular matrix organization	1.22E-04	*COL17A1, LAMA5, TNXB, SDC4, COL11A1, LAMC2, FBLN2, ADAMTS4, CAPNS2, DMD, CAPN1, TPSAB1, CTSD, ADAMTS9, CAST, LAMB3, VWF, DST, MMP1, ACTN1, MMP3, BMP7, CEACAM1, BMP1, CEACAM6, CD44*
Cytokine signaling in immune system	1.23E-04	*LAMA5, CSF3, IL1RN, CEBPD, MAOA, UBE2D3, FPR1, IL20RB, RORA, CXCL1, GATA3, HIF1A, PPP2CA, IKBKB, CDC42, MT2A, MUC1, PPP2R1A, TRIM29, PSMB3, PIM1, S100A12, HRAS, EDARADD, DUSP4, POU2F1, DUSP3, SMAD3, MMP1, IL1R1, MMP3, TALDO1, TNFRSF1A, NFKBIA, BCL6, PSMC1, IRF1, PSME4, PSME1, LCN2, PDCD4, RHOU, UBE2V1, ALPK1, EIF4G3, SQSTM1, CD44, BIRC3*
Interleukin-4 and interleukin-13 signaling	1.66E-04	*LAMA5, POU2F1, CEBPD, MAOA, MMP1, MMP3, RORA, GATA3, HIF1A, MUC1, BCL6, PIM1, LCN2, RHOU*
Degradation of the extracellular matrix	2.00E-04	*COL17A1, CAST, LAMA5, LAMB3, MMP1, COL11A1, MMP3, LAMC2, ADAMTS4, BMP1, CAPNS2, CAPN1, TPSAB1, CTSD, ADAMTS9, CD44*
Innate immune system	3.50E-04	*SERPINA3, PIGR, FCN3, TNFAIP6, UBE2D3, HP, DEFB1, CXCL1, SLC2A3, IKBKB, PPP2R1A, NAPRT, TOM1, CAPN1, RAC1, HRAS, CTSD, DUSP4, VAV3, SERPINB1, DUSP3, TMC6, CEACAM1, SLPI, CEACAM6, PSME4, PSME1, MASP1,*

		UBE2V1, ALPK1, S100A9, S100A8, CD44, ATP6V1B1, BIRC3, CFD, FPR1, IQGAP1, AGPAT2, PPP2CA, C3, CDC42, MUC1, PSMB3, S100A12, S100A11, CD55, MAP3K1, MYO10, XRCC5, MYO5A, MAPK13, DERA, NFKBIA, LRG1, FABP5, PSMC1, RAB18, LCN2, S100P, MUC20, MUC21
Neutrophil degranulation	4.53E-04	*CFD, SERPINA3, PIGR, TNFAIP6, HP, FPR1, CXCL1, SLC2A3, IQGAP1, AGPAT2, C3, NAPRT, TOM1, S100A12, RAC1, CAPN1, CTSD, CD55, S100A11, SERPINB1, XRCC5, TMC6, DERA, CEACAM1, LRG1, FABP5, SLPI, CEACAM6, RAB18, LCN2, S100P, S100A9, CD44, S100A8*
Cell junction organization	5.02E-04	*VASP, COL17A1, LAMB3, DST, ACTN1, PXN, KRT14, CLDN7, LAMC2, KRT5, FLNC, CLDN1*
TP53 regulates transcription of several additional cell death genes whose specific roles in p53-dependent apoptosis remain uncertain	0.001363	*PPP1R13B, BCL6, PERP, RABGGTA, NDRG1*
Signaling by receptor tyrosine kinases	0.001879	*ALK, LAMA5, MAPKAP1, FLT4, COL11A1, PXN, LAMC2, PCSK5, HIF1A, AREG, PPP2CA, CDC42, PPP2R1A, RAC1, HRAS, CTSD, POLR2J, VAV3, DUSP4, FGFBP1, FGFBP2, DUSP3, LAMB3, SPHK1, WWP1, F3, MAPK13, SPINT1, ESRP2, SPRY2, TRIB3, TRIB1, MUC20, ATP6V1B1*
Cell-cell communication	0.002887	*VASP, COL17A1, LAMB3, DST, ACTN1, PXN, LAMC2, KRT5, IQGAP1, CLDN1, KRT14, CLDN7, FLNC*
TLR4 cascade	0.00289	*DUSP4, DUSP3, MAP3K1, UBE2D3, PPP2CA, NFKBIA, IKBKB, PPP2R1A, S100A12, UBE2V1, ALPK1, S100A9, S100A8, BIRC3*
MyD88:MAL (TIRAP) cascade initiated on plasma membrane	0.002996	*DUSP4, NFKBIA, PPP2CA, IKBKB, DUSP3, MAP3K1, PPP2R1A, S100A12, UBE2V1, ALPK1, S100A9, S100A8*
TLR6:TLR2 cascade	0.002996	*DUSP4, NFKBIA, PPP2CA, IKBKB, DUSP3, MAP3K1, PPP2R1A, S100A12, UBE2V1, ALPK1, S100A9, S100A8*
Interleukin-1 signaling	0.003435	*NFKBIA, IKBKB, IL1RN, IL1R1, PSMB3, PSMC1, PSME4, PSME1, S100A12, UBE2V1, ALPK1, SQSTM1*
TLR2 cascade	0.003672	*DUSP4, NFKBIA, PPP2CA, IKBKB, DUSP3, MAP3K1, PPP2R1A, S100A12, UBE2V1, ALPK1, S100A9, S100A8*
TLR1:TLR2 cascade	0.003672	*DUSP4, NFKBIA, PPP2CA, IKBKB, DUSP3, MAP3K1, PPP2R1A, S100A12, UBE2V1, ALPK1, S100A9, S100A8*
Negative regulation of MAPK pathway	0.004668	*DUSP4, BRAP, PPP2CA, DUSP5, DUSP1, PPP2R1A, HRAS*
TLR3 cascade	0.005332	*DUSP4, NFKBIA, PPP2CA, IKBKB, DUSP3, PPP2R1A, UBE2D3, S100A12, UBE2V1, ALPK1, BIRC3*
Keratinization	0.006064	*KRT4, KRT13, KRT24, KRT5, EVPL, PPL, KLK12, TGM1, KRT19, KRT18, KRT17, PERP, KRT15, KRT14, PKP3, PRSS8, CAPN1*
Metallothioneins bind metals	0.0066	*MT2A, MT1A, MT1M, MT1X*
MyD88-independent TLR4 cascade	0.007373	*DUSP4, NFKBIA, PPP2CA, IKBKB, DUSP3, PPP2R1A, UBE2D3, S100A12, UBE2V1, ALPK1, BIRC3*
TRIF(TICAM1)-mediated TLR4 signaling	0.007373	*DUSP4, NFKBIA, PPP2CA, IKBKB, DUSP3, PPP2R1A, UBE2D3, S100A12, UBE2V1, ALPK1, BIRC3*
Developmental biology	0.00861	*RPL32, KRT24, ADIRF, MED16, RBPJ, TGM1, MYB, PRSS8, CAPN1, RAC1, HRAS, VAV3, KRT4, RPS5, PAX6, KRT5, MED4, TUBA4A, RGMA, MED25, PSME4, RARA, PSME1, PKP3, EZR, NOTCH1, CEBPD, ACVR1B, EVPL, PPL, CDC42, RELN, PSMB3, FAM120B, PERP, ZSWIM8, MYH14, MYH11, POLR2J, SPTBN2, VASP, SMAD3, MYO10, LGI4, LIMK2, KRT13, KLF4, KLK12, MAPK13, EFNA1, DRAP1, KRT19, MEIS1, KRT18, FABP4, KRT17, KRT15, PSMC1, KRT14*
TLR cascades	0.009341	*DUSP4, DUSP3, MAP3K1, UBE2D3, PPP2CA, NFKBIA, IKBKB, PPP2R1A, S100A12, UBE2V1, ALPK1, S100A9, S100A8, BIRC3*

MyD88 cascade initiated on plasma membrane	0.009472	*DUSP4*, *NFKBIA*, *PPP2CA*, *IKBKB*, *DUSP3*, *MAP3K1*, *PPP2R1A*, *S100A12*, *UBE2V1*, *ALPK1*
TLR5 cascade	0.009472	*DUSP4*, *NFKBIA*, *PPP2CA*, *IKBKB*, *DUSP3*, *MAP3K1*, *PPP2R1A*, *S100A12*, *UBE2V1*, *ALPK1*
TLR10 cascade	0.009472	*DUSP4*, *NFKBIA*, *PPP2CA*, *IKBKB*, *DUSP3*, *MAP3K1*, *PPP2R1A*, *S100A12*, *UBE2V1*, *ALPK1*

TLR, toll-like receptor; MAPK, mitogen-activated protein kinase.
