# Supplementary material for: Novel potential drugs for the treatment of primary open-angle glaucoma using protein-protein interaction network analysis
Source: Genomics Inform. 2023 Mar 31;21(1):e6. doi: 10.5808/gi.22070 (PMC10085733; doi:10.5808/gi.22070)
Supplement: Supplementary Table 7. — Downregulated genes based on molecular function enrichment analysis [file gi-22070-Supplementary-Table-7.pdf]

Supplementary Table 7. Downregulated genes based on molecular function enrichment analysis

Molecular function	p-value	Genes
Protein binding	2.23E-16	*ZNF296, SMC5, RBPJ, ABCA12, CLDN1, ALKBH6, FAM110A, PPP4R1, PPP4R2, CAPNS2, SOX15, MYB, NAMPT, CKMT1B, CYP1B1, PRSS8, VPS36, TYW3, PIH1D1, MED4, SULT2B1, ACE2, SCO1, DAAM1, ETHE1, DDIT4, TMEM79, CLDN7, PSME4, PSME1, RNF122, ABCB7, SHMT2, MAPKAP1, FPR1, LYPD2, IL20RB, GATA3, IQGAP1, MRPL12, HIF1A, C3, S100A12, SFN, S100A14, S100A11, PROM2, GPIHBP1, ABCA1, BRAP, PHC2, JAG1, MRPL28, CGN, GPN2, FOSL2, TBL3, GJB2, GIPC1, GJB5, CEP57, SERPINA3, BTG1, PHF23, TCF20, MED16, AREG, IKBKB, CSRP2, MECOM, IMPA2, RBPMS2, PLEKHN1, TPSAB1, TEAD3, EDARADD, DUSP4, DUSP5, FGFBP1, SERPINB1, DUSP3, FGFBP2, DUSP1, SPHK1, MAMDC2, RIPK4, PAX6, TMC6, F3, SERPINB5, BACE2, SLC7A6, MED25, PIGA, RARA, PICK1, PLIN2, CCL13, CTDP1, PSMB3, MFN2, DMD, FLNC, LRRC26, CDKN2B, ZNF581, BIK, SMAGP, NDE1, MOCOS, BBOX1, FMO1, WWP1, MKLN1, XAB2, NUDT21, LRG1, FABP5, C6ORF47, PSMC1, ZYX, LGR4, ERO1A, UBE2D3, HP, CXCL17, DEFB1, RORA, BZW2, CDC14B, ZFP36, SLN, KPNA6, PIM1, CHP2, ZNF207, PAPOLA, TOM1, MTPAP, RAB2A, RNF43, USP7, S100A2, RPS5, FBXO11, SDCBP2, TUBA4A, MTNR1A, OBSCN, ZNF438, RAB38, PKP3, S100A9, SQSTM1, S100A8, COL17A1, NOTCH1, STK39, DPT, GTF2E2, KLC3, STK38, EIF4EBP1, AP1M2, EIF2B4, MYPOP, CCDC12, RBPMS, ZG16B, ZBTB16, FBXO34, FOXN3, CYP4F12, HOPX, DRAP1, STK24, BCL6, TMEM11, BCL3, LCN2, RNF181, ZNF777, EIF4G3, RSL24D1, ALK, PPP1R13B, SH2D4A, CDCA7L, FLT4, MSLN, ADAMTS4, PPP6C, TRIM63, VWF, DST, GPX3, VPS37C, RAD23A, VPS37B, RABGGTA, TNFRSF1A, PTP4A1, ARL4D, ARL4A, PTP4A3, DNAJC7, SCGB2A1, SLC25A10, TNFRSF21, BIRC3, ZNF750, PPP1R15A, CEBPD, GTF3C5, MTFP1, TYRP1, ATP10B, AMBRA1, HDAC8, PPM1G, CDC42, SH3BP1, RAB25, TP53INP1, UBQLN1, TNNI2, ZNF622, POLR2J, KCNJ2, MYO10, FOXJ2, KLF4, ASS1, SYT17, CLEC2B, RAB18, PDCD4, EIF3A, IL1RN, HDAC11, MT1M, ZDHHC5, MT1X, CALML3, AQP5, AQP3, NUBP2, MT2A, TRIM29, PPP2R1A, RASSF6, ARL6IP4, KAT8, TBC1D22A, EIF2A, KRT4, IL1R1, ACTN1, TALDO1, CSNK1E, KRT5, RUNX3, THAP4, INPP4B, CARHSP1, AZGP1, MT1A, SLPI, RRAGD, MALL, TRIB3, TRIM16, EZR, ALPK1, TRIB1, PLPP2, PTGES, LLGL2, CDS1, SDC4, MAOA, CSTF2, TWIST2, PPL, UGCG, DHX37, TSPAN1, VASP, NTNG1, ZRANB1, GADD45B, DTNB, XRCC5, RASSF7, MYO5A, CNFN, PUM3, DERA, EXT1, PM20D2, CC2D1A, MON1B, CSRNP1, GFM1, CCDC120, HILPDA, KRT24, ADIRF, PCSK5, RAC1, HRAS, CTSD, RBBP8NL, VAV3, CAMLG, AKR1A1, RGMA, GAK, CNKSR1, CEACAM1, CDCP1, CEACAM6, CEACAM5, WAC, RHOU, TFF1, VGLL1, VGLL4, CD44, AVPI1, TNXB, ADH1A, AKAP8, GNAI3, TYMP, PPP2CA, PGRMC2, PERP, CD55, MPZL2, MAP3K1, GDF15, KRT13, DOHH, TMUB2, SH3RF2, KRT19, KRT18, KRT17, DLC1, KRT15, KRT14, SAA2, FCN3, PLVAP, MIDN, ZFAND5, UBE3A, CMIP, TGM1, CHCHD6, KHSRP, FAM3B, CAPN1, ARID2, CAST, OSR2, RALBP1, KDM2A, SLX1B, LMO4, SSU72, WBP11, SCNN1A, UBE2V1, MASP1, ABCG1, UBIAD1, HTRA3, PXN, DTX2, SLC1A5, RPF2, FBLN2, NDRG1, STK3, FAM83H, FAM83F, RNASEH1, STAP2, MYH11, OTX1, NDUFV2, MORC2, MARK2, FAM83A, POU2F1, SMAD3, AUTS2, CBX3, TBCD, BMP7, ANKHD1, SELP, NFKBIA, EFNA1, BMP1, HOOK1, MKRN1, BHLHE40, JMY, RSPH14, KDM5B, TNFAIP6, USP32, SLC2A3, CXCL1, YBX3, RND3, UPK1B, NAPRT, FLOT1, FLOT2, IFT57, MMP3, ARHGEF16, TMPRSS11D, TAPBP, ARHGAP10, DCT, ELF3, IRF1, OTUB1, ATP6V1B1, RBM47, ACVR1B, TOB1, ERICH5, PDLIM1, MUC1, RASD1, MAT2A, MLANA, PRRG2, FAM120B, MXI1, MARVELD3, EPS8L2, SASH1, HES4, TMEM30B, LAMB3, EYA2, LIMK2, MAPK13, SH3YL1, MEIS1, SPRY2, S100P, RAN, BNIPL*

Cadherin binding	2.69E-05	*IQGAP1, BZW2, EVPL, NDRG1, PPL, COBLL1, STK38, EFHD2, EPS8L2, SFN, MARK2, EIF2A, SPTBN2, VASP, CAST, ARHGEF16, CGN, STK24, GIPC1, S100P, PKP3, EZR, RAN, CC2D1A, CDH19*
Calcium-dependent protein binding	8.70E-05	*SELP, S100A2, TNNT3, VPS37C, S100A12, S100P, MASP1, VPS37B, S100A14, S100A9, S100A8, S100A11*
Identical protein binding	1.78E-04	*CEP57, ALK, PLVAP, DEFB1, AQP5, AQP3, CLDN1, TGM1, IKBKB, TRIM29, NAMPT, RBPMS2, ARL6IP4, TPSAB1, MTPAP, S100A2, VWF, GPX3, SORD, SDCBP2, THAP4, ACE2, CEACAM1, DAAM1, CEACAM6, CEACAM5, ETHE1, TMEM79, CLDN7, VWA1, PICK1, EZR, SQSTM1, NOTCH1, CEBPD, SDC4, SHMT2, HTRA3, UAP1, GATA3, STK3, CDC42, MAT2A, UBQLN1, S100A12, SFN, FLNC, MORC2, KCNJ2, FAM83A, BRAP, PHC2, MYPOP, POU2F1, SMAD3, CBX3, ZBTB16, NDE1, FOXJ2, TYSND1, BBOX1, ASS1, DLK2, NFKBIA, DRAP1, NUDT21, MKLN1, PM20D2, GJB2, BMP1, CLEC2B, BCL6, FABP5, HOOK1, GIPC1, LCN2, MUC20, BNIPL*
Myosin phosphatase activity	4.06E-04	*DUSP4, PPP2CA, PPP6C, DUSP5, DUSP3, CTDP1, DUSP1, SSU72, PPM1G, CDC14B*
Structural molecule activity	5.05E-04	*JAG1, LAMB3, DST, KRT13, KRT24, EVPL, CLDN1, PPL, KRT19, UPK1B, KRT18, KRT17, KRT15, KRT14, CLDN7, EIF3A*
Cadherin binding involved in cell-cell adhesion	0.001684	*PDLIM1, KRT18, TRIM29, PKP3, S100A11*
Serine-type endopeptidase activity	0.001912	*CFD, MMP1, HTRA3, MMP3, HP, TYSND1, PCSK5, F3, KLK11, KLK12, TMPRSS11D, BMP1, MASP1, PRSS8, TPSAB1*
Transcription coactivator binding	0.002538	*MED25, SMAD3, RARA, RORA, GATA3, HIF1A, VGLL4*
Actin binding	0.002901	*VASP, MYO10, DST, ACTN1, MYO5A, CGN, PDLIM1, DIAPH2, CEACAM1, DAAM1, HOOK1, GIPC1, TNNT3, JMY, TNNI2, MYH14, PICK1, EPS8L2, DMD, EZR, SPTBN2*
Peptidase activity	0.003318	*ADAMTS4, BMP1, MMP1, MMP3, MASP1, CAPN1, PCSK5, CTSD, KLK12, TMPRSS11D*
RAGE receptor binding	0.003588	*FPR1, S100A12, S100A9, S100A8*
Zinc ion binding	0.004515	*KDM5B, SHMT2, ADH1B, ADH1A, MT1M, ZFAND5, AKAP8, RORA, MT1X, DTX2, GATA3, MT2A, TRIM29, ZSWIM8, S100A12, DMD, ZNF622, MORC2, ADAMTS9, BRAP, PHC2, TRIM63, KDM2A, MAP3K1, SMAD3, DTNB, MMP1, MMP3, SORD, BBOX1, KLF4, FBXO11, RABGGTA, ACE2, MT1A, BMP1, RARA, TRIM16, S100A9, SQSTM1, S100A8*
Protein tyrosine/serine/threonine phosphatase activity	0.005652	*PTP4A1, DUSP5, PTP4A3, DUSP3, DUSP1, CDC14B*
mRNA binding	0.006641	*RBM47, CLUH, RBPMS, RPS5, CSTF2, MRPL12, PUM3, NUDT21, ZFP36, SLPI, ESRP2, KHSRP, RBPMS2, EIF4G3, EIF3A, EIF2A*
Serine-type peptidase activity	0.007907	*CFD, RELN, HTRA3, PRSS8, TPSAB1, KLK11, KLK12*
Transcription factor activity, sequence-specific DNA binding	0.008361	*CSRNP1, ZNF395, ZNF296, RORA, GATA3, RBPJ, HIF1A, MECOM, SOX15, OTX1, TEAD3, ZNF581, SMAD3, BCL11A, ZBTB16, PRRX2, PAX6, FOXN3, KLF4, RUNX3, FOSL2, ELF3, BCL6, IRF1, BHLHE40, KLF9, ZNF438, RARA*
Protein serine/threonine phosphatase activity	0.008571	*PPP2CA, PPP6C, PPA2, DUSP1, PPP2R1A, PPM1G, CDC14B*
