# Supplementary material for: Novel potential drugs for the treatment of primary open-angle glaucoma using protein-protein interaction network analysis
Source: Genomics Inform. 2023 Mar 31;21(1):e6. doi: 10.5808/gi.22070 (PMC10085733; doi:10.5808/gi.22070)
Supplement: Supplementary Table 8. — Downregulated genes based on cell component enrichment analysis [file gi-22070-Supplementary-Table-8.pdf]

Supplementary Table 8. Downregulated genes based on cell component enrichment analysis

Cell component	p-value	Genes
Extracellular exosome	1.70E-13	*PLVAP, IL1RN, UBE2D3, HP, CALML3, DEFB1, AQP5, TGM1, PPP2R1A, NAMPT, TOM1, FAM3B, PRSS8, CAPN1, VPS36, RAB2A, RPS5, ACTN1, SLC6A14, TALDO1, KRT5, SDCBP2, TUBA4A, SULT2B1, ACE2, AZGP1, SPINT1, SLPI, SCNN1A, PSME1, UBE2V1, EZR, S100A9, SQSTM1, S100A8, CFD, SDC4, SHMT2, SLC1A5, IQGAP1, EVPL, PPL, NDRG1, C3, COBLL1, MYH14, MYH11, SFN, S100A14, TSPAN1, S100A11, PROM2, VASP, ZG16B, MYO5A, STK24, GIPC1, LCN2, CC2D1A, CLIC6, ALK, SERPINA3, PIGR, KRT24, ADIRF, SLC2A3, UPK1B, NAPRT, FLOT1, FLOT2, RAC1, CTSD, BCAS1, SERPINB1, VWF, GPX3, VPS37C, AKR1A1, SORD, LSR, TMC6, VPS37B, TMC5, TMC4, TMPRSS11D, CEACAM1, DNAJC7, CEACAM5, VWA1, CD44, ATP6V1B1, OTUB1, LAMA5, TNXB, GNAI3, PPP2CA, CDC42, MUC1, RAB25, PSMB3, GCNT3, EPS8L2, CD55, LRRC26, GDF15, GSR, KRT13, WWP1, BBOX1, KLK11, ASS1, KRT19, KRT18, LRG1, FABP4, FABP5, KRT15, KRT14, SAA2, S100P, RAN*
Cytosol	5.53E-13	*RPL32, ABCA12, NUBP2, FAM110A, ALKBH5, MT2A, CAPNS2, PPP2R1A, NAMPT, MYB, VPS36, KRT4, ACTN1, TALDO1, KRT5, CSNK1E, RUNX3, SULT2B1, INPP4B, CARHSP1, MT1A, PPA2, DAAM1, PSME4, RRAGD, DDIT4, PSME1, TRIB3, TRIM16, EZR, ALPK1, TRIB1, LLGL2, ZNF395, CASZ1, MAOA, SHMT2, MAPKAP1, IQGAP1, HIF1A, EVPL, PPL, S100A12, SFN, STXBP5, C19ORF33, SPTBN2, BRAP, VASP, ZRANB1, BCL11A, XRCC5, MYO5A, TYSND1, MRPL28, DERA, FERMT1, GJB2, GIPC1, CC2D1A, CEP57, HILPDA, ADK, KRT24, ADIRF, CROT, IKBKB, MECOM, IMPA2, RAC1, HRAS, EDARADD, VAV3, DUSP3, SPHK1, AKR1A1, PAX6, GAK, CEACAM7, TNNT3, RARA, PICK1, RHOU, PLIN2, CD44, ADH1B, ADH1A, TYMP, PPP2CA, PSMB3, ZSWIM8, MFN2, DMD, FLNC, CDKN2B, MAP3K1, NDE1, MOCOS, GSR, KRT13, BBOX1, WWP1, DOHH, MKLN1, KRT19, FABP4, KRT18, FABP5, KRT17, FABP6, PSMC1, DLC1, KRT15, KRT14, ZYX, TACC2, MIDN, UBE2D3, UBE3A, CMIP, TGM1, ZFP36, CHCHD6, KHSRP, FPGS, KPNA6, PIM1, TOM1, CHP2, CAPN1, RAB2A, CAST, RALBP1, USP7, DIMT1, RPS5, SSU72, WBP11, FBXO11, SDCBP2, TUBA4A, OBSCN, SHPK, ZNF438, RAB38, MASP1, UBE2V1, S100A9, SQSTM1, S100A8, NOTCH1, PXN, STK39, UAP1, RASAL1, NDRG1, GTF2E2, STK3, STK38, EIF4EBP1, MYH14, MYH11, STAP2, RHPN2, NDUFV2, MORC2, AP1M2, EIF2B4, SMAD3, RBPMS, KLHL25, ZBTB16, NFKBIA, DIAPH2, STK24, HOOK1, MKRN1, BCL3, LCN2, EIF4G3, KDM5B, PPP1R13B, SH2D4A, USP32, CDCA7L, FLT4, YBX3, PPP6C, NAPRT, DBT, GPX2, DST, ARHGEF16, SORD, RAD23A, RABGGTA, ARHGAP10, ARL4A, DNAJC7, DCT, ELF3, IRF1, ATP6V1B1, OTUB1, BIRC3, PPP1R15A, AMBRA1, ACVR1B, CDC42, MAT2A, SH3BP1, TP53INP1, MXI1, UBQLN1, TNNI2, EPS8L2, ZNF622, BCO1, MYO10, EYA2, KLF4, ASS1, MAPK13, KLF9, PDCD4, RAB18, SPRY2, RAN, EIF3A, BNIPL*
Cytoplasm	1.47E-11	*IL1RN, RPL32, MT1M, SMC5, MT1X, ABCA12, RBPJ, AQP3, CLDN1, ALKBH6, NUBP2, FAM110A, MT2A, PPP4R2, TRIM29, PPP2R1A, SOX15, NAMPT, EIF2A, ACTN1, PIH1D1, TYW3, TALDO1, KRT5, CSNK1E, RUNX3, SULT2B1, THAP4, INPP4B, ACE2, CARHSP1, MT1A, PPA2, ETHE1, RRAGD, DDIT4, PSME1, HECA, TRIM16, EZR, TRIB1, RNF122, LLGL2, ZNF395, SHMT2, MAPKAP1, FPR1, TWIST2, IQGAP1, HIF1A, EVPL, PPL, DHX37, S100A12, SFN, STXBP5, TSPAN1, S100A11, BRAP, VASP, ZRANB1, GADD45B, DTNB, BCL11A, RASSF7, MYO5A, CNFN, DERA, FERMT1, GIPC1, PLCH2, MON1B, CLIC6, CEP57, BTG1, PHF23, CITED4, ADM, IKBKB, CSRP2, IMPA2, RBPMS2, RAC1, HRAS, VAV3, DUSP4, DUSP5, CLUH, DUSP3, CAMLG, DUSP1, SPHK1, RIPK4, PAX6, TMC6, SERPINB5, GAK, RARA, PICK1, CTDP1, GNAI3, RELN, PSMB3, DMD, FLNC, LRRC26, CDKN2B, MAP3K1, GDF15, NDE1, WWP1, PODN, MKLN1, NUDT21, FABP4, KRT18, FABP5, FABP6, RNF149, PSMC1, DLC1, KRT14, ZYX, TACC2, MIDN, ZFAND5, BZW2, CDC14B, ZFP36, KHSRP, FPGS, ZNF207, KPNA6, PAPOLA, PIM1, TOM1, CHP2, CAPN1, CAST, RPS5, FBXO11, SDCBP2, TUBA4A, SHPK, SCNN1A, UBE2V1, PKP3, S100A9, SQSTM1, UBIAD1, PXN, STK39, DTX2, NDRG1, STK3, FAM83H, ALDH3B2, KLC3, RNASEH1, STK38, EIF4EBP1, MYH14, MYH11, MORC2, MARK2, FAM83A, EIF2B4,*

		SMAD3, KLHL25, AUTS2, TBCD, CYP4F12, HOPX, ANKHD1, BSPRY, NFKBIA, STK24, HOOK1, BCL3, JMY, BHLHE40, RNF181, PPP1R13B, SH2D4A, CDCA7L, YBX3, PPP6C, DBT, BCAS1, GPX2, TRIM63, DST, RAD23A, VPS37B, RABGGTA, PTP4A1, ARL4D, ARL4A, PTP4A3, DNAJC7, IRF1, TNFRSF21, ATP6V1B1, BIRC3, PPP1R15A, TYRP1, AMBRA1, HDAC8, TOB1, PPM1G, PDLIM1, CDC42, RASD1, SH3BP1, MARVELD3, UBQLN1, EPS8L2, SASH1, LIMK2, KLF4, ASS1, MAPK13, PDCD4, S100P, RAN, EIF3A, BNIPL
Nucleoplasm	2.67E-06	*UBE2D3, SMC5, RORA, RBPJ, AQP3, ALKBH6, CMIP, CDC14B, ALKBH5, PPP4R2, KHSRP, MYB, ZNF207, KPNA6, PAPOLA, PIM1, CHP2, KAT8, MTPAP, ARID2, USP7, KDM2A, DIMT1, SLX1B, RPS5, SSU72, THOC3, CSNK1E, WBP11, MED4, FBXO11, RUNX3, SDCBP2, ETHE1, PSME4, RRAGD, ZNF438, PSME1, TRIB3, MASP1, UBE2V1, PKP3, BCORL1, S100A9, SQSTM1, ZNF395, CASZ1, NOTCH1, MAPKAP1, CSTF2, TWIST2, STK39, UAP1, DTX2, GATA3, RPF2, HIF1A, GTF2E2, DHX37, C19ORF33, NDUFV2, MORC2, TSPAN1, MARK2, BRAP, PHC2, ZRANB1, POU2F1, SMAD3, RBPMS, NOP16, CBX3, BCL11A, XRCC5, PUM3, FOSL2, DERA, NFKBIA, PM20D2, TBL3, BCL6, STK24, BCL3, JMY, RSL24D1, KDM5B, PPP1R13B, BTG1, FLT4, PHF23, CDCA7L, CITED4, HILPDA, ADK, ADIRF, TCF20, MED16, PPP6C, MECOM, SH3BGRL2, HRAS, TEAD3, DUSP4, DUSP5, DUSP3, DST, PAX6, RAD23A, RABGGTA, ARL4A, MED25, TOX2, DNAJC7, ELF3, ESRP2, IRF1, RARA, WAC, SLC25A10, VGLL1, OTUB1, BIRC3, CTDP1, CEBPD, GTF3C5, ADH1B, ADH1A, AKAP8, HDAC8, PPM1G, PSMB3, TP53INP1, MXI1, UBQLN1, ZNF622, PCDH1, POLR2J, EYA2, SMAGP, FOXJ2, KLF4, ASS1, NUDT21, XAB2, SH3RF2, MKLN1, FABP5, PSMC1, KLF9, KLHL8, S100P, TACC2, RAN, EIF3A*
Adherens junction	7.51E-06	*NOTCH1, JAG1, TBCD, NDRG1, PDLIM1, CEACAM1, KRT18, SH3BP1, POF1B, TRIM29, FLOT1, ZYX, FLOT2, PKP3, EZR, S100A11, CDH19, LLGL2*
Extracellular space	1.73E-05	*ERO1A, SERPINA3, PIGR, CSF3, FCN3, IL1RN, TNFAIP6, HILPDA, HP, CXCL17, ADM, DEFB1, LAMC2, CXCL1, MSLN, PCSK5, AREG, GLDN, ADAMTS4, NAMPT, FAM3B, FAM3D, PRSS8, TPSAB1, ADAMTS9, CTSD, RBBP8NL, EIF2A, SERPINB1, FGFBP1, FGFBP2, EDN2, VWF, GPX3, ACTN1, MMP3, AKR1A1, SORD, F3, SERPINB5, TNFRSF1A, ACE2, AZGP1, SPINT1, SLPI, CEACAM6, SCGB2A1, CHI3L2, VWA1, TFF1, MASP1, EZR, S100A9, S100A8, CFD, COL17A1, LAMA5, CCL13, TNXB, COL11A1, DPT, TFPI, LILRA5, C3, MUC1, RELN, PRRG2, S100A12, SFN, S100A14, S100A11, SPTBN2, GDF15, ZG16B, LGI4, PODN, BMP7, KLK11, KLK12, SELP, BMP1, KRT18, LRG1, FABP5, LCN2*
Cytoskeleton	1.93E-05	*PXN, STK39, KRT24, AMBRA1, RND3, EVPL, PPL, PDLIM1, FAM83H, S100A12, DMD, FLNC, PLEKHN1, RAC1, MPZL2, VASP, LRRC26, AUTS2, DST, KRT13, TUBA4A, FERMT1, KRT19, KRT18, KRT17, DNAJC7, KRT15, KRT14, JMY, SPRY2, PICK1, EZR, S100A9, S100A8*
Plasma membrane	1.11E-04	*IL1RN, HDAC11, UBE2D3, ZDHHC5, AQP5, ABCA12, AQP3, CLDN1, GLDN, RAPGEFL1, TGM1, CAPNS2, DUOXA1, NAMPT, TOM1, PIM1, CHP2, PRSS8, CAPN1, ARID2, RNF43, RALBP1, IL1R1, ACTN1, SLC6A14, UNC93A, SDCBP2, ACE2, MTNR1A, OBSCN, ADCY9, SPINT1, DAAM1, CLDN7, MALL, SLCO2A1, SCNN1A, RAB38, TRIB3, TRIM16, PKP3, EZR, BCORL1, S100A9, PLPP2, S100A8, ABCG1, LLGL2, COL17A1, NOTCH1, SDC4, MAPKAP1, PXN, FPR1, LYPD2, IL20RB, UAP1, RASAL1, SLC1A5, IQGAP1, PPL, NDRG1, IGSF9, C3, HCAR3, TSPAN9, S100A12, STXBP5, STAP2, C19ORF33, RHPN2, TSPAN1, MARK2, SPTBN2, GPIHBP1, PROM2, ABCA1, NTNG1, VASP, JAG1, SMAD3, DTNB, XRCC5, ZBTB16, CGN, SELP, EFNA1, NFKBIA, GJB2, TMEM11, BCL3, PLCH2, MUC20, MUC21, CC2D1A, CLIC6, ALK, PIGR, PPP1R13B, FLT4, ADK, SLC2A3, MSLN, RND3, UPK1B, EFHD2, FLOT1, FLOT2, PLEKHN1, RAC1, HRAS, VAV3, FGFBP1, SPHK1, LSR, TMC6, VPS37B, F3, RABGGTA, RGMA, TMPRSS11D, TNFRSF1A, CNKSR1, ARHGAP10, ARL4D, BACE2, SLC7A6, CEACAM1, ARL4A, PTP4A3, CDCP1, CEACAM7, DCT, CEACAM6, CEACAM5, RARA, PICK1, RHOU, PLIN2, TNFRSF21, CD44, CDH19, ADH1B, ADH1A, GNAI3, ATP10B, HDAC8, ACVR1B, TFPI, AGPAT2, LILRA5, PPP2CA, CDC42, GNA15, MUC1, RELN, RASD1, MLANA, PRRG2, PERP, UBQLN1, SLITRK6, EPS8L2, DMD, FLNC, PCDH1, CD55, KCNJ2, MPZL2, LRRC26, TMEM30B, MYO10, SMAGP, KCNJ13, WWP1, SYT17, KRT19, CLEC2B, FABP5, KLF9, ZYX, RAB18, SPRY2, TACC2, ACKR1, LGR4*

Focal adhesion	1.32E-04	*SDC4, PXN, AMBRA1, IQGAP1, RND3, ALKBH6, PDLIM1, CDC42, CSRP2, TSPAN9, FLOT1, FLOT2, FLNC, RAC1, CAPN1, VASP, DST, ACTN1, STARD8, RPS5, GAK, FERMT1, DLC1, ZYX, RHOU, EZR, CD44*
Melanosome	1.35E-04	*RAB2A, DCT, MLANA, TYRP1, FLOT1, MYO5A, RAB38, MYH11, RAC1, SLC1A5, CTSD, RAN*
Lateral plasma membrane	1.69E-04	*GJB2, CEACAM1, PPP2R1A, CLDN7, TBCD, DMD, IQGAP1, CLDN1, MARK2, ATP6V1B1*
Nucleus	1.89E-04	*ZNF296, HDAC11, MT1M, SMC5, MT1X, RBPJ, ALKBH6, NUBP2, FAM110A, ALKBH5, MT2A, PPP4R2, PPP2R1A, SOX15, MYB, CYP1B1, ARL6IP4, KAT8, VPS36, KRT4, ACTN1, PIH1D1, TALDO1, KRT5, CSNK1E, MED4, RUNX3, SULT2B1, THAP4, AZGP1, MT1A, PSME4, RRAGD, TRIB3, HECA, BCORL1, TRIB1, ZNF395, CASZ1, SHMT2, TWIST2, GATA3, IQGAP1, HIF1A, WDR70, S100A12, SFN, C19ORF33, S100A11, PHC2, ZRANB1, GADD45B, DTNB, BCL11A, XRCC5, FOSL2, CC2D1A, CEP57, SERPINA3, CSRNP1, BTG1, PHF23, CITED4, ADK, ADIRF, TCF20, MED16, AREG, IKBKB, CSRP2, MECOM, RAC1, TEAD3, DUSP4, DUSP5, DUSP3, DUSP1, SPHK1, PAX6, MED25, TOX2, WAC, RARA, PLIN2, VGLL1, VGLL4, CTDP1, AKAP8, PPP2CA, PSMB3, DMD, CDKN2B, ZNF581, GDF15, KRT13, WWP1, XAB2, SH3RF2, NUDT21, FABP4, FABP5, FABP6, PSMC1, DLC1, KRT15, KRT14, ZYX, MIDN, UBE2D3, UBE3A, RORA, CDC14B, FOXQ1, ZFP36, KHSRP, ZNF207, KPNA6, PAPOLA, PIM1, CHP2, RAB2A, OSR2, RALBP1, USP7, LMO4, WBP11, FBXO11, ZNF438, UBE2V1, PKP3, S100A9, S100A8, UBIAD1, NOTCH1, NDRG1, STK3, STK38, EIF4EBP1, OTX1, MORC2, POU2F1, MYPOP, SMAD3, AUTS2, CBX3, ZBTB16, FOXN3, HOPX, NFKBIA, DRAP1, BCL6, STK24, BCL3, JMY, BHLHE40, ZNF777, BRWD3, KDM5B, PPP1R13B, USP32, CDCA7L, YBX3, TRIM63, DST, RAD23A, PTP4A1, ARL4A, PTP4A3, ELF3, ESRP2, IRF1, SLC25A10, OTUB1, BIRC3, ZNF750, LAMA5, RBM47, CEBPD, AMBRA1, HDAC8, TOB1, PPM1G, MUC1, RASD1, SH3BP1, FAM120B, TP53INP1, MXI1, UBQLN1, TNNI2, POLR2J, HES4, EYA2, LIMK2, PRRX2, FOXJ2, ASS1, MAPK13, MEIS1, KLF9, PDCD4, SPRY2, S100P, RAN, BNIPL*
Cornified envelope	2.24E-04	*TGM1, KRT17, KRT14, PKP3, CAPN1, CNFN, PPL, EVPL, SERPINB5*
Extracellular region	6.72E-04	*SERPINA3, CSF3, FCN3, TNFAIP6, FLT4, HP, ADM, DEFB1, LAMC2, CXCL1, MSLN, PCSK5, AREG, ADAMTS4, NAPRT, FAM3B, FAM3D, PRSS8, CAPN1, TPSAB1, ADAMTS9, CTSD, SERPINB1, FGFBP1, EDN2, VWF, GPX3, MMP1, IL1R1, ACTN1, MAMDC2, MMP3, TUBA4A, TMPRSS11D, TNFRSF1A, ACE2, AZGP1, CDCP1, SPINT1, SLPI, CEACAM7, CEACAM5, CHI3L2, TFF1, PLIN2, MASP1, S100A9, S100A8, CFD, COL17A1, LAMA5, CCL13, TNXB, NOTCH1, COL11A1, HTRA3, LYPD2, TFPI, FBLN2, LILRA5, C3, RELN, CHSY1, PRRG2, S100A12, S100A11, CD55, GPIHBP1, NTNG1, JAG1, LAMB3, GDF15, XRCC5, LGI4, BMP7, KLK12, DERA, EFNA1, BMP1, LRG1, FABP5, LCN2, S100P, MUC20*
Apical plasma membrane	8.81E-04	*NOTCH1, JAG1, SLC6A14, STK39, AKR1A1, AQP5, CYP4F12, CLDN1, ACE2, MUC1, CEACAM1, CEACAM7, CEACAM6, CEACAM5, SCNN1A, RAB18, EZR, MUC20, CD44, PROM2, ATP6V1B1, SPTBN2, GPIHBP1*
Intermediate filament	0.001118	*KRT19, KRT18, KRT17, DST, KRT4, KRT15, KRT14, KRT13, KRT24, KRT5, PPL, EVPL*
Cortical actin cytoskeleton	0.001241	*ACTN1, DLC1, FLOT1, FLOT2, DMD, IQGAP1, SPTBN2, LLGL2*
Secretory granule lumen	0.00127	*CFD, C3, SERPINA3, SERPINB1, XRCC5, S100A12, S100P, S100A9, S100A8, S100A11, DERA*
Membrane raft	0.001769	*ABCA1, SDC4, GNAI3, IQGAP1, TNFRSF1A, PPP2CA, IKBKB, ACE2, DLC1, MALL, FLOT1, EFHD2, DMD, CTSD, CD55, PROM2, BIRC3*
Anchored component of membrane	0.003521	*EFNA1, CEACAM7, CEACAM6, CEACAM5, LYPD2, MSLN, TFPI, CD55, RGMA, GPIHBP1*
Proteasome complex	0.004621	*PSMB3, PSMC1, PSME4, PSME1, UBQLN1, UBE3A, RAD23A*
Desmosome	0.005199	*POF1B, PERP, PKP3, PPL, EVPL*

Cell projection	0.0052	*VASP, DST, ACTN1, SLC2A3, RND3, CDC42, RHOU, DMD, RAC1, EZR, CD44, PROM2, SPTBN2*
RNA polymerase II transcription factor complex	0.006533	*DRAP1, POU2F1, CEBPD, CBX3, LMO4, MXI1, MYB, RARA, HIF1A, FOSL2*
Basolateral plasma membrane	0.007772	*ABCA1, STK39, AQP3, CLDN1, SLC7A6, CEACAM5, CLDN7, FLOT1, FLOT2, EZR, MUC20, CD44, PROM2, ATP6V1B1, GPIHBP1*
Intracellular membrane-bounded organelle	0.008392	*ERO1A, CASZ1, CTDP1, STK39, AMBRA1, CROT, ABCA12, RND3, CYP1B1, FLOT2, RAC1, PCDH1, MTPAP, ADAMTS9, TSPAN1, AP1M2, SPTBN2, ABCA1, ZRANB1, POU2F1, NOP16, SPHK1, VPS37C, FMO1, TALDO1, RAD23A, VPS37B, WBP11, CYP4F12, RUNX3, GAK, SULT2B1, XAB2, DIAPH2, SLC7A6, LRG1, DCT, BCL3, RRAGD, SQSTM1, LLGL2*
Bicellular tight junction	0.009747	*VASP, SH3BP1, POF1B, CLDN7, MARVELD3, TBCD, LSR, CGN, YBX3, CLDN1*
Microvillus membrane	0.009824	*CEACAM1, S100P, EZR, MUC20, PROM2*
