# Supplementary material for: Novel potential drugs for the treatment of primary open-angle glaucoma using protein-protein interaction network analysis
Source: Genomics Inform. 2023 Mar 31;21(1):e6. doi: 10.5808/gi.22070 (PMC10085733; doi:10.5808/gi.22070)
Supplement: Supplementary Table 9. — Downregulated genes based on biological process enrichment analysis [file gi-22070-Supplementary-Table-9.pdf]

Supplementary Table 9. Downregulated genes based on biological process enrichment analysis

Biological process	p-value	Genes
Epidermis development	1.23E-07	*COL17A1, LAMB3, LAMC2, KRT5, EVPL, AGPAT2, UGCG, SPINT1, DCT, FABP5, KRT15, GJB5, KRT14, FLOT2, ZNF750*
Negative regulation of cell proliferation	2.01E-07	*NOTCH1, BTG1, DPT, ADM, CXCL1, GATA3, AMBRA1, RBPJ, TOB1, NDRG1, STK3, TP53INP1, CYP1B1, ARID2, HRAS, S100A11, CDKN2B, FGFBP1, SMAD3, DUSP1, ZBTB16, PODN, KLF4, BMP7, SULT2B1, AZGP1, BCL6, FABP6, IRF1, DLC1, RARA, SPRY2, ZNF777, TFF1, PTGES, BNIPL*
Actin cytoskeleton organization	4.71E-06	*VASP, TNXB, LIMK2, ACTN1, STARD8, CXCL1, RND3, PDLIM1, CDC42, CSRP2, POF1B, DAAM1, BCL6, DLC1, RHOU, DMD, FLNC, RAC1, SPTBN2*
Signal transduction	6.69E-06	*ALK, IL1RN, TNFAIP6, ADM, CXCL1, GLDN, PPP4R1, IMPA2, NAMPT, RASSF6, TOM1, FAM3B, FAM3D, MAP3K6, HRAS, EDARADD, FGFBP1, TRIM63, CAMLG, STARD8, CSNK1E, TNFRSF1A, ARHGAP10, INPP4B, CEACAM1, ADCY9, CEACAM6, RARA, TFF1, PLPP2, TNFRSF21, PTGES, CDS1, CCL13, PXN, AKAP8, FPR1, STK39, RASAL1, GATA3, IQGAP1, ACVR1B, HIF1A, NDRG1, STK3, TYMP, C3, FAM83H, FAM83F, RASD1, SH3BP1, SFN, RHPN2, S100A11, FAM83A, MYO10, GDF15, RASSF7, WWP1, EXT1, MKLN1, BMP1, STK24, DLC1, ZYX*
Apoptotic process	7.33E-05	*PPP1R15A, CSRNP1, PPP1R13B, MTFP1, UBE2D3, YBX3, STK3, PPP2CA, MECOM, PPP2R1A, TP53INP1, RASSF6, FAM3B, PIM1, MFN2, S100A14, IFT57, GADD45B, BIK, ZBTB16, RASSF7, TNFRSF1A, NFKBIA, CEACAM6, IRF1, CEACAM5, DLC1, DDIT4, LCN2, PDCD4, S100A9, SQSTM1, S100A8, TNFRSF21, BIRC3, BNIPL*
Establishment of skin barrier	1.44E-04	*UGCG, TMEM79, SFN, LSR, KLF4, ABCA12, CLDN1*
Inactivation of MAPK activity	2.04E-04	*DUSP4, PPP2CA, DUSP5, DUSP3, DUSP1, PPP2R1A*
Endothelial cell migration	3.62E-04	*PTP4A3, EDN2, S100A2, GIPC1, PXN, CYP1B1, S100P*
Negative regulation of transcription, DNA-templated	4.41E-04	*KDM5B, NOTCH1, CEBPD, TWIST2, GATA3, RBPJ, TOB1, RASD1, MECOM, MYB, KAT8, PHC2, POU2F1, CBX3, XRCC5, ZBTB16, WWP1, FOXN3, KLF4, BMP7, FABP4, ELF3, BCL6, IRF1, BCL3, BHLHE40, ZNF438, RARA, PDCD4, ZNF777, TRIB3, LGR4, VGLL4*
Protein dephosphorylation	4.94E-04	*DUSP4, DUSP5, CTDP1, DUSP1, EYA2, CDC14B, PPM1G, PPP2CA, PTP4A1, PPP6C, PTP4A3, PPP4R1, PPP4R2, PPP2R1A*
Positive regulation of transcription from RNA polymerase II promoter	7.83E-04	*CSRNP1, CSF3, ZNF296, ADIRF, TCF20, UBE3A, RORA, RBPJ, IKBKB, MECOM, LRP5L, SOX15, NAMPT, MYB, CHP2, KPNA6, KAT8, HRAS, TEAD3, OSR2, LMO4, PAX6, RGMA, TNFRSF1A, MED25, TOX2, ELF3, IRF1, ZNF438, RARA, SQSTM1, ZNF750, ZNF395, CASZ1, NOTCH1, CEBPD, GATA3, HDAC8, ACVR1B, HIF1A, OTX1, JAG1, POU2F1, SMAD3, AUTS2, BCL11A, ZBTB16, FOXJ2, KLF4, BMP7, FOSL2, NFKBIA, DRAP1, MEIS1, BCL3*
Extracellular matrix organization	9.45E-04	*ERO1A, COL17A1, TNXB, MMP1, COL11A1, MMP3, FBLN2, SERPINB5, ADAMTS4, SPINT1, ELF3, BCL3, VWA1, ADAMTS9*
Cellular response to tumor necrosis factor	0.001015831	*CCL13, RORA, GATA3, YBX3, CLDN1, ASS1, NFKBIA, IKBKB, ZFP36, FABP4, LCN2, CYP1B1, TNFRSF21*
Regulation of cell differentiation	0.001022972	*PPP2CA, TNXB, CEBPD, BCL6, PPP2R1A, KLF4, RUNX3*
Negative regulation of MAPK cascade	0.001321732	*DUSP4, EFNA1, DUSP5, DUSP3, RNF149, DUSP1, PPP2R1A*
Epithelial cell differentiation	0.001478073	*UPK1B, KRT19, KRT17, KRT4, ELF3, PIH1D1, KRT15, KRT14, KRT13, BMP7*
Positive regulation of vascular	0.00148194	*C3, FLT4, CXCL17, CYP1B1, RORA, HIF1A*

endothelial growth factor production		
Keratinization	0.001586121	*TGM1, KRT17, KRT4, SFN, KRT5, CNFN, ABCA12, PPL, EVPL*
Negative regulation of growth	0.001822949	*MT2A, MT1A, MT1M, MT1X, HIF1A*
Positive regulation of transcription, DNA-templated	0.001837581	*CASZ1, NOTCH1, CITED4, RORA, GATA3, MRPL12, MED16, HIF1A, IKBKB, PDLIM1, MECOM, TP53INP1, MYB, PIM1, TNNI2, KAT8, OSR2, SMAD3, RBPMS, ACTN1, ZBTB16, FOXJ2, PAX6, MED4, KLF4, BMP7, RUNX3, IRF1, BCL3, JMY, RARA, WAC, TRIM16, UBE2V1, VGLL1, LGR4*
Keratinocyte differentiation	0.001901823	*TGM1, UGCG, NOTCH1, JAG1, KRT14, PAX6, RBPJ, EVPL*
Cell migration	0.002155807	*NTNG1, VAV3, LAMA5, ZRANB1, BTG1, LAMB3, SDC4, NDE1, PXN, IQGAP1, RND3, CDC42, EFNA1, CEACAM1, SH3BP1, RHOU, RAC1, CD44*
Neural tube closure	0.002373444	*VASP, SDC4, SPINT1, LMO4, DLC1, RARA, ADM, IFT57, RGMA*
Intermediate filament cytoskeleton organization	0.002690246	*FAM83H, KRT18, DST, PPL, EVPL*
Mesonephros development	0.003235213	*OSR2, ZBTB16, GATA3, BMP7*
Negative regulation of epithelial cell proliferation	0.003516554	*CDKN2B, NOTCH1, KRT4, MARVELD3, PAX6, IFT57, RUNX3, STK3*
Cell-matrix adhesion	0.003598847	*COL17A1, MKLN1, FERMT1, TNXB, BCL6, PXN, ZYX, RAC1, MSLN, CD44*
Negative regulation of ERK1 and ERK2 cascade	0.003690075	*DUSP4, ACE2, DUSP3, DUSP1, SPRY2, DMD, KLF4, EZR, EIF3A*
Response to retinoic acid	0.003914668	*GJB2, DUSP1, RARA, TRIM16, KLF4, AQP3, PTGES*
Transcription from RNA polymerase II promoter	0.004613737	*NOTCH1, CEBPD, PAX6, GATA3, KLF4, MED16, MED4, GTF2E2, TNFRSF1A, DRAP1, ELF3, BCL6, IRF1, KPNA6, SQSTM1, POLR2J*
Activation of MAPK activity	0.004817711	*ALK, DUSP5, GDF15, CXCL17, STK39, FPR1, MUC20, AVPI1*
Peptidyl-threonine dephosphorylation	0.005195357	*DUSP4, PPP2CA, DUSP5, DUSP1, PPM1G*
Cardiac septum morphogenesis	0.005373788	*NOTCH1, JAG1, BMP7, DHRS3*
Protein phosphorylation	0.005604469	*STK39, ACVR1B, STK3, IKBKB, CDC42, PPP4R1, STK38, PIM1, MAP3K6, MARK2, EIF2A, MAP3K1, LIMK2, RIPK4, PAX6, CSNK1E, RUNX3, GAK, OBSCN, STK24, RARA, PICK1, TRIB3, ALPK1, TRIB1, SQSTM1*
Response to lipopolysaccharide	0.005839931	*SELP, GJB2, NOTCH1, SLPI, FMO1, ADM, S100A14, TRIB1, CLDN1, S100A9, S100A8, PTGES*
Cellular oxidant detoxification	0.006451845	*UBIAD1, GPX2, GPX3, TP53INP1, GSR, HP, S100A9, PTGES*
Acute-phase response	0.006681022	*SERPINA3, IL1RN, HP, LCN2, SAA2, ASS1*
Cell proliferation	0.007107871	*BTG1, XRCC5, SPHK1, ZBTB16, ADM, GATA3, SDCBP2, FPGS, TFF1, TACC2, RAC1, PTGES, FAM83A*
Cell differentiation	0.007802019	*NOTCH1, TWIST2, CXCL17, ADIRF, RASAL1, AMBRA1, TYMP, FOXQ1, UGCG, ALKBH5, CSRP2, MECOM, SOX15, FLNC, HES4, EDARADD, OSR2, SMAD3, GADD45B, EYA2, NDE1, FOXJ2, SYT17, NUDT21, BMP1, ELF3, PPDPF, RARA, TFF1, UBE2V1, SQSTM1, ZNF750*

Inflammatory response	0.008270957	*SERPINA3, CCL13, IL1RN, TNFAIP6, IL1R1, SPHK1, STK39, FPR1, ADM, CXCL1, TNFRSF1A, SELP, C3, IKBKB, ELF3, BCL6, S100A12, ACKR1, RAC1, S100A9, CD44, S100A8*
Positive regulation of cell migration	0.009297061	*NOTCH1, SMAD3, TNFAIP6, SPHK1, FLT4, TWIST2, LAMC2, F3, CLDN1, CDC42, FAM83H, PTP4A1, SH3RF2, CEACAM6, SPRY2, HRAS*
Proteolysis	0.009402961	*CFD, FCN3, MMP1, HTRA3, MMP3, TYSND1, UBE3A, KLK11, KLK12, TMPRSS11D, ADAMTS4, BACE2, ACE2, PM20D2, BMP1, RELN, MASP1, PRSS8, CAPN1, TPSAB1, CTSD, ADAMTS9*
Cellular response to leukemia inhibitory factor	0.009434953	*KDM5B, PTP4A3, MAT2A, XRCC5, PIGA, SPRY2, PAX6, KLF4, BSPRY*
Detoxification of copper ion	0.009873042	*MT2A, MT1A, MT1M, MT1X*
Positive regulation of heterotypic cell-cell adhesion	0.009873042	*CEACAM6, FLOT1, BMP7, CD44*

MAPK, mitogen-activated protein kinase.
