# Supplementary material for: Novel potential drugs for the treatment of primary open-angle glaucoma using protein-protein interaction network analysis
Source: Genomics Inform. 2023 Mar 31;21(1):e6. doi: 10.5808/gi.22070 (PMC10085733; doi:10.5808/gi.22070)
Supplement: Supplementary Table 10. — Reactome pathway results for protein-protein interaction module 1 [file gi-22070-Supplementary-Table-10.pdf]

Supplementary Table 10. Reactome pathway results for protein-protein interaction module 1

Reactome pathway	p-value	Genes
Deubiquitination	7.57E-07	*USP7*, *ZRANB1*, *UBB*, *STAMBPL1*, *PSMC1*, *RAD23A*
Degradation of beta-catenin by the destruction complex	0.001544214	*ZRANB1*, *UBB*, *PSMC1*
Post-translational protein modification	0.001556859	*USP7*, *ZRANB1*, *UBB*, *STAMBPL1*, *PSMC1*, *RAD23A*
Nucleotide excision repair	0.002742056	*USP7*, *UBB*, *RAD23A*
PTEN regulation	0.004324011	*USP7*, *UBB*, *PSMC1*
Metabolism of proteins	0.006491553	*USP7*, *ZRANB1*, *UBB*, *STAMBPL1*, *PSMC1*, *RAD23A*
Regulation of PTEN localization	0.006560939	*USP7*, *UBB*
Josephin domain DUBs	0.008739533	*UBB*, *RAD23A*
