# Supplementary material for: Novel potential drugs for the treatment of primary open-angle glaucoma using protein-protein interaction network analysis
Source: Genomics Inform. 2023 Mar 31;21(1):e6. doi: 10.5808/gi.22070 (PMC10085733; doi:10.5808/gi.22070)
Supplement: Supplementary Table 11. — Reactome pathway results for protein-protein interaction module 2 [file gi-22070-Supplementary-Table-11.pdf]

Supplementary Table 11. Reactome pathway results for protein-protein interaction module 2

Reactome pathway	p-value	Genes
Complex I biogenesis	7.09E-17	*NDUFB8*, *TIMMDC1*, *NDUFB10*, *NDUFB5*, *NDUFB2*, *NDUFC1*, *TMEM126B*, *NDUFV2*
Respiratory electron transport	5.31E-15	*NDUFB8*, *TIMMDC1*, *NDUFB10*, *NDUFB5*, *NDUFB2*, *NDUFC1*, *TMEM126B*, *NDUFV2*
Respiratory electron transport, ATP synthesis by chemiosmotic coupling, and heat production by uncoupling proteins	2.40E-14	*NDUFB8*, *TIMMDC1*, *NDUFB10*, *NDUFB5*, *NDUFB2*, *NDUFC1*, *TMEM126B*, *NDUFV2*
Citric acid cycle and respiratory electron transport	2.67E-13	*NDUFB8*, *TIMMDC1*, *NDUFB10*, *NDUFB5*, *NDUFB2*, *NDUFC1*, *TMEM126B*, *NDUFV2*
Metabolism	1.01E-05	*NDUFB8*, *TIMMDC1*, *NDUFB10*, *NDUFB5*, *NDUFB2*, *NDUFC1*, *TMEM126B*, *NDUFV2*
