# Supplementary material for: Novel potential drugs for the treatment of primary open-angle glaucoma using protein-protein interaction network analysis
Source: Genomics Inform. 2023 Mar 31;21(1):e6. doi: 10.5808/gi.22070 (PMC10085733; doi:10.5808/gi.22070)
Supplement: Supplementary Table 12. — Reactome pathway results for protein-protein interaction module 3 [file gi-22070-Supplementary-Table-12.pdf]

Supplementary Table 12. Reactome pathway results for protein-protein interaction module 3

Reactome pathway	p-value	Genes
Translation	1.00E-11	*RPL32*, *EIF3L*, *RPS5*, *SEC61G*, *RPS27L*, *RPL15*, *EIF3A*, *SRP9*, *RPS12*
Formation of a pool of free 40S subunits	1.15E-10	*RPL32*, *EIF3L*, *RPS5*, *RPS27L*, *RPL15*, *EIF3A*, *RPS12*
L13a-mediated translational silencing of ceruloplasmin expression	2.04E-10	*RPL32*, *EIF3L*, *RPS5*, *RPS27L*, *RPL15*, *EIF3A*, *RPS12*
SRP-dependent cotranslational protein targeting to membrane	2.15E-10	*RPL32*, *RPS5*, *SEC61G*, *RPS27L*, *RPL15*, *SRP9*, *RPS12*
GTP hydrolysis and joining of the 60S ribosomal subunit	2.15E-10	*RPL32*, *EIF3L*, *RPS5*, *RPS27L*, *RPL15*, *EIF3A*, *RPS12*
Cap-dependent translation Initiation	3.10E-10	*RPL32*, *EIF3L*, *RPS5*, *RPS27L*, *RPL15*, *EIF3A*, *RPS12*
Eukaryotic translation initiation	3.10E-10	*RPL32*, *EIF3L*, *RPS5*, *RPS27L*, *RPL15*, *EIF3A*, *RPS12*
Major pathway of rRNA processing in the nucleolus and cytosol	4.27E-09	*TBL3*, *RPL32*, *IMP3*, *RPS5*, *RPS27L*, *RPL15*, *RPS12*
rRNA processing in the nucleus and cytosol	5.86E-09	*TBL3*, *RPL32*, *IMP3*, *RPS5*, *RPS27L*, *RPL15*, *RPS12*
rRNA processing	7.92E-09	*TBL3*, *RPL32*, *IMP3*, *RPS5*, *RPS27L*, *RPL15*, *RPS12*
Formation of the ternary complex and subsequently the 43S complex	9.31E-08	*EIF3L*, *RPS5*, *RPS27L*, *EIF3A*, *RPS12*
Translation initiation complex formation	1.56E-07	*EIF3L*, *RPS5*, *RPS27L*, *EIF3A*, *RPS12*
Ribosomal scanning and start codon recognition	1.56E-07	*EIF3L*, *RPS5*, *RPS27L*, *EIF3A*, *RPS12*
Activation of the mRNA upon binding of the cap-binding complex and eIFs, and subsequent binding to 43S	1.67E-07	*EIF3L*, *RPS5*, *RPS27L*, *EIF3A*, *RPS12*
Viral mRNA translation	8.64E-07	*RPL32*, *RPS5*, *RPS27L*, *RPL15*, *RPS12*
Peptide chain elongation	8.64E-07	*RPL32*, *RPS5*, *RPS27L*, *RPL15*, *RPS12*
Eukaryotic translation Termination	1.03E-06	*RPL32*, *RPS5*, *RPS27L*, *RPL15*, *RPS12*
Eukaryotic translation Elongation	1.03E-06	*RPL32*, *RPS5*, *RPS27L*, *RPL15*, *RPS12*
Selenocysteine synthesis	1.03E-06	*RPL32*, *RPS5*, *RPS27L*, *RPL15*, *RPS12*
NMD independent of the EJC	1.12E-06	*RPL32*, *RPS5*, *RPS27L*, *RPL15*, *RPS12*
Response of EIF2AK4 (GCN2) to amino acid deficiency	1.43E-06	*RPL32*, *RPS5*, *RPS27L*, *RPL15*, *RPS12*
NMD enhanced by the EJC	2.39E-06	*RPL32*, *RPS5*, *RPS27L*, *RPL15*, *RPS12*
NMD	2.39E-06	*RPL32*, *RPS5*, *RPS27L*, *RPL15*, *RPS12*
Selenoamino acid metabolism	2.65E-06	*RPL32*, *RPS5*, *RPS27L*, *RPL15*, *RPS12*
Influenza viral RNA transcription and replication	4.52E-06	*RPL32*, *RPS5*, *RPS27L*, *RPL15*, *RPS12*

Cellular response to starvation	8.00E-06	*RPL32, RPS5, RPS27L, RPL15, RPS12*
Influenza infection	8.00E-06	*RPL32, RPS5, RPS27L, RPL15, RPS12*
Regulation of expression of Slits and Robos	1.15E-05	*RPL32, RPS5, RPS27L, RPL15, RPS12*
Metabolism of RNA	1.28E-05	*TBL3, RPL32, IMP3, RPS5, RPS27L, RPL15, RPS12*
Signaling by Robo receptors	2.98E-05	*RPL32, RPS5, RPS27L, RPL15, RPS12*
Metabolism of proteins	3.46E-05	*RPL32, EIF3L, RPS5, SEC61G, RPS27L, RPL15, EIF3A, SRP9, RPS12*
Metabolism of amino acids and derivatives	2.44E-04	*RPL32, RPS5, RPS27L, RPL15, RPS12*
SARS-CoV-1 modulates host translation machinery	4.92E-04	*RPS5, RPS27L, RPS12*
SARS-CoV-2 modulates host translation machinery	9.35E-04	*RPS5, RPS27L, RPS12*
Axon guidance	0.00106	*RPL32, RPS5, RPS27L, RPL15, RPS12*
Nervous system development	0.001251	*RPL32, RPS5, RPS27L, RPL15, RPS12*
SARS-CoV-1-host interactions	0.003272	*RPS5, RPS27L, RPS12*
Cellular responses to stress	0.003957	*RPL32, RPS5, RPS27L, RPL15, RPS12*
Cellular responses to stimuli	0.004219	*RPL32, RPS5, RPS27L, RPL15, RPS12*
SARS-CoV-1 infection	0.007024	*RPS5, RPS27L, RPS12*
Infectious disease	0.008026	*RPL32, RPS5, RPS27L, RPL15, RPS12*

NMD, nonsense-mediated decay; EJC, exon junction complex; SARS-CoV-1, severe acute respiratory syndrome coronavirus 1; SARS-CoV-2, severe acute respiratory syndrome coronavirus 2.
