# Supplementary material for: Novel potential drugs for the treatment of primary open-angle glaucoma using protein-protein interaction network analysis
Source: Genomics Inform. 2023 Mar 31;21(1):e6. doi: 10.5808/gi.22070 (PMC10085733; doi:10.5808/gi.22070)
Supplement: Supplementary Table 13. — Biological process results for protein-protein interaction module 1 [file gi-22070-Supplementary-Table-13.pdf]

Supplementary Table 13. Biological process results for protein-protein interaction module 1

Biological process	p-value	Genes
Protein K63-linked deubiquitination	1.01E-04	*USP7*, *ZRANB1*, *STAMBPL1*
