# Supplementary material for: Novel potential drugs for the treatment of primary open-angle glaucoma using protein-protein interaction network analysis
Source: Genomics Inform. 2023 Mar 31;21(1):e6. doi: 10.5808/gi.22070 (PMC10085733; doi:10.5808/gi.22070)
Supplement: Supplementary Table 14. — Cell component results for protein-protein interaction module 1 [file gi-22070-Supplementary-Table-14.pdf]

Supplementary Table 14. Cell component results for protein-protein interaction module 1

Cell component	p-value	Genes
Cytosol	1.83E-04	*USP7*, *ZRANB1*, *UBB*, *STAMBPL1*, *PSMC1*, *TOM1*, *UBQLN1*, *RAD23A*, *VPS36*
Proteasome complex	2.68E-04	*PSMC1*, *UBQLN1*, *RAD23A*
Nucleoplasm	0.002483061	*USP7*, *ZRANB1*, *UBB*, *PSMC1*, *UBQLN1*, *RAD23A*, *CCDC92*
Endosome	0.007975452	*STAMBPL1*, *TOM1*, *VPS36*
