# Supplementary material for: Novel potential drugs for the treatment of primary open-angle glaucoma using protein-protein interaction network analysis
Source: Genomics Inform. 2023 Mar 31;21(1):e6. doi: 10.5808/gi.22070 (PMC10085733; doi:10.5808/gi.22070)
Supplement: Supplementary Table 15. — Molecular function results for protein-protein interaction module 1 [file gi-22070-Supplementary-Table-15.pdf]

Supplementary Table 15. Molecular function results for protein-protein interaction module 1

Molecular function	p-value	Genes
Polyubiquitin binding	4.43E-07	*ZRANB1*, *TOM1*, *UBQLN1*, *RAD23A*
Ubiquitin binding	8.79E-04	*TOM1*, *RAD23A*, *VPS36*
Thiol-dependent ubiquitin-specific protease activity	0.001373767	*USP7*, *ZRANB1*, *STAMBPL1*
Ubiquitinyl hydrolase activity	0.005224814	*USP7*, *ZRANB1*
Protein C-terminus binding	4.43E-07	*ZRANB1*, *TOM1*, *UBQLN1*, *RAD23A*
