# Supplementary material for: Novel potential drugs for the treatment of primary open-angle glaucoma using protein-protein interaction network analysis
Source: Genomics Inform. 2023 Mar 31;21(1):e6. doi: 10.5808/gi.22070 (PMC10085733; doi:10.5808/gi.22070)
Supplement: Supplementary Table 16. — Biological process results for protein-protein interaction module 2 [file gi-22070-Supplementary-Table-16.pdf]

Supplementary Table 16. Biological process results for protein-protein interaction module 2

Biological process	p-value	Genes
Mitochondrial electron transport, NADH to ubiquinone	4.08E-13	*NDUFB8*, *NDUFB10*, *NDUFB5*, *NDUFB2*, *NDUFC1*, *NDUFV2*
Mitochondrial respiratory chain complex I assembly	1.87E-12	*NDUFB8*, *NDUFB10*, *NDUFB5*, *NDUFB2*, *NDUFC1*, *TMEM126B*
Mitochondrial ATP synthesis coupled proton transport	2.20E-12	*NDUFB8*, *NDUFB10*, *NDUFB5*, *NDUFB2*, *NDUFC1*, *NDUFV2*
Aerobic respiration	3.22E-12	*NDUFB8*, *NDUFB10*, *NDUFB5*, *NDUFB2*, *NDUFC1*, *NDUFV2*
