# Supplementary material for: Novel potential drugs for the treatment of primary open-angle glaucoma using protein-protein interaction network analysis
Source: Genomics Inform. 2023 Mar 31;21(1):e6. doi: 10.5808/gi.22070 (PMC10085733; doi:10.5808/gi.22070)
Supplement: Supplementary Table 17. — Cell component results for protein-protein interaction module 2 [file gi-22070-Supplementary-Table-17.pdf]

Supplementary Table 17. Cell component results for protein-protein interaction module 2

Cell component	p-value	Genes
Mitochondrial respiratory chain complex I	1.44E-12	*NDUFB8*, *NDUFB10*, *NDUFB5*, *NDUFB2*, *NDUFC1*, *NDUFV2*
Mitochondrial inner membrane	3.54E-12	*NDUFB8*, *TIMMDC1*, *NDUFB10*, *NDUFB5*, *NDUFB2*, *NDUFC1*, *TMEM126B*, *NDUFV2*
Mitochondrion	7.24E-04	*NDUFB8*, *TIMMDC1*, *NDUFB5*, *TMEM126B*, *NDUFV2*
