# Supplementary material for: Novel potential drugs for the treatment of primary open-angle glaucoma using protein-protein interaction network analysis
Source: Genomics Inform. 2023 Mar 31;21(1):e6. doi: 10.5808/gi.22070 (PMC10085733; doi:10.5808/gi.22070)
Supplement: Supplementary Table 18. — Molecular function results for protein-protein interaction module 2 [file gi-22070-Supplementary-Table-18.pdf]

Supplementary Table 18. Molecular function results for protein-protein interaction module 2

Molecular function	p-value	Genes
NADH dehydrogenase (ubiquinone) activity	1.00E-12	ND*UFB8, NDUFB10*, *NDUFB5, NDUFB2*, *NDUFC1*, *NDUFV2*
