# Supplementary material for: Novel potential drugs for the treatment of primary open-angle glaucoma using protein-protein interaction network analysis
Source: Genomics Inform. 2023 Mar 31;21(1):e6. doi: 10.5808/gi.22070 (PMC10085733; doi:10.5808/gi.22070)
Supplement: Supplementary Table 19. — Biological process results for protein-protein interaction module 3 [file gi-22070-Supplementary-Table-19.pdf]

Supplementary Table 19. Biological process results for protein-protein interaction module 3

Biological process	p-value	Genes
Translation	5.34E-06	*RPL32, RPS5, RPS27L, RPL15, RPS12*
Cytoplasmic translation	1.57E-05	*RPL32, RPS5, RPL15, RPS12*
Translational initiation	3.99E-04	*EIF3L, RPS5, EIF3A*
Viral translational termination-reinitiation	0.002842	*EIF3L, EIF3A*
Ribosomal small subunit assembly	0.008504	*RPS5, RPS27L*
Formation of cytoplasmic translation initiation complex	0.009633	*EIF3L, EIF3A*
