# Supplementary material for: Novel potential drugs for the treatment of primary open-angle glaucoma using protein-protein interaction network analysis
Source: Genomics Inform. 2023 Mar 31;21(1):e6. doi: 10.5808/gi.22070 (PMC10085733; doi:10.5808/gi.22070)
Supplement: Supplementary Table 20. — Cell component results for protein-protein interaction module 3 [file gi-22070-Supplementary-Table-20.pdf]

Supplementary Table 20. Cell component results for protein-protein interaction module 3

Cell component	p-value	Genes
Cytosolic ribosome	1.08E-05	*RPL32*, *RPS5*, *RPL15*, *RPS12*
Ribosome	1.28E-04	*RPL32*, *RPS5*, *RPS27L*, *RPL15*
Cytosolic small ribosomal subunit	3.18E-04	*RPS5*, *RPS27L*, *RPS12*
Cytosol	0.004484	*RPL32, EIF3L, RPS5, SEC61G, ZNF622, RPL15, EIF3A, SRP9, RPS12*
Eukaryotic 48S preinitiation complex	0.009292	*EIF3L*, *EIF3A*
Eukaryotic translation initiation factor 3 complex	0.009292	*EIF3L*, *EIF3A*
