# Supplementary material for: Novel potential drugs for the treatment of primary open-angle glaucoma using protein-protein interaction network analysis
Source: Genomics Inform. 2023 Mar 31;21(1):e6. doi: 10.5808/gi.22070 (PMC10085733; doi:10.5808/gi.22070)
Supplement: Supplementary Table 21. — Molecular function results for protein-protein interaction module 3 [file gi-22070-Supplementary-Table-21.pdf]

Supplementary Table 21. Molecular function results for protein-protein interaction module 3

Molecular function	p-value	Genes
RNA binding	4.50E-10	*TBL3*, *RPL32*, *IMP3*, *EIF3L*, *RPS5*, *RPS27L*, *ZNF622*, *RPL15*, *EIF3A*, *SRP9*, *RPS12*
Structural constituent of ribosome	4.50E-06	*RPL32*, *RPS5*, *RPS27L*, *RPL15*, *RPS12*
